# SNPs within microRNA binding sites and the prognosis of breast cancer

**DOI:** 10.18632/aging.202612

**Published:** 2021-02-26

**Authors:** Liwen Zhang, Lu Han, Yubei Huang, Ziwei Feng, Xin Wang, Haixin Li, Fangfang Song, Luyang Liu, Junxian Li, Hong Zheng, Peishan Wang, Fengju Song, Kexin Chen

**Affiliations:** 1 Department of Epidemiology and Biostatistics, Key Laboratory of Molecular Cancer Epidemiology of Tianjin, National Clinical Research Center for Cancer, Tianjin Medical University Cancer Institute and Hospital, Tianjin 300060, People’s Republic of China; 2 Department of Infection Control, Tianjin Huanhu Hospital, Tianjin 300350, People’s Republic of China; 3 Department of Epidemiology and Biostatistics, West China School of Public Health, Sichuan University, Sichuan 610041, People’s Republic of China; 4 Department of Cancer Biobank, Key Laboratory of Cancer Prevention and Therapy of Tianjin, Tianjin’s Clinical Research Center for Cancer, National Clinical Research Centre of Cancer, Tianjin Medical University Cancer Institute and Hospital, Tianjin 300060, People’s Republic of China

**Keywords:** breast cancer, microRNA, single nucleotide polymorphism, prognosis

## Abstract

Single nucleotide polymorphisms (SNPs) within microRNA binding sites can affect the binding of microRNA to mRNA and regulate gene expression, thereby contributing to cancer prognosis. Here we performed a two-stage study of 2647 breast cancer patients to explore the association between SNPs within microRNA binding sites and breast cancer prognosis. In stage I, we genotyped 192 SNPs within microRNA binding sites using the Illumina Goldengate platform. In stage II, we validated SNPs associated with breast cancer prognosis in another dataset using the TaqMan platform. We identified 8 SNPs significantly associated with breast cancer prognosis in stage I (*P*<0.05), and only rs10878441 was statistically significant in stage II (AA vs CC, HR=2.21, 95% CI: 1.11-4.42, *P*=0.024). We combined the data from stage I and stage II, and found that, compared with rs10878441 AA genotype, CC genotype was associated with poor survival of breast cancer (HR=2.19, 95% CI: 1.30-3.70, *P*=0.003). Stratified analyses demonstrated that rs10878441 was related to breast cancer prognosis in grade II and lymph node-negative patients (*P*<0.05). The Leucine-rich repeat kinase 2 (LRRK2) rs10878441 CC genotype is associated with poor prognosis of breast cancer in a Chinese population and may be used as a potential prognostic biomarker for breast cancer.

• The LRRK2 rs10878441 CC genotype is associated with poor prognosis of breast cancer in a Chinese population.

• Stratified analyses demonstrated that rs10878441 was related to breast cancer prognosis in grade II patients and lymph node-negative patients.

## INTRODUCTION

Breast cancer is the most commonly diagnosed tumor and the leading cause of cancer death among women, with an estimated 2.1 million new cases and 626,679 deaths worldwide each year according to the Globocan 2018 [1]. In China, breast cancer is predicted to account for about 15% of all new cancer cases among women [2]. It is estimated that around 3-6 million SNPs in the human genome could provide a means for elucidating the genetic component of complex diseases [3].

For many years, age at diagnosis, axillary lymph node metastasis, tumor size, histological grade, hormone receptor status, and human epidermal growth factor receptor 2 (HER2) status represented principal factors used for the purposes of evaluating the prognosis and determining the appropriate strategy of treatment [4]. In addition, different environmental exposures can lead to different prognosis of breast cancer. Body mass index (BMI), nutrition and physical activity are related to the prognosis of breast cancer [5, 6]. Reproductive factors such as breastfeeding and pregnancy have been reported to be associated with breast cancer prognosis [7, 8].

MicroRNAs (miRNAs) are endogenous non-coding small RNAs (containing about 22 nucleotides) that regulate gene expression by Waston-Crick pairing with the target gene of the 3’ untranslated region (3’UTR). It has been reported that microRNAs regulate nearly 30% of human genes [9], and play important roles in most physiological and pathological processes such as tumorigenesis and proliferation. The binding of microRNA to mRNA is critical for regulating the mRNA level and protein expression. However, this binding can be affected by SNPs that reside in the microRNA binding sites. Therefore, SNP variations may interfere or disrupt the binding of the SNPs to microRNAs, which may affect the regulation of miRNAs on target genes, thereby contributing to the prognosis of cancer [10–12].

In recent years, a number of studies have reported a link between SNPs within microRNA binding sites and prognosis of various types of cancer including breast cancer [12–14]. Teo et al [15] reported the role of rs7180135 in RAD51 in the prognosis of breast cancer patients, and the G minor allele had improved breast cancer specific survival. Brendle et al [16] identified that the A allele of the SNP rs743554 in the 3’UTR of ITGB4 gene was associated with estrogen receptor-negative tumors and worse survival in patients with breast cancer. Zhang et al [17] found that miR-367-binding site rs1044129 in RYR3 gene was associated with poor survival of patients with breast cancer. Liu et al [18] uncovered that TT genotype of rs16917496 on SET8 3′-UTR region was significantly associated with poor outcome of breast cancer in a Chinese population.

However, there is still a lack of association studies between SNPs within microRNA binding sites and the prognosis of breast cancer with large sample size in China. Therefore, we carried out a two-stage cohort study to investigate the relationship between SNPs within microRNA binding sites and breast cancer prognosis.

## RESULTS

### Demographic and epidemiological characteristics of patients

The demographic and epidemiological characteristics of 2647 breast cancer patients were shown in Table 1. The median age at diagnosis of all patients was 51 years (range 22-89). The median follow-up time was 68 months (range 0-159). 302 (12.0%) patients smoked and 63 (2.6%) patients drank alcohol. 1385 (52.6%) patients had menopause, 686 (26.4%) patients had benign breast disease, and 814 (30.9%) patients had a family history of cancer. In total, 239 patients died and 335 patients displayed tumor progression. Univariate analysis showed that age at diagnosis, education, occupation, age at menarche, number of live births, breastfeeding duration, abortion and menopause were significantly associated with breast cancer OS (*P*<0.05). In addition, age at diagnosis, number of live births, breastfeeding duration, abortion, menopause, and BBD were significantly related to breast cancer DFS (*P*<0.05).

**Table 1:** Demographic and epidemiological characteristics of breast cancer patients and associations with breast cancer prognosis.

**Characteristics**	***N*=2647**	**Stage I**	**Stage II**	**Overall survival**			**Disease-free survival**	
	**(%)**	**(*N*=1297, %)**	**(*N*=1350, %)**	**HR (95% CI)**	***P***		**HR (95% CI)**	***P***
**Age at diagnosis (years)**								
≤50	1167 (44.1)	578 (44.6)	589 (43.7)	1 (ref)	**<0.001**		1 (ref)	**0.016**
>50	1477 (55.9)	717 (55.4)	760 (56.3)	**1.655 (1.264-2.168)**			**1.320 (1.052-1.657)**	
**BMI (kg/m**^2^**)**								
≤18.4	56 (2.1)	30 (2.4)	26 (1.9)	0.900 (0.330-2.457)	**0.203**		0.718 (0.293-1.759)	**0.634**
18.5-23.9	1025 (39.3)	512 (40.4)	513 (38.4)	1 (ref)			1 (ref)	
24.0-27.9	1024 (39.3)	508 (40.1)	516 (38.6)	1.291 (0.962-1.733)			1.105 (0.858-1.423)	
≥28	500 (19.2)	218 (17.2)	283 (21.1)	1.386 (0.975-1.971)			1.142 (0.838-1.556)	
Marital status								
Unmarried	35 (1.3)	16 (1.2)	19 (1.4)	1 (ref)	0.593		1 (ref)	0.850
Married	2420 (92.9)	1180 (91.8)	1240 (93.9)	0.939 (0.300-2.934)			1.186 (0.380-3.701)	
Divorced/widowed	150 (5.8)	89 (6.9)	61 (4.6)	1.213 (0.355-4.139)			1.329 (0.393-4.493)	
Education								
Without education	133 (5.3)	71 (5.7)	62 (5.0)	1 (ref)	**0.001**		1 (ref)	0.079
Primary school	303 (12.1)	154 (12.3)	149 (12.0)	0.615 (0.357-1.056)			0.656 (0.386-1.116)	
Junior high school	738 (29.6)	364 (29.1)	374 (30.1)	**0.399 (0.243-0.658)**			0.563 (0.352-0.902)	
High school	810 (32.5)	426 (34.0)	384 (30.9)	**0.425 (0.261-0.693)**			0.545 (0.342-0.869)	
College and advanced	512 (20.5)	238 (19.0)	274 (22.0)	0.372 (0.218-0.634)			0.520 (0.318-0.852)	
Average monthly income (RMB)								
≤999	839 (34.5)	480 (38.8)	359 (30.0)	1 (ref)	0.056		1 (ref)	0.112
1000-1999	1055 (43.3)	544 (43.9)	511 (42.8)	0.895 (0.667-1.202)			0.995 (0.766-1.293)	
≥2000	539 (22.2)	214 (17.3)	325 (27.2)	0.616 (0.414-0.919)			0.718 (0.510-1.012)	
Occupation								
No	1459 (58.3)	770 (61.3)	689 (55.3)	1 (ref)	**0.009**		1 (ref)	0.089
Yes	1042 (41.7)	486 (38.7)	556 (44.7)	**0.689 (0.520-0.912)**			0.814 (0.641-1.033)	
Age at marriage (years)								
<30	2441 (93.9)	1202 (94.1)	1239 (93.8)	1 (ref)	**0.816**		1 (ref)	**0.457**
≥30	158 (6.1)	76 (5.9)	82 (6.2)	0.940 (0.556-1.587)			0.831 (0.509-1.355)	
Age at menarche (years)								
≤14	698 (26.6)	347 (27.0)	351 (26.2)	1 (ref)	**0.015**		1 (ref)	0.117
>14	1927 (73.4)	938 (73.0)	989 (73.8)	**1.466 (1.075-1.999)**			1.231 (0.949-1.596)	
Number of pregnancies								
≤2	1198 (45.5)	576 (44.7)	622 (46.2)	1 (ref)	0.385		1 (ref)	0.695
>2	1435 (54.5)	712 (55.3)	723 (53.8)	1.120 (0.867-1.448)			1.045 (0.837-1.305)	
Number of live births								
≤1	1585 (62.0)	783 (62.4)	802 (61.6)	1 (ref)	<0.001		1 (ref)	0.002
>1	971 (38.0)	471 (37.6)	500 (38.4)	**1.760 (1.358-2.280)**			**1.435 (0.143-1.802)**	
Breastfeeding duration (months)								
≤12	1045 (42.0)	534 (43.9)	511 (40.1)	1 (ref)	**0.028**		1 (ref)	**0.010**
>12	1444 (58.0)	682 (56.1)	762 (59.9)	**1.366 (1.033-1.804)**			**1.372 (1.079-1.746)**	
Abortion								
No	732 (28.2)	357 (28.1)	375 (28.2)	1 (ref)	0.003		1 (ref)	0.004
Yes	1867 (71.8)	914 (71.9)	953 (71.8)	**0.664 (0.508-0.868)**			**0.705 (0.556-0.893)**	
Oral contraceptive								
No	2036 (82.6)	1007 (81.5)	1029 (83.7)	1 (ref)	**0.823**		1 (ref)	**0.673**
Yes	428 (17.4)	228 (18.5)	200 (16.3)	1.040 (0.738-1.465)			0.935 (0.684-1.278)	
Menopause								
No	1247 (47.4)	614 (47.6)	633 (47.2)	1 (ref)	<0.001		1 (ref)	0.011
Yes	1385 (52.6)	677 (52.4)	708 (52.8)	**1.844 (1.408-2.417)**			**1.341 (1.070-1.679)**	
BBD								
No	1909 (73.6)	957 (74.2)	952 (72.9)	1 (ref)	0.227		1 (ref)	**0.028**
Yes	686 (26.4)	332 (25.8)	354 (27.1)	0.829 (0.611-1.125)			**0.741 (0.566-0.968)**	
Smoking								
No	2214 (88.0)	1108 (88.2)	1106 (87.8)	1 (ref)	**0.988**		1 (ref)	**0.428**
Yes	302 (12.0)	148 (11.8)	154 (12.2)	1.003 (0.671-1.500)			1.151 (0.812-1.632)	
Alcohol drinking								
No	2445 (97.4)	1233 (98.0)	1212 (96.8)	1 (ref)	**0.573**		1 (ref)	**0.246**
Yes	65 (2.6)	25 (2.0)	40 (3.2)	0.574 (0.280-2.025)			0.562 (0.209-1.508)	
Physical activity per week (hours)								
≤3	1817 (72.9)	943 (75.3)	874 (70.5)	1 (ref)	0.072		1 (ref)	0.067
>3	675 (27.1)	310 (24.7)	365 (29.5)	1.295 (0.976-1.718)			1.261 (0.984-1.617)	
Family history of cancer								
No	1817 (69.1)	912 (70.4)	905 (67.8)	1 (ref)	0.117		1 (ref)	0.258
Yes	814 (30.9)	384 (29.6)	430 (32.2)	0.795 (0.596-1.060)			0.868 (0.678-1.110)	

BMI, body mass index; BBD, benign breast disease; HR, hazard ratio; CI, confidence interval.

### Clinicopathological characteristics of patients

The clinicopathological characteristics of all participants were presented in Table 2. 1593 (67.7%) patients showed 0-IIa TNM stage and 761 (32.3%) patients showed IIb-IV TNM stage. There were 1483 (67.8%) patients with tumor size ≤2.5cm, 1853 (70.1%) patients with invasive ductal cancer, 567 (21.8%) patients with positive lymph nodes, 1542 (60.0%) patients with positive ER, 1383 (53.8%) patients with positive PR, and 555 (23.3%) patients with positive HER2. Univariate analysis showed that TNM stage, tumor size, histopathologic classification, grade, lymph node, ER, PR, and HER2 were significantly associated with breast cancer OS and DFS (*P*<0.05).

**Table 2:** Clinicopathological characteristics of breast cancer patients and associations with breast cancer prognosis.

**Characteristics**	***N*=2647**	**Stage I**	**Stage II**	**Overall survival**			**Disease-free survival**	
	**(%)**	**(*N*=1297, %)**	**(*N*=1350, %)**	**HR (95% CI)**	***P***		**HR (95% CI)**	***P***
TNM stage								
0-IIa	1593 (67.7)	772 (65.5)	821 (69.9)	1 (ref)	<0.001		1 (ref)	**<0.001**
IIb-IV	761 (32.3)	407 (34.5)	354 (30.1)	**3.493 (2.672-4.568)**			**2.776 (2.203-3.498)**	
Tumor size								
≤2.5cm	1483 (67.8)	683 (65.2)	800 (70.2)	1 (ref)	<0.001		1 (ref)	<0.001
>2.5cm	704 (32.2)	364 (34.8)	340 (29.8)	**2.092 (1.591-2.750)**			**1.694 (1.291-2.221)**	
Histopathologic classification								
non-IDC	789 (29.9)	449.(34.7)	340 (25.2)	1 (ref)	**<0.001**		1 (ref)	**<0.001**
IDC	1853 (70.1)	846 (65.3)	1007 (74.8)	**1.815 (1.324-2.487)**			**1.629 (1.275-2.082)**	
Grade								
I	187 (10.0)	74 (8.1)	113 (11.7)	1 (ref)	**0.015**		1 (ref)	**0.004**
II	1390 (74.0)	693 (76.0)	697 (72.2)	1.814 (0.954-3.449)			**2.903 (1487-5.668)**	
III	301 (16.0)	145 (15.9)	156 (16.1)	**2.593 (1.294-5.198)**			**2.934 (1.421-6.056)**	
Lymph node								
Negative	2029 (78.2)	983 (77.2)	1046 (79.1)	1 (ref)	**<0.001**		1 (ref)	**<0.001**
Positive	567 (21.8)	290 (22.8)	277 (20.9)	**4.488 (3.466-5.813)**			**3.672 (2.934-4.597)**	
ER								
Negative	1028 (40.0)	576 (44.9)	452 (35.1)	1 (ref)	<0.001		1 (ref)	**0.002**
Positive	1542 (60.0)	707 (55.1)	835 (64.9)	**0.589 (0.456-0.761)**			**0.701 (0.561-0.877)**	
PR								
Negative	1187 (46.2)	563 (43.9)	624 (48.5)	1 (ref)	<0.001		1 (ref)	**<0.001**
Positive	1383 (53.8)	720 (56.1)	662 (51.5)	**0.519 (0.399-0.675)**			**0.599 (0.478-0.751)**	
HER2								
Negative	1824 (76.7)	910 (79.3)	914 (74.2)	1 (ref)	**0.003**		1 (ref)	**0.018**
Positive	555 (23.3)	237 (20.7)	318 (25.8)	**1.544 (1.157-2.062)**			**1.365 (1.055-1.767)**	

ER, estrogen receptor; PR, progestogen receptor; HER2, human epidermal growth factor receptor 2; non-IDC, non-invasive ductal carcinoma; IDC, invasive ductal carcinoma; HR, hazard ratio; CI, confidence interval.

### Association between 192 SNPs and breast cancer prognosis in stage I

In stage I, the median follow-up time was 76 months (range 0 to 159). The relationship between 192 SNPs within microRNA binding sites and breast cancer OS were shown in Supplementary Table 2. Among the 192 candidate SNPs, 8 SNPs within microRNA binding sites were related to breast cancer OS (P<0.05), with and without adjustments for age at diagnosis, education, occupation, age at menarche, number of live births, breastfeeding duration, abortion, menopause, TNM stage, tumor size, histopathologic classification, grade, lymph node, ER, PR, and HER2 (Table 3 and Supplementary Figure 1). The associated SNPs were rs1053739 located in NMT1 at 17q21.31, rs2693 located in KIF13B at 8p12, rs698761 located in PREPL at 2p21, rs8602 located in MKNK1 at 1p33, rs10878441 located in LRRK2 at 12q12, rs10318 located in GREM1 at 15q13.3, rs10075853 located in ST8SIA4 at 5q21.1 and rs8410 located in PREPL at 2p21. We further analyzed the association between the 8 SNPs and breast cancer DFS, rs1053739, rs698761, rs10878441, rs10318, and rs8410 showed a significant association with breast cancer DFS (*P*<0.05) (Table 3 and Supplementary Figure 2).

**Table 3:** Association between SNP within microRNA binding sites and the prognosis of breast cancer (Stage I).

**SNP**	**Overall survival**						**Disease-free survival**				
	***N***	**HR (95% CI)**	***P***	**HR (95% CI)**	***P***^#^		***N***	**HR (95% CI)**	***P***	**HR (95% CI)**	P*
rs1053739											
AA	359	1 (ref)		1 (ref)			354	1 (ref)		1 (ref)	
AG	659	1.64 (0.88-3.07)	0.121	2.65 (0.98-7.10)	0.053		656	1.27 (0.83-1.95)	0.270	1.24 (0.69-2.21)	0.471
GG	278	**2.66 (1.37-5.16)**	**0.004**	**4.38 (1.52-12.65)**	**0.006**		277	**1.75 (1.09-2.81)**	**0.020**	1.63 (0.85-3.12)	0.144
rs2693											
GG	656	1 (ref)		1 (ref)			651	1 (ref)		1 (ref)	
AG	532	1.08 (0.67-1.74)	0.754	0.99 (0.50-1.95)	0.975		529	0.90 (0.64-1.28)	0.568	1.05 (0.65-1.71)	0.837
AA	108	**2.35 (1.22-4.51)**	**0.011**	**3.19 (1.01-10.04)**	**0.047**		107	1.53 (0.89-2.63)	0.124	1.93 (0.83-4.46)	0.126
rs698761											
GG	588	1 (ref)		1 (ref)			583	1 (ref)		1 (ref)	
AG	540	0.99 (0.60-1.62)	0.958	1.63 (0.78-3.39)	0.192		536	0.93 (0.64-1.34)	0.683	0.99 (0.61-1.61)	0.977
AA	168	**1.92 (1.07-3.44)**	**0.028**	**3.48 (1.45-8.33)**	**0.005**		168	**1.64 (1.05-2.58)**	**0.030**	0.90 (0.43-1.88)	0.777
rs8602											
CC	656	1 (ref)		1 (ref)			652	1 (ref)		1 (ref)	
AC	518	1.38 (0.85-2.23)	0.189	1.45 (0.73-2.88)	0.293		514	1.05 (0.74-1.49)	0.781	1.04 (0.63-1.70)	0.886
AA	123	**2.27 (1.19-4.32)**	**0.013**	**2.63 (1.04-6.65)**	**0.041**		122	1.34 (0.79-2.28)	0.278	1.27 (0.58-2.78)	0.549
rs10878441											
AA	476	1 (ref)		1 (ref)			471	1 (ref)		1 (ref)	
AC	610	1.00 (0.58-1.70)	0.986	1.09 (0.52-2.29)	0.811		606	1.07 (0.72-1.57)	0.745	1.26 (0.74-2.16)	0.396
CC	211	**2.63 (1.51-4.58)**	**0.001**	**2.46 (1.07-5.68)**	**0.035**		211	**2.11 (1.37-3.25)**	**0.001**	1.83 (0.95-3.55)	0.071
rs10318											
AA	351	1 (ref)		1 (ref)			350	1 (ref)		1 (ref)	
AG	660	0.75 (0.47-1.21)	0.242	0.47 (0.24-0.94)	0.033		654	0.77 (0.53-1.11)	0.153	**0.55 (0.32-0.94)**	**0.029**
GG	283	**0.37 (0.18-0.78)**	**0.009**	**0.32 (0.13-0.80)**	**0.015**		281	0.64 (0.39-1.03)	0.064	0.63 (0.34-1.20)	0.161
rs10075853											
AA	802	1 (ref)		1 (ref)			798	1 (ref)		1 (ref)	
AG	412	1.32 (0.82-2.14)	0.256	1.22 (0.60-2.46)	0.583		407	1.15 (0.81-1.64)	0.436	1.34 (0.82-2.17)	0.243
GG	83	**2.52 (1.30-4.91)**	**0.006**	**3.58 (1.26-10.14)**	**0.017**		83	1.34 (0.73-2.46)	0.343	1.08 (0.45-2.61)	0.857
rs8410											
GG	599	1 (ref)		1 (ref)			593	1 (ref)		1 (ref)	
AG	532	0.95 (0.58-1.56)	0.840	1.63 (0.78-3.40)	0.191		529	0.88 (0.61-1.26)	0.480	0.98 (0.60-1.59)	0.934
AA	163	**1.98 (1.11-3.54)**	**0.021**	**3.63 (1.52-8.71)**	**0.004**		163	**1.60 (1.01-2.51)**	**0.043**	0.84 (0.39-1.80)	0.647

SNP, single-nucleotide polymorphism; HR, hazard ratio; CI, confidence interval.

^#^Adjusted for age at diagnosis, education, occupation, age at menarche, number of live births, breastfeeding duration, abortion, menopause, TNM stage, tumor size, histopathologic classification, grade, lymph node, ER, PR, and HER2.

*Adjusted for age at diagnosis, number of live births, breastfeeding duration, abortion, menopause, benign breast disease, TNM stage, tumor size, histopathologic classification, grade, lymph node, ER, PR, and HER2.

### Association between 8 SNPs and breast cancer prognosis in stage II

In stage II, the median follow-up time was 67 months (0 to 143). Among the 8 SNPs identified from stage I, the SNP rs10878441 in LRRK2 gene (the duplex structure between miR-550-3p and LRRK2 was shown in Supplementary Figure 3) was significantly associated with the OS of breast cancer (AA vs CC: HR=2.21, 95% CI: 1.11-4.42, *P*=0.024) (Table 4). However, there was no association between the 8 SNPs and breast cancer DFS in multivariate analysis, and only the SNP rs10318 was significantly associated with breast cancer DFS in univariate analysis (AA vs GG: HR=0.64, 95% CI: 0.42-0.98, *P*=0.040).

**Table 4:** Association between SNP within microRNA binding sites and the prognosis of breast cancer (Stage II).

**SNP**	**Overall survival**						**Disease-free survival**				
	***N***	**HR (95% CI)**	***P***	**HR (95% CI)**	***P***^#^		***N***	**HR (95% CI)**	***P***	**HR (95% CI)**	P*
rs1053739											
AA	466	1 (ref)		1 (ref)			463	1 (ref)		1 (ref)	
AG	614	0.93 (0.65-1.33)	0.693	1.13 (0.65-1.95)	0.670		608	0.97 (0.69-1.37)	0.872	1.06 (0.64-1.74)	0.827
GG	257	**1.11 (0.73-1.70)**	**0.624**	1.38 (0.74-2.57)	0.306		255	1.21 (0.80-1.82)	0.370	1.17 (0.66-2.09)	0.596
rs2693											
GG	690	1 (ref)		1 (ref)			684	1 (ref)		1 (ref)	
AG	531	1.08 (0.78-1.51)	0.635	1.05 (0.64-1.73)	0.856		526	1.00 (0.73-1.38)	0.981	1.07 (0.68-1.69)	0.773
AA	126	**1.13 (0.67-1.91)**	**0.649**	1.48 (0.68-3.23)	0.319		126	0.79 (0.45-1.39)	0.408	1.48 (0.68-3.21)	0.321
rs698761											
GG	592	1 (ref)		1 (ref)			585	1 (ref)		1 (ref)	
AG	598	1.10 (0.78-1.53)	0.598	1.13 (0.69-1.86)	0.631		594	1.03 (0.74-1.42)	0.874	0.93 (0.59-1.47)	0.747
AA	160	**1.31 (0.73-1.91)**	**0.506**	1.12 (0.50-2.50)	0.780		160	1.15 (0.72-1.84)	0.563	1.07 (0.52-2.19)	0.863
rs8602											
CC	679	1 (ref)	0.465	1 (ref)			674	1 (ref)		1 (ref)	
AC	554	0.88 (0.63-1.23)	0.450	0.77 (0.46-1.29)	0.328		551	0.87 (0.63-1.20)	0.381	0.80 (0.50-1.28)	0.351
AA	101	**1.24 (0.71-2.14)**	**0.454**	1.61 (0.67-3.88)	0.289		98	1.34 (0.81-2.24)	0.260	1.82 (0.84-3.93)	0.127
rs10878441											
AA	498	1 (ref)		1 (ref)			493	1 (ref)		1 (ref)	
AC	614	1.35 (0.95-1.92)	0.100	1.69 (0.99-2.88)	0.055		610	**1.41 (1.00-1.98)**	**0.049**	1.57 (0.95-2.59)	0.078
CC	186	**1.27 (0.77-2.07)**	**0.349**	**2.21 (1.11-4.42)**	**0.024**		184	1.11 (0.67-1.84)	0.700	1.46 (0.74-2.87)	0.275
rs10318											
AA	376	1 (ref)		1 (ref)			374	1 (ref)		1 (ref)	
AG	643	0.74 (0.51-1.07)	0.104	1.00 (0.58-1.72)	0.984		637	**0.69 (0.49-0.98)**	**0.037**	0.78 (0.48-1.28)	0.329
GG	313	**0.70 (0.45-1.09)**	**0.115**	0.77 (0.36-1.67)	0.511		312	**0.64 (0.42-0.98)**	**0.040**	0.60 (0.31-1.15)	0.125
rs10075853											
AA	822	1 (ref)		1 (ref)			814	1 (ref)		1 (ref)	
AG	448	0.75 (0.52-1.07)	0.107	0.55 (0.31-0.98)	0.041		446	0.77 (0.54-1.08)	0.127	**0.53 (0.31-0.90)**	**0.019**
GG	78	**0.94 (0.47-1.85)**	**0.851**	1.03 (0.36-2.98)	0.950		77	1.06 (0.57-1.97)	0.854	1.61 (0.72-3.62)	0.245
rs8410											
GG	602	1 (ref)		1 (ref)			596	1 (ref)		1 (ref)	
AG	583	1.17 (0.84-1.64)	0.358	1.22 (0.75-2.00)	0.424		578	1.09 (0.79-1.51)	0.615	0.94 (0.59-1.48)	0.774
AA	157	**1.15 (0.70-1.89)**	**0.593**	1.01 (0.43-2.35)	0.981		157	1.20 (0.75-1.91)	0.458	1.02 (0.49-2.09)	0.965

SNP, single-nucleotide polymorphism; HR, hazard ratio; CI, confidence interval

^#^Adjusted for age at diagnosis, education, occupation, age at menarche, number of live births, breastfeeding duration, abortion, menopause, TNM stage, tumor size, histopathologic classification, grade, lymph node, ER, PR, and HER2.

*Adjusted for age at diagnosis, number of live births, breastfeeding duration, abortion, menopause, benign breast disease, TNM stage, tumor size, histopathologic classification, grade, lymph node, ER, PR, and HER2.

### Association between rs10878441 and breast cancer overall survival

**Figure 1:**
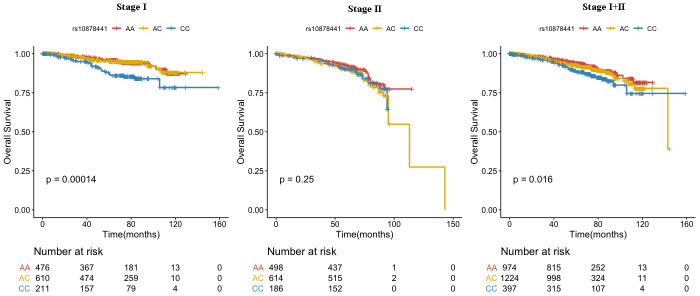
**Association between rs10878441 and the prognosis of breast cancer.**

We combined the data from stage I and stage II, compared with rs10878441 AA genotype, CC genotype was significantly connected with poor prognosis in breast cancer (HR=1.69, 95% CI: 1.18-2.42, P=0.004), which were still significantly connected with breast cancer OS when adjusted for age at diagnosis, education, occupation, age at menarche, number of live births, breastfeeding duration, abortion, menopause, TNM stage, tumor size, histopathologic classification, grade, lymph node, ER, PR, and HER2 (HR=2.19, 95% CI: 1.30-3.70, P=0.003) (Table 5 and Figure 1). Furthermore, we evaluated the association between the SNP rs10878441 and breast cancer OS stratified by clinical characteristics (Supplementary Table 3). The association was significant for grade II breast cancer patients (HR=1.64, 95% CI: 1.11-2.40, *P*=0.012; adjusted HR=1.76, 95% CI: 1.08-2.88, *P*=0.022), and was significant for lymph node-negative breast cancer patients (HR=1.78, 95% CI: 1.16-2.74, *P*=0.008; adjusted HR=2.02, 95% CI: 1.07-2.36, *P*=0.029). Specifically, this SNP was associated with breast cancer patients older than 50 years (HR=1.58, 95% CI: 1.12-2.24, *P*=0.010; adjusted HR=2.03, 95% CI: 1.21-3.42, *P*=0.008) (Supplementary Table 3).

**Table 5:** Association between rs10878441 and breast cancer overall survival.

**SNP**	***N***	**Univariate**			**Multivariate**	
		**HR (95% CI)**	***P***		**HR (95% CI)**	***P***^#^
rs10878441 (A/C) (Stage I)						
AA	476	1 (ref)			1 (ref)	
AC	610	1.00 (0.58-1.70)	0.986		1.09 (0.52-2.29)	0.811
CC	211	**2.63 (1.51-4.58)**	**0.001**		**2.46 (1.07-5.68)**	**0.035**
Additive model	1297	**1.64 (1.20-2.23)**	**0.002**		1.56 (0.99-1.45)	0.053
Dominant model	1297	1.40 (0.87-2.26)	0.170		1.38 (0.70-2.74)	0.353
Recessive model	1297	**2.63 (1.65-4.21)**	**<0.001**		**2.33 (1.16-4.68)**	**0.018**
rs10878441 (A/C) (Stage II)						
AA	498	1 (ref)			1 (ref)	
AC	614	1.35 (0.95-1.92)	0.100		1.69 (0.99-2.88)	0.055
CC	186	1.27 (0.77-2.07)	0.349		**2.21 (1.11-4.42)**	**0.024**
Additive model	1298	1.16 (0.93-1.46)	0.188		**1.51 (1.08-2.10)**	**0.015**
Dominant model	1298	1.33 (0.95-1.86)	0.100		**1.80 (1.08-2.99)**	**0.024**
Recessive model	1298	1.07 (0.69-1.67)	0.767		1.63 (0.89-2.97)	0.111
rs10878441 (A/C) (Combined)						
AA	974	1 (ref)			1 (ref)	
AC	1224	1.19 (0.89-1.60)	0.250		1.40 (0.91-2.16)	0.122
CC	397	**1.69 (1.18-2.42)**	**0.004**		**2.19 (1.30-3.70)**	**0.003**
Additive model	2595	**1.29 (1.07-1.54)**	**0.007**		**1.47 (1.13-1.92)**	**0.004**
Dominant model	2695	1.31 (0.99-1.72)	0.056		**1.57 (1.05-2.36)**	**0.028**
Recessive model	2695	**1.53 (1.12-2.10)**	**0.008**		**1.79 (1.14-2.79)**	**0.011**

SNP, single-nucleotide polymorphism; HR, hazard ratio; CI, confidence interval.

^#^Adjusted for age at diagnosis, education, occupation, age at menarche, number of live births, breastfeeding duration, abortion, menopause, TNM stage, tumor size, histopathologic classification, grade, lymph node, ER, PR, and HER2.

## DISCUSSION

Through this association study, we genotyped 192 SNPs within microRNA binding sites and found that 8 SNPs were associated with the prognosis of breast cancer. We further replicated the 8 SNPs in an independent data set, and identified that the SNP rs10878441 (C allele) in LRRK2 gene was significantly associated with poor prognosis of breast cancer. This study provided some evidence for a novel prognostic locus for breast cancer.

In this present study, two SNPs (MKNK1 rs8602, GREM1 rs10318) were previously reported in the context of cancer prognosis. MKNK1 regulates diverse biologic processes including translation, cell proliferation, and differentiation [19, 20]. Berger et al found that MKNK1 polymorphism rs8602 might serve as a predictive marker in KRAS wild-type metastatic colorectal cancer patients treated with first-line FOLFIRI and bevacizumab [21]. Neckmann et al showed that GREM1 was associated with metastasis and predicted poor prognosis in ER-negative breast cancer patients [22]. Dai et al indicated that GREM1 polymorphism rs10318 was associated with recurrence in stage II colorectal cancer patients [23]. Our study found significant association between these two SNPs and breast cancer prognosis only in stage I, while no significant difference was observed in stage II (the validation set).

The LRRK2 gene, located in human chromosome 12q12, is a member of the leucine-rich repeat kinase family and encodes a protein with multiple domains such as a leucine-rich repeat (LRR) domain, a RAS domain, a GTPase domain, a kinase domain and several protein-protein interaction domains [24]. Mutations in LRRK2 gene have been demonstrated to be associated with autosomal-dominant Parkinson’s disease [25, 26]. Studies have revealed that SNPs in LRRK2 gene have been related to Crohn’s disease [27, 28]. LRRK2 gene is involved in a variety of cellular processes including cell transformation, proliferation and tumorigenesis, and is linked to various types of cancer [29, 30]. Gu et al demonstrated that high expression of LRRK2 promoted the cell proliferation and migration of intrahepatic cholangiocarcinoma (ICC) cells, and predicted worse prognosis in ICC patients [31]. Looyenga et al indicated that MET and LRRK2 cooperated to promote efficient tumor cell growth and survival in papillary renal and thyroid carcinomas [29]. Warø et al reported that LRRK2 mutation carriers had an increased risk of non-skin cancer [32].

Our findings suggest that the C allele of LRRK2 has poor prognosis in breast cancer. LRRK2 expression may be regulated in a variety of ways, while the association between the SNP rs10878441 and the prognosis of breast cancer might be caused by differential microRNA regulation. SNP rs10878441 (A/C) is located within the miR-550-3p binding site, and it is likely to affect the miR-550-3p/LRRK2 interaction. As shown in Supplementary Figure 3, the C allele cannot be targeted by miR-550-3p, leading to an increase expression of LRRK2 protein, thereby altering the prognosis of breast cancer. The expression analysis of TCGA data in Supplementary Figure 4 showed that CC genotype increased the expression of LRRK2 in 1058 breast cancer patients. The definite underlying mechanism for the association with the prognosis of breast cancer remains unknown. Lin et al identified a LINK-A lncRNA that mediated HIF1α phosphorylation at Ser797 by LRRK2, resulting in the activation of normoxic HIF1α signaling and promoting glycolysis reprogramming, tumorigenesis and progression in triple-negative breast cancer [33]. Jiang et al revealed that downregulated LRRK2 gene expression inhibited proliferation and migration while promoting the apoptosis of thyroid cancer cells by inhibiting activation of the JNK signaling pathway [34].

Although we conducted a large systematic two-stage cohort study to evaluate mircoRNA target SNPs and breast cancer prognosis, our study has several limitations. First, we only selected high frequency SNPs with MAF ≥ 0.05, inevitably miss low frequency SNPs that have an impact on breast cancer prognosis. Second, Type 1 error of multiple testing was not corrected in this study, although our design with large sample size and replication set can ensure a high repeatability of our findings. Third, due to the good prognosis of breast cancer patients, the number of deaths and tumor progression were small, and further follow-up will be required to confirm the reliability of the results. In addition, it would be more plausible if we had the data of the expression level of miRNAs and their target genes in clinical samples, further studies are warranted to evaluate the meaning of SNPs on miRNA binding sites in breast cancer biology.

In conclusion, the LRRK2 rs10878441 CC genotype is associated with poor prognosis of breast cancer in a Chinese population, suggesting that it could be a potential prognostic biomarker for breast cancer. Further studies to elucidate the underling mechanism for this association are warranted.

## MATERIALS AND METHODS

### Study subjects

We performed a two-stage cohort study including 2647 breast cancer patients, with 1297 and 1350 breast cancer patients in stage I and stage II, respectively. All patients were newly diagnosed and histologically confirmed for breast cancer at Tianjin Medical University Cancer Hospital (TJMUCH) from January 2006 to December 2012. The two stages were defined according to the time of sample enrollment. In stage I, we selected 1297 patients from January 2006 to December 2008 for SNP screening. In stage II, to validate the findings from stage I, the validation set of 1350 patients from January 2009 to December 2012 were genotyped. The detailed description of Tianjin Cohort of Breast Cancer Cases (TBCCC) can be obtained in our previous study [35]. Demographic and epidemiological data were obtained from face-to-face questionnaires by trained personnel. Clinical data and pathology reports were taken from medical records. All patients were followed up by telephone annually. In addition, we further confirmed the accuracy of self-reported information through Hospital information system (HIS) at TJMUCH and death registration system. The study was approved by the Ethics Committee of Tianjin Medical University Cancer Institute and Hospital, and all patients participated in the study signed written informed consent.

### SNP selection

The “Patrocles” database (http://www.patrocles.org/) was used to select genome-wide microRNA target SNPs. Of all the 5035 SNPs within microRNA binding site provided by the database, 1742 SNPs had been confirmed. At the same time, SNPs for inclusion conformed with the following criteria: (1) SNPs located at the binding site of microRNA-seed region, and the seed region was defined according to the “7-mirs” criteria [36]. (2) SNPs have Chinese population frequency data (http://www.ncbi.nlm.nih.gov/snp/), and SNPs have three genotypes with minor genotype frequency (MAF) ≥0.05. Finally, 192 microRNA target SNPs were included in our study, the detailed information of these SNPs were shown in Supplementary Table 1.

### SNP genotyping

We collected 10 ml ETDA-anticoagulated venous blood, and separated the plasma and white blood cell layer, and stored the white blood cells in a cryotube at -80° C Celsius refrigerator for DNA extraction. Genomic DNA was extracted using QIAGEN DNA Extraction Kit (QIAGEN Inc.) [37]. The Illumina Golden Gate SNP Genotyping Arrays was used to genotype 192 SNPs in stage I. The TaqMan platform was taken to genotype 8 SNPs associated with breast cancer prognosis in stage II. We used a 5-μl reaction mixture system with 20 ng of genomic DNA, 2.5 μl of 2×TaqMan Genotyping Master Mix, 0.1 μl of 40×probe and 1.9μl of double distilled water. The PCR reaction conditions were 95° C for 10 minutes, followed by 50 cycles of 92° C for 30 seconds, and 60° C for 1 minutes. We amplified using the 384-well reaction plates and performed genotype analysis using SDS 2.4 software (Applied Biosystems, Foster City, CA, USA). In order to ensure the accuracy and reliability of the experimental results, approximately 5% of the samples were randomly selected for retesting.

### Follow-up of breast cancer

Followed-up information included follow-up date, vital status (alive, dead, and lost to follow-up), tumor progression (recurrence, metastasis), and treatment after tumor progression. Overall survival (OS) was defined as the time from the date of breast cancer diagnosis to the date of death from any cause. Disease-free survival (DFS) was calculated as the time from breast cancer diagnosis to the date of tumor progression (recurrence, metastasis or death). If patients were lost to follow-up, the follow-up date was calculated based on the date of the last visit. Follow-up of this study was completed on December 31, 2017.

### Statistical analysis

Patients’ characteristics such as demographic, epidemiological and clinicopathological are represented by *n* (%). The Kaplan-Meier method was used to calculate survival estimates, and log-rank test was used to compare the survival differences of these SNPs. To determine potential prognostic risk factors, univariate Cox regression was used to evaluate the relationship between demographic, epidemiological and clinicopathological characteristics and breast cancer prognosis, presented as hazard ratios (HRs) and 95% confidence intervals (CIs). Cox regression was used to appraise the association between SNPs and breast cancer OS, with and without adjustments for age at diagnosis, education, occupation, age at menarche, number of live births, breastfeeding duration, abortion, menopause, TNM stage, tumor size, histopathologic classification, grade, lymph node, estrogen receptor (ER), progestogen receptor (PR), and HER2. Similarly, Cox regression was used to assess the relationship between SNPs and breast cancer DFS, with and without adjustments for age at diagnosis, number of live births, breastfeeding duration, abortion, menopause, benign breast disease (BBD), TNM stage, tumor size, histopathologic classification, grade, lymph node, ER, PR, and HER2. We further analyzed the relationship between the SNP rs10878441 and breast cancer OS stratified by clinical characteristics. All statistical tests were two-sided and *P*<0.05 was considered statistically significant. All statistical analysis was performed using SPSS 20.0 software (SPSS Inc. Chicago, IL, USA) and R version 3.4.3.

## Supplementary Material

Supplementary Figures

Supplementary Table 1

Supplementary Table 2

Supplementary Table 3
