# Supplementary material for: SNPs within microRNA binding sites and the prognosis of breast cancer
Source: Aging (Albany NY). 2021 Feb 26;13(5):7465–80. doi: 10.18632/aging.202612 (PMC7993692; doi:10.18632/aging.202612)
Supplement: Supplementary Figures [file aging-13-202612-s001.pdf]

SUPPLEMENTARY FIGURES

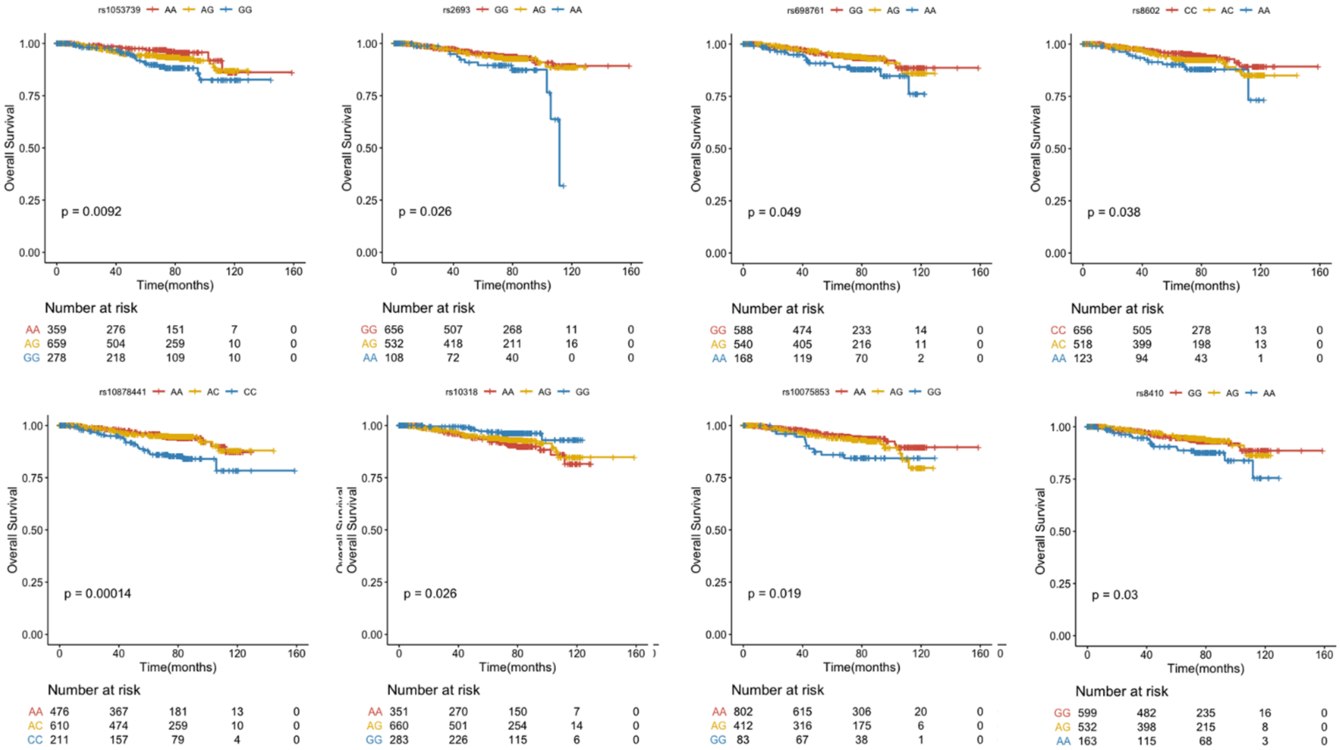

Supplementary Figure 1. Association between 8 SNPs and breast cancer OS in stage I.

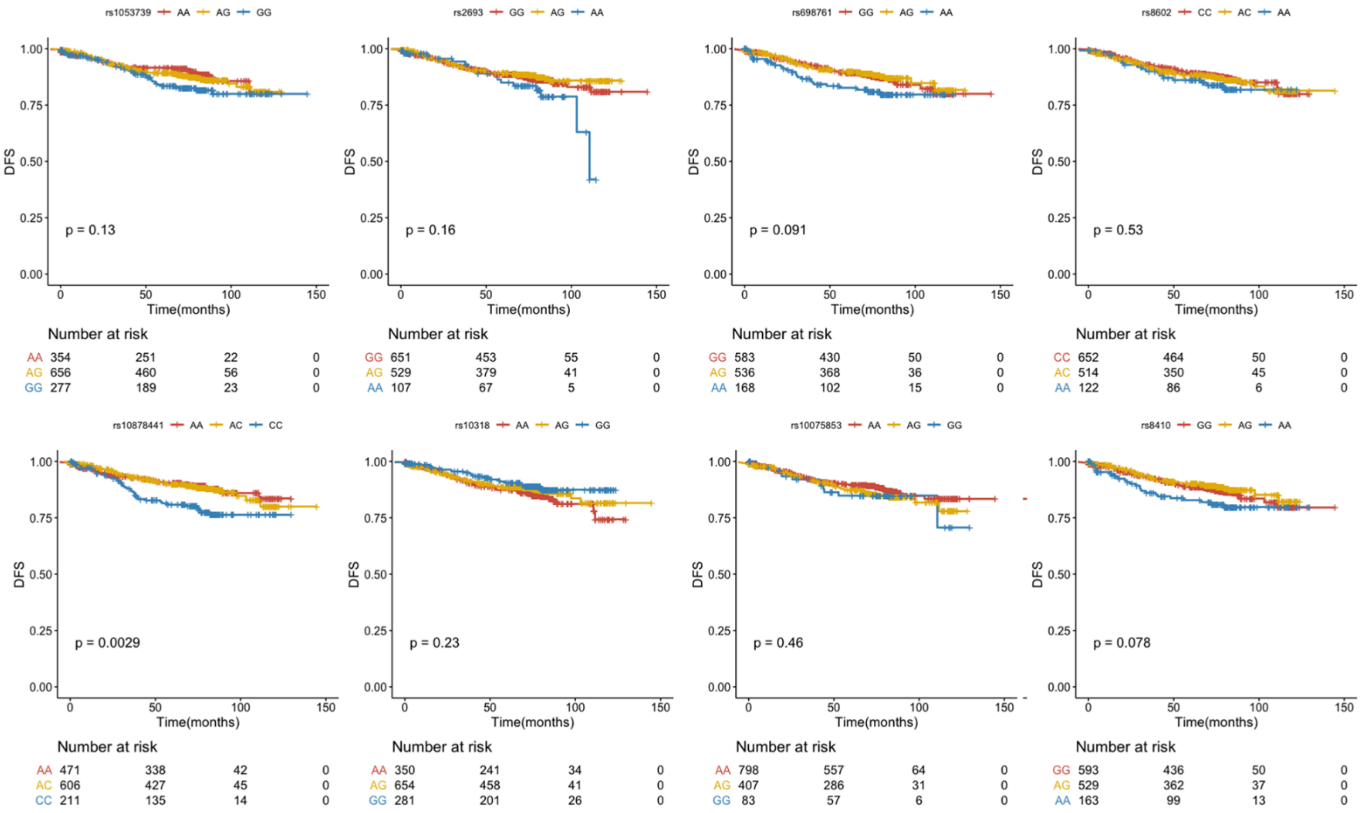

Supplementary Figure 2. Association between 8 SNPs and breast cancer DFS in stage I.

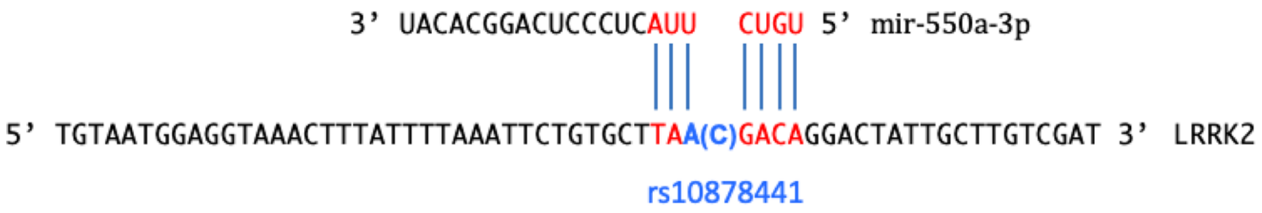

Supplementary Figure 3. The duplex structure of hsa-miR-550*and the 3’UTR of LRRK2 gene.

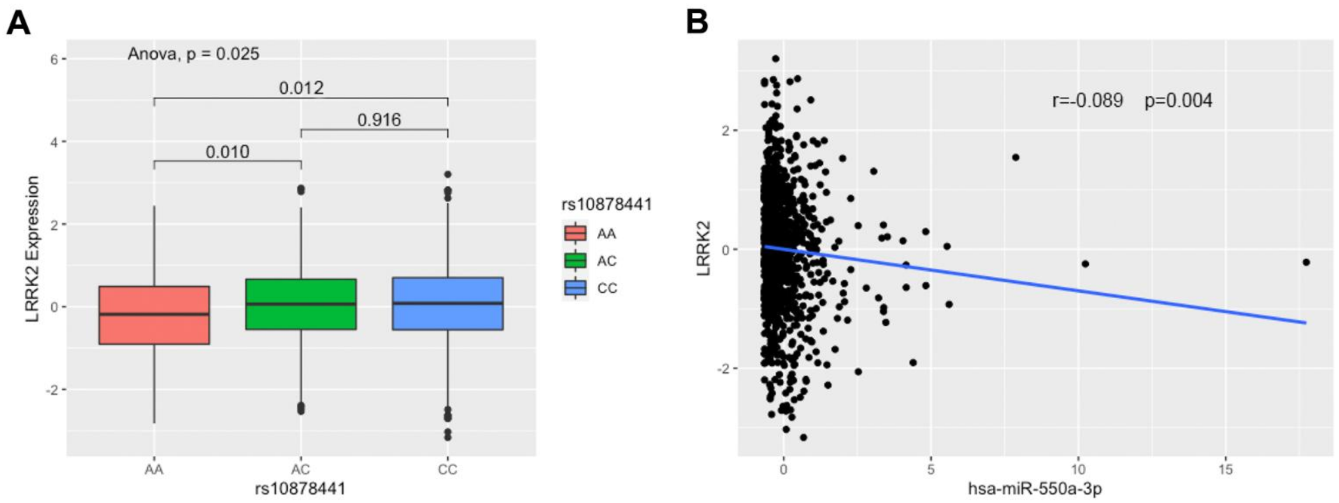

Supplementary Figure 4. Expression analysis of TCGA data. (**A**) The relationship between rs10878441 and LRRK2 gene expression. (**B**) The relationship between the expression of has-miR-550a-3p and the expression of LRRK2.
