# Supplementary material for: SNPs within microRNA binding sites and the prognosis of breast cancer
Source: Aging (Albany NY). 2021 Feb 26;13(5):7465–80. doi: 10.18632/aging.202612 (PMC7993692; doi:10.18632/aging.202612)
Supplement: Supplementary Table 1 [file aging-13-202612-s002.docx]

**Supplementary Table 1. 192 microRNA binding site SNPs identified from “Patrocles” database.**

| **rs number** | **SNP** | **Gene** | **Target sequence** | **Micro-RNA** |
| --- | --- | --- | --- | --- |
| [**rs10075853**](http://www.ncbi.nlm.nih.gov/SNP/snp_ref.cgi?rs=rs10075853) | C/T | ST8SIA4(CMP-N-acetylneuraminate-poly-alpha-2,8-sialyltransferase) | **AAGCACA** | [hsa-miR-218](http://microrna.sanger.ac.uk/cgi-bin/sequences/mirna_entry.pl?acc=MIMAT0000275) |
| [**rs10196**](http://www.ncbi.nlm.nih.gov/SNP/snp_ref.cgi?rs=rs10196) | G/A | NDRG2(Protein NDRG2) | **TGGTGTT** | [hsa-miR-21*](http://microrna.sanger.ac.uk/cgi-bin/sequences/mirna_entry.pl?acc=MIMAT0004494) |
| [**rs10205020**](http://www.ncbi.nlm.nih.gov/SNP/snp_ref.cgi?rs=rs10205020) | A/G | PPP1R1C(Protein phosphatase 1 regulatory subunit 1C) | **CTTTCAA** | [hsa-miR-488](http://microrna.sanger.ac.uk/cgi-bin/sequences/mirna_entry.pl?acc=MIMAT0004763) |
| [**rs10270308**](http://www.ncbi.nlm.nih.gov/SNP/snp_ref.cgi?rs=rs10270308) | T/C | CDNA FLJ44366 fis, clone TRACH3008629, similar to Cadherin related tumor suppressor | **TGGAGAA** | [hsa-miR-515-5p](http://microrna.sanger.ac.uk/cgi-bin/sequences/mirna_entry.pl?acc=MIMAT0002826) |
| [**rs10305516**](http://www.ncbi.nlm.nih.gov/SNP/snp_ref.cgi?rs=rs10305516) | C/G | GLP1R(Glucagon-like peptide 1 receptor precursor) | **AGGGCAG** | [hsa-miR-18a*](http://microrna.sanger.ac.uk/cgi-bin/sequences/mirna_entry.pl?acc=MIMAT0002891) |
| [**rs10318**](http://www.ncbi.nlm.nih.gov/SNP/snp_ref.cgi?rs=rs10318) | C/T | GREM1(Gremlin-1 precursor) | **CTATTAA** | [hsa-miR-633](http://microrna.sanger.ac.uk/cgi-bin/sequences/mirna_entry.pl?acc=MIMAT0003303) |
| [**rs1043291**](http://www.ncbi.nlm.nih.gov/SNP/snp_ref.cgi?rs=rs1043291) | G/A | C7orf27(HEAT repeat domain-containing protein C7orf27 precursor) | **GAGAAGA** | [hsa-miR-942](http://microrna.sanger.ac.uk/cgi-bin/sequences/mirna_entry.pl?acc=MIMAT0004985) |
| [**rs10437**](http://www.ncbi.nlm.nih.gov/SNP/snp_ref.cgi?rs=rs10437) | A/G | GLS(Glutaminase kidney isoform, mitochondrial precursor) | **ACCAAAG** | [hsa-miR-9](http://microrna.sanger.ac.uk/cgi-bin/sequences/mirna_entry.pl?acc=MIMAT0000441) |
| [**rs1043881**](http://www.ncbi.nlm.nih.gov/SNP/snp_ref.cgi?rs=rs1043881) | A/G | BCAT1(Branched-chain-amino-acid aminotransferase, cytosolic) | **CTGGCAA** | [hsa-miR-1202](http://microrna.sanger.ac.uk/cgi-bin/sequences/mirna_entry.pl?acc=MIMAT0005865) |
| [**rs1043915**](http://www.ncbi.nlm.nih.gov/SNP/snp_ref.cgi?rs=rs1043915) | A/T | opposite strand transcription unit to STAG3 | **ACAATCA** | [hsa-miR-219-5p](http://microrna.sanger.ac.uk/cgi-bin/sequences/mirna_entry.pl?acc=MIMAT0000276) |
| [**rs1044123**](http://www.ncbi.nlm.nih.gov/SNP/snp_ref.cgi?rs=rs1044123) | C/T | NOTCH3(Neurogenic locus notch homolog protein 3 precursor) | **AGCCTCA** | [hsa-miR-485-5p](http://microrna.sanger.ac.uk/cgi-bin/sequences/mirna_entry.pl?acc=MIMAT0002175) |
| [**rs1044145**](http://www.ncbi.nlm.nih.gov/SNP/snp_ref.cgi?rs=rs1044145) | G/A | YOD1(Ubiquitin thioesterase OTU1) | **TGCACTG** | hsa-miR-148 |
| [**rs1044158**](http://www.ncbi.nlm.nih.gov/SNP/snp_ref.cgi?rs=rs1044158) | T/C | TMEM150(Transmembrane protein 150 precursor) | **TGTACCA** | [hsa-miR-150*](http://microrna.sanger.ac.uk/cgi-bin/sequences/mirna_entry.pl?acc=MIMAT0004610) |
| [**rs1044268**](http://www.ncbi.nlm.nih.gov/SNP/snp_ref.cgi?rs=rs1044268) | G/A | NRP1(Neuropilin-1 precursor) | **CAGCGAA** | [hsa-miR-639](http://microrna.sanger.ac.uk/cgi-bin/sequences/mirna_entry.pl?acc=MIMAT0003309) |
| [**rs1045004**](http://www.ncbi.nlm.nih.gov/SNP/snp_ref.cgi?rs=rs1045004) | G/A | FBXO34(F-box only protein 34) | **CCCTGTG** | [hsa-miR-220c](http://microrna.sanger.ac.uk/cgi-bin/sequences/mirna_entry.pl?acc=MIMAT0004915) |
| [**rs1045832**](http://www.ncbi.nlm.nih.gov/SNP/snp_ref.cgi?rs=rs1045832) | C/T | AMMECR1L(AMMECR1-like protein) | **GCCAGGA** | [hsa-miR-221*](http://microrna.sanger.ac.uk/cgi-bin/sequences/mirna_entry.pl?acc=MIMAT0004568) |
| [**rs1047499**](http://www.ncbi.nlm.nih.gov/SNP/snp_ref.cgi?rs=rs1047499) | T/C | SPTBN1(Spectrin beta chain, brain 1) | **GGTGTGT** | [hsa-miR-329](http://microrna.sanger.ac.uk/cgi-bin/sequences/mirna_entry.pl?acc=MIMAT0001629) |
| [**rs1048906**](http://www.ncbi.nlm.nih.gov/SNP/snp_ref.cgi?rs=rs1048906) | A/G | IKK interacting protein isoform 3.1 | **AGTGACA** | [hsa-miR-668](http://microrna.sanger.ac.uk/cgi-bin/sequences/mirna_entry.pl?acc=MIMAT0003881) |
| [**rs1049269**](http://www.ncbi.nlm.nih.gov/SNP/snp_ref.cgi?rs=rs1049269) | C/T | SPOCK2(Testican-2 precursor) | **CCACTGA** | [hsa-miR-194*](http://microrna.sanger.ac.uk/cgi-bin/sequences/mirna_entry.pl?acc=MIMAT0004671) |
| [**rs1049684**](http://www.ncbi.nlm.nih.gov/SNP/snp_ref.cgi?rs=rs1049684) | T/C | CABYR(Calcium-binding tyrosine phosphorylation-regulated protein) | **CTGGACA** | [hsa-miR-198](http://microrna.sanger.ac.uk/cgi-bin/sequences/mirna_entry.pl?acc=MIMAT0000228) |
| [**rs1050488**](http://www.ncbi.nlm.nih.gov/SNP/snp_ref.cgi?rs=rs1050488) | C/T | NSMAF(Protein FAN) | **GTTGCCA** | [hsa-miR-196a*](http://microrna.sanger.ac.uk/cgi-bin/sequences/mirna_entry.pl?acc=MIMAT0004562) |
| [**rs10510454**](http://www.ncbi.nlm.nih.gov/SNP/snp_ref.cgi?rs=rs10510454) | G/A | DAZL(Deleted in azoospermia-like) | **AGTCCAA** | [hsa-miR-1269](http://microrna.sanger.ac.uk/cgi-bin/sequences/mirna_entry.pl?acc=MIMAT0005923) |
| [**rs1051148**](http://www.ncbi.nlm.nih.gov/SNP/snp_ref.cgi?rs=rs1051148) | G/T | SLC35A1(CMP-sialic acid transporter) | **TTTGCTG** | [hsa-miR-545](http://microrna.sanger.ac.uk/cgi-bin/sequences/mirna_entry.pl?acc=MIMAT0003165) |
| [**rs1051424**](http://www.ncbi.nlm.nih.gov/SNP/snp_ref.cgi?rs=rs1051424) | A/G | RPS6KB1(Ribosomal protein S6 kinase beta-1) | **CAAAAAA** | [hsa-miR-129-5p](http://microrna.sanger.ac.uk/cgi-bin/sequences/mirna_entry.pl?acc=MIMAT0000242) |
| [**rs1051434**](http://www.ncbi.nlm.nih.gov/SNP/snp_ref.cgi?rs=rs1051434) | G/A | MPHOSPH9(M-phase phosphoprotein 9) | **GGCCACA** | [hsa-miR-663b](http://microrna.sanger.ac.uk/cgi-bin/sequences/mirna_entry.pl?acc=MIMAT0005867) |
| [**rs1053739**](http://www.ncbi.nlm.nih.gov/SNP/snp_ref.cgi?rs=rs1053739) | G/A | NMT1(Glycylpeptide N-tetradecanoyltransferase 1) | **CTGGCAA** | [hsa-miR-1202](http://microrna.sanger.ac.uk/cgi-bin/sequences/mirna_entry.pl?acc=MIMAT0005865) |
| [**rs1056471**](http://www.ncbi.nlm.nih.gov/SNP/snp_ref.cgi?rs=rs1056471) | C/G | HADHB(Trifunctional enzyme subunit beta, mitochondrial precursor (TP-beta)) | **ATACTTT** | [hsa-miR-548l](http://microrna.sanger.ac.uk/cgi-bin/sequences/mirna_entry.pl?acc=MIMAT0005889) |
| [**rs1056796**](http://www.ncbi.nlm.nih.gov/SNP/snp_ref.cgi?rs=rs1056796) | T/G | MLANA(Melanoma antigen recognized by T-cells 1) | **ACAGGTT** | [hsa-miR-649](http://microrna.sanger.ac.uk/cgi-bin/sequences/mirna_entry.pl?acc=MIMAT0003319) |
| [**rs1058267**](http://www.ncbi.nlm.nih.gov/SNP/snp_ref.cgi?rs=rs1058267) | G/A | PCDH10(Protocadherin-10 precursor) | **ATGTGAA** | hsa-miR-23 |
| [**rs1058747**](http://www.ncbi.nlm.nih.gov/SNP/snp_ref.cgi?rs=rs1058747) | G/A | PHLPPL(PH domain leucine-rich repeat protein phosphatase-like) | **AAGCACT** | [hsa-miR-520f](http://microrna.sanger.ac.uk/cgi-bin/sequences/mirna_entry.pl?acc=MIMAT0002830) |
| [**rs1059111**](http://www.ncbi.nlm.nih.gov/SNP/snp_ref.cgi?rs=rs1059111) | T/A | NEFL(Neurofilament light polypeptide (NF-L)) | **CATGGTC** | [hsa-miR-591](http://microrna.sanger.ac.uk/cgi-bin/sequences/mirna_entry.pl?acc=MIMAT0003259) |
| [**rs1059310**](http://www.ncbi.nlm.nih.gov/SNP/snp_ref.cgi?rs=rs1059310) | A/G | SOS1(Son of sevenless homolog 1) | **ATGGAAA** | [hsa-miR-587](http://microrna.sanger.ac.uk/cgi-bin/sequences/mirna_entry.pl?acc=MIMAT0003253) |
| [**rs1059829**](http://www.ncbi.nlm.nih.gov/SNP/snp_ref.cgi?rs=rs1059829) | T/C | SPARC(SPARC precursor) | **CTTTCAA** | [hsa-miR-488](http://microrna.sanger.ac.uk/cgi-bin/sequences/mirna_entry.pl?acc=MIMAT0004763) |
| [**rs1062679**](http://www.ncbi.nlm.nih.gov/SNP/snp_ref.cgi?rs=rs1062679) | G/C | SLC4A4(Electrogenic sodium bicarbonate cotransporter 1) | **AGTTTTG** | [hsa-miR-1323](http://microrna.sanger.ac.uk/cgi-bin/sequences/mirna_entry.pl?acc=MIMAT0005795) |
| [**rs1065518**](http://www.ncbi.nlm.nih.gov/SNP/snp_ref.cgi?rs=rs1065518) | A/T | RALB(Ras-related protein Ral-B precursor) | **TCAGTGG** | [hsa-miR-181a-2*](http://microrna.sanger.ac.uk/cgi-bin/sequences/mirna_entry.pl?acc=MIMAT0004558) |
| [**rs10878441**](http://www.ncbi.nlm.nih.gov/SNP/snp_ref.cgi?rs=rs10878441) | A/C | LRRK2(Leucine-rich repeat serine/threonine-protein kinase 2) | **TAAGACA** | [hsa-miR-550*](http://microrna.sanger.ac.uk/cgi-bin/sequences/mirna_entry.pl?acc=MIMAT0003257) |
| [**rs10888486**](http://www.ncbi.nlm.nih.gov/SNP/snp_ref.cgi?rs=rs10888486) | G/A | CRNN(cornulin) | **GACTCCA** | [hsa-miR-1289](http://microrna.sanger.ac.uk/cgi-bin/sequences/mirna_entry.pl?acc=MIMAT0005879) |
| [**rs10980234**](http://www.ncbi.nlm.nih.gov/SNP/snp_ref.cgi?rs=rs10980234) | A/G | AKAP2(Paralemmin-2) | **AGTGTTA** | [hsa-miR-141](http://microrna.sanger.ac.uk/cgi-bin/sequences/mirna_entry.pl?acc=MIMAT0000432) |
| [**rs11108452**](http://www.ncbi.nlm.nih.gov/SNP/snp_ref.cgi?rs=rs11108452) | G/A | PCTK2(Serine/threonine-protein kinase PCTAIRE-2) | **AACTGTA** | [hsa-miR-582-5p](http://microrna.sanger.ac.uk/cgi-bin/sequences/mirna_entry.pl?acc=MIMAT0003247) |
| [**rs11173459**](http://www.ncbi.nlm.nih.gov/SNP/snp_ref.cgi?rs=rs11173459) | A/G | SLC2A13(Proton myo-inositol cotransporter) | **GTGACTT** | [hsa-miR-224](http://microrna.sanger.ac.uk/cgi-bin/sequences/mirna_entry.pl?acc=MIMAT0000281) |
| [**rs1130741**](http://www.ncbi.nlm.nih.gov/SNP/snp_ref.cgi?rs=rs1130741) | A/G | MPI(Mannose-6-phosphate isomerase) | **ACAGTGA** | [hsa-miR-1208](http://microrna.sanger.ac.uk/cgi-bin/sequences/mirna_entry.pl?acc=MIMAT0005873) |
| [**rs1131382**](http://www.ncbi.nlm.nih.gov/SNP/snp_ref.cgi?rs=rs1131382) | A/G | SLC23A2(Solute carrier family 23 member 2) | **ACTGTAG** | [hsa-miR-139-5p](http://microrna.sanger.ac.uk/cgi-bin/sequences/mirna_entry.pl?acc=MIMAT0000250) |
| [**rs11538239**](http://www.ncbi.nlm.nih.gov/SNP/snp_ref.cgi?rs=rs11538239) | T/C | ZFAT1(Zinc finger protein 406) | **AGCAATA** | [hsa-miR-137](http://microrna.sanger.ac.uk/cgi-bin/sequences/mirna_entry.pl?acc=MIMAT0000429) |
| [**rs11724758**](http://www.ncbi.nlm.nih.gov/SNP/snp_ref.cgi?rs=rs11724758) | T/C | FABP2(Fatty acid-binding protein, intestinal) | **GACTGTT** | [hsa-miR-212](http://microrna.sanger.ac.uk/cgi-bin/sequences/mirna_entry.pl?acc=MIMAT0000269) |
| [**rs1180342**](http://www.ncbi.nlm.nih.gov/SNP/snp_ref.cgi?rs=rs1180342) | T/G | BMP8A(Bone morphogenetic protein 8A precursor) | **TGTCCCA** | [hsa-miR-1302](http://microrna.sanger.ac.uk/cgi-bin/sequences/mirna_entry.pl?acc=MIMAT0005890) |
| [**rs11844549**](http://www.ncbi.nlm.nih.gov/SNP/snp_ref.cgi?rs=rs11844549) | A/G | MDGA2(MAM domain-containing glycosylphosphatidylinositol anchor protein 2 precursor) | **AAGCACT** | [hsa-miR-520f](http://microrna.sanger.ac.uk/cgi-bin/sequences/mirna_entry.pl?acc=MIMAT0002830) |
| [**rs11912802**](http://www.ncbi.nlm.nih.gov/SNP/snp_ref.cgi?rs=rs11912802) | C/T | SYN3(Synapsin-3) | **TCAGCAA** | [hsa-miR-628-5p](http://microrna.sanger.ac.uk/cgi-bin/sequences/mirna_entry.pl?acc=MIMAT0004809) |
| [**rs11920311**](http://www.ncbi.nlm.nih.gov/SNP/snp_ref.cgi?rs=rs11920311) | A/G | PLXND1(Plexin-D1 precursor) | **GGTGGTG** | [hsa-miR-220b](http://microrna.sanger.ac.uk/cgi-bin/sequences/mirna_entry.pl?acc=MIMAT0004908) |
| [**rs1194**](http://www.ncbi.nlm.nih.gov/SNP/snp_ref.cgi?rs=rs1194) | G/A | ZNF76(Zinc finger protein 76) | **GCTGGAG** | [hsa-miR-766](http://microrna.sanger.ac.uk/cgi-bin/sequences/mirna_entry.pl?acc=MIMAT0003888) |
| [**rs12107172**](http://www.ncbi.nlm.nih.gov/SNP/snp_ref.cgi?rs=rs12107172) | A/G | ZIC1(Zinc finger protein ZIC 1) | **TAATAAT** | [hsa-miR-126*](http://microrna.sanger.ac.uk/cgi-bin/sequences/mirna_entry.pl?acc=MIMAT0000444) |
| [**rs12191285**](http://www.ncbi.nlm.nih.gov/SNP/snp_ref.cgi?rs=rs12191285) | T/C | MDGA1(MAM domain-containing glycosylphosphatidylinositol anchor protein 1 precursor) | **GACCCTA** | [hsa-miR-1182](http://microrna.sanger.ac.uk/cgi-bin/sequences/mirna_entry.pl?acc=MIMAT0005827) |
| [**rs1235**](http://www.ncbi.nlm.nih.gov/SNP/snp_ref.cgi?rs=rs1235) | C/A | MDGA2(MAM domain-containing glycosylphosphatidylinositol anchor protein 2 precursor) | **CTGATTA** | [hsa-miR-34a*](http://microrna.sanger.ac.uk/cgi-bin/sequences/mirna_entry.pl?acc=MIMAT0004557) |
| [**rs12636077**](http://www.ncbi.nlm.nih.gov/SNP/snp_ref.cgi?rs=rs12636077) | G/A | ACVR2B(Activin receptor type-2B precursor) | **AGGCCGA** | [hsa-miR-1234](http://microrna.sanger.ac.uk/cgi-bin/sequences/mirna_entry.pl?acc=MIMAT0005589) |
| [**rs12677519**](http://www.ncbi.nlm.nih.gov/SNP/snp_ref.cgi?rs=rs12677519) | C/T | RBM35A(RNA-binding protein 35A) | **CATGAAC** | [hsa-miR-1206](http://microrna.sanger.ac.uk/cgi-bin/sequences/mirna_entry.pl?acc=MIMAT0005870) |
| [**rs12705977**](http://www.ncbi.nlm.nih.gov/SNP/snp_ref.cgi?rs=rs12705977) | T/G | FOXP2(Forkhead box protein P2) | **TGGACTG** | [hsa-miR-455-3p](http://microrna.sanger.ac.uk/cgi-bin/sequences/mirna_entry.pl?acc=MIMAT0004784) |
| [**rs12913**](http://www.ncbi.nlm.nih.gov/SNP/snp_ref.cgi?rs=rs12913) | T/C | NKD1(naked cuticle homolog 1) | **CTTCTCA** | [hsa-miR-1300](http://microrna.sanger.ac.uk/cgi-bin/sequences/mirna_entry.pl?acc=MIMAT0005888) |
| [**rs13242090**](http://www.ncbi.nlm.nih.gov/SNP/snp_ref.cgi?rs=rs13242090) | T/C | CASD1(CAS1 domain containing 1) | **TACTTTT** | [hsa-miR-548n](http://microrna.sanger.ac.uk/cgi-bin/sequences/mirna_entry.pl?acc=MIMAT0005916) |
| [**rs1325774**](http://www.ncbi.nlm.nih.gov/SNP/snp_ref.cgi?rs=rs1325774) | T/G | CLDN10(Claudin-10) | **CTGGCAA** | [hsa-miR-1202](http://microrna.sanger.ac.uk/cgi-bin/sequences/mirna_entry.pl?acc=MIMAT0005865) |
| [**rs13422**](http://www.ncbi.nlm.nih.gov/SNP/snp_ref.cgi?rs=rs13422) | C/A | PMP22(Peripheral myelin protein 22) | **AAACCAG** | [hsa-miR-29b-1*](http://microrna.sanger.ac.uk/cgi-bin/sequences/mirna_entry.pl?acc=MIMAT0004514) |
| [**rs13429321**](http://www.ncbi.nlm.nih.gov/SNP/snp_ref.cgi?rs=rs13429321) | T/A | GALNT3(Polypeptide N-acetylgalactosaminyltransferase 3) | **GTGATGT** | [hsa-miR-499-3p](http://microrna.sanger.ac.uk/cgi-bin/sequences/mirna_entry.pl?acc=MIMAT0004772) |
| [**rs13734**](http://www.ncbi.nlm.nih.gov/SNP/snp_ref.cgi?rs=rs13734) | C/T | RRBP1(Ribosome-binding protein 1) | **TCCAGGC** | [hsa-miR-1254](http://microrna.sanger.ac.uk/cgi-bin/sequences/mirna_entry.pl?acc=MIMAT0005905) |
| [**rs1395119**](http://www.ncbi.nlm.nih.gov/SNP/snp_ref.cgi?rs=rs1395119) | T/A | SIM1(Single-minded homolog 1) | **ATACACA** | [hsa-miR-223*](http://microrna.sanger.ac.uk/cgi-bin/sequences/mirna_entry.pl?acc=MIMAT0004570) |
| [**rs14240**](http://www.ncbi.nlm.nih.gov/SNP/snp_ref.cgi?rs=rs14240) | T/C | SLC18A2(Synaptic vesicular amine transporter) | **ATTTGAA** | [hsa-miR-607](http://microrna.sanger.ac.uk/cgi-bin/sequences/mirna_entry.pl?acc=MIMAT0003275) |
| [**rs1509617**](http://www.ncbi.nlm.nih.gov/SNP/snp_ref.cgi?rs=rs1509617) | C/G | PDLIM5(PDZ and LIM domain protein 5) | **AGCTCCC** | [hsa-miR-920](http://microrna.sanger.ac.uk/cgi-bin/sequences/mirna_entry.pl?acc=MIMAT0004970) |
| [**rs1595066**](http://www.ncbi.nlm.nih.gov/SNP/snp_ref.cgi?rs=rs1595066) | G/A | ERBB4(Receptor tyrosine-protein kinase erbB-4 precursor) | **GTACTTT** | [hsa-miR-548k](http://microrna.sanger.ac.uk/cgi-bin/sequences/mirna_entry.pl?acc=MIMAT0005882) |
| [**rs1599796**](http://www.ncbi.nlm.nih.gov/SNP/snp_ref.cgi?rs=rs1599796) | C/T | CD80(T-lymphocyte activation antigen CD80 precursor) | **AGATGAA** | [hsa-miR-452*](http://microrna.sanger.ac.uk/cgi-bin/sequences/mirna_entry.pl?acc=MIMAT0001636) |
| [**rs16845990**](http://www.ncbi.nlm.nih.gov/SNP/snp_ref.cgi?rs=rs16845990) | A/G | ERBB4(Receptor tyrosine-protein kinase erbB-4 precursor) | **AGCCCCA** | [hsa-miR-185*](http://microrna.sanger.ac.uk/cgi-bin/sequences/mirna_entry.pl?acc=MIMAT0004611) |
| [**rs16974880**](http://www.ncbi.nlm.nih.gov/SNP/snp_ref.cgi?rs=rs16974880) | T/G | CRISPLD2(Cysteine-rich secretory protein LCCL domain-containing 2 precursor) | **AACTGGA** | [hsa-miR-145](http://microrna.sanger.ac.uk/cgi-bin/sequences/mirna_entry.pl?acc=MIMAT0000437) |
| [**rs17053540**](http://www.ncbi.nlm.nih.gov/SNP/snp_ref.cgi?rs=rs17053540) | C/T | EYA4(Eyes absent homolog 4) | **GAATGCT** | [hsa-miR-1179](http://microrna.sanger.ac.uk/cgi-bin/sequences/mirna_entry.pl?acc=MIMAT0005824) |
| [**rs17063228**](http://www.ncbi.nlm.nih.gov/SNP/snp_ref.cgi?rs=rs17063228) | A/T | SLC2A12(Solute carrier family 2, facilitated glucose transporter member 12) | **ACTGGCT** | [hsa-miR-575](http://microrna.sanger.ac.uk/cgi-bin/sequences/mirna_entry.pl?acc=MIMAT0003240) |
| [**rs17104965**](http://www.ncbi.nlm.nih.gov/SNP/snp_ref.cgi?rs=rs17104965) | C/T | PAX9(Paired box protein Pax-9) | **TGTTGCC** | [hsa-miR-196a*](http://microrna.sanger.ac.uk/cgi-bin/sequences/mirna_entry.pl?acc=MIMAT0004562) |
| [**rs17264436**](http://www.ncbi.nlm.nih.gov/SNP/snp_ref.cgi?rs=rs17264436) | A/T | PBRM1(Protein polybromo-1) | **TCACCCA** | [hsa-miR-1262](http://microrna.sanger.ac.uk/cgi-bin/sequences/mirna_entry.pl?acc=MIMAT0005914) |
| [**rs17765013**](http://www.ncbi.nlm.nih.gov/SNP/snp_ref.cgi?rs=rs17765013) | A/G | BACH2(Transcription regulator protein BACH2) | **AGGGATA** | [hsa-miR-188-5p](http://microrna.sanger.ac.uk/cgi-bin/sequences/mirna_entry.pl?acc=MIMAT0000457) |
| [**rs1805100**](http://www.ncbi.nlm.nih.gov/SNP/snp_ref.cgi?rs=rs1805100) | G/A | HNF4G(Hepatocyte nuclear factor 4-gamma) | **CTCAGGA** | [hsa-miR-1200](http://microrna.sanger.ac.uk/cgi-bin/sequences/mirna_entry.pl?acc=MIMAT0005863) |
| [**rs1941084**](http://www.ncbi.nlm.nih.gov/SNP/snp_ref.cgi?rs=rs1941084) | A/G | GATA6(Transcription factor GATA-6) | **GCAGTCC** | [hsa-miR-1288](http://microrna.sanger.ac.uk/cgi-bin/sequences/mirna_entry.pl?acc=MIMAT0005942) |
| [**rs2018650**](http://www.ncbi.nlm.nih.gov/SNP/snp_ref.cgi?rs=rs2018650) | T/C | EHBP1(EH domain-binding protein 1) | **AAACTTT** | [hsa-miR-561](http://microrna.sanger.ac.uk/cgi-bin/sequences/mirna_entry.pl?acc=MIMAT0003225) |
| [**rs2069086**](http://www.ncbi.nlm.nih.gov/SNP/snp_ref.cgi?rs=rs2069086) | T/G | EMP1(Epithelial membrane protein 1) | **ATGGAGA** | [hsa-miR-136](http://microrna.sanger.ac.uk/cgi-bin/sequences/mirna_entry.pl?acc=MIMAT0000448) |
| [**rs2071501**](http://www.ncbi.nlm.nih.gov/SNP/snp_ref.cgi?rs=rs2071501) | T/G | CSK(Tyrosine-protein kinase CSK) | **TTTGAGA** | [hsa-miR-616*](http://microrna.sanger.ac.uk/cgi-bin/sequences/mirna_entry.pl?acc=MIMAT0003284) |
| [**rs2072693**](http://www.ncbi.nlm.nih.gov/SNP/snp_ref.cgi?rs=rs2072693) | C/A | RHCG(Ammonium transporter Rh type C) | **CCTGAGA** | [hsa-miR-1266](http://microrna.sanger.ac.uk/cgi-bin/sequences/mirna_entry.pl?acc=MIMAT0005920) |
| [**rs2073859**](http://www.ncbi.nlm.nih.gov/SNP/snp_ref.cgi?rs=rs2073859) | G/A | LIMK2(LIM domain kinase 2) | **AAGCCAT** | hsa-miR-135 |
| [**rs2075802**](http://www.ncbi.nlm.nih.gov/SNP/snp_ref.cgi?rs=rs2075802) | C/A | KLK9(Kallikrein-9 precursor) | **GGCCACA** | [hsa-miR-663b](http://microrna.sanger.ac.uk/cgi-bin/sequences/mirna_entry.pl?acc=MIMAT0005867) |
| [**rs2078864**](http://www.ncbi.nlm.nih.gov/SNP/snp_ref.cgi?rs=rs2078864) | G/A | ITGA3(Integrin alpha-3 precursor) | **AATGCAG** | [hsa-miR-20a*](http://microrna.sanger.ac.uk/cgi-bin/sequences/mirna_entry.pl?acc=MIMAT0004493) |
| [**rs216598**](http://www.ncbi.nlm.nih.gov/SNP/snp_ref.cgi?rs=rs216598) | G/A | RHBDF1(rhomboid family 1) | **CATCTCA** | [hsa-miR-143](http://microrna.sanger.ac.uk/cgi-bin/sequences/mirna_entry.pl?acc=MIMAT0000435) |
| [**rs2200285**](http://www.ncbi.nlm.nih.gov/SNP/snp_ref.cgi?rs=rs2200285) | T/C | SLC2A12(Solute carrier family 2, facilitated glucose transporter member 12) | **TGCCTTA** | [hsa-miR-124](http://microrna.sanger.ac.uk/cgi-bin/sequences/mirna_entry.pl?acc=MIMAT0000422) |
| [**rs2235882**](http://www.ncbi.nlm.nih.gov/SNP/snp_ref.cgi?rs=rs2235882) | A/G | ENPP5(Ectonucleotide pyrophosphatase/phosphodiesterase family member 5 precursor) | **TGGCACA** | [hsa-miR-1227](http://microrna.sanger.ac.uk/cgi-bin/sequences/mirna_entry.pl?acc=MIMAT0005580) |
| [**rs2241114**](http://www.ncbi.nlm.nih.gov/SNP/snp_ref.cgi?rs=rs2241114) | C/G | AMPH(Amphiphysin) | **TGCCAAA** | [hsa-miR-96](http://microrna.sanger.ac.uk/cgi-bin/sequences/mirna_entry.pl?acc=MIMAT0000095) |
| [**rs2241183**](http://www.ncbi.nlm.nih.gov/SNP/snp_ref.cgi?rs=rs2241183) | A/G | BTBD11(BTB (POZ) domain containing 11 isoform 3) | **TGGTGAA** | [hsa-miR-197](http://microrna.sanger.ac.uk/cgi-bin/sequences/mirna_entry.pl?acc=MIMAT0000227) |
| [**rs2255090**](http://www.ncbi.nlm.nih.gov/SNP/snp_ref.cgi?rs=rs2255090) | G/A | SH3BGRL2(SH3 domain-binding glutamic acid-rich-like protein 2) | **CAAGGAA** | [hsa-miR-502-5p](http://microrna.sanger.ac.uk/cgi-bin/sequences/mirna_entry.pl?acc=MIMAT0002873) |
| [**rs2272669**](http://www.ncbi.nlm.nih.gov/SNP/snp_ref.cgi?rs=rs2272669) | G/A | HNF4G(Hepatocyte nuclear factor 4-gamma) | **GATGGTA** | [hsa-miR-181c*](http://microrna.sanger.ac.uk/cgi-bin/sequences/mirna_entry.pl?acc=MIMAT0004559) |
| [**rs2273847**](http://www.ncbi.nlm.nih.gov/SNP/snp_ref.cgi?rs=rs2273847) | A/G | GALNT12(Polypeptide N-acetylgalactosaminyltransferase 12) | **ATAAGCT** | [hsa-miR-21](http://microrna.sanger.ac.uk/cgi-bin/sequences/mirna_entry.pl?acc=MIMAT0000076) |
| [**rs2280692**](http://www.ncbi.nlm.nih.gov/SNP/snp_ref.cgi?rs=rs2280692) | A/G | SLC8A2(Sodium/calcium exchanger 2 precursor) | **TTACCCA** | [hsa-miR-555](http://microrna.sanger.ac.uk/cgi-bin/sequences/mirna_entry.pl?acc=MIMAT0003219) |
| [**rs2292822**](http://www.ncbi.nlm.nih.gov/SNP/snp_ref.cgi?rs=rs2292822) | C/G | NAV1(Neuron navigator 1) | **CCCCACA** | [hsa-miR-491-5p](http://microrna.sanger.ac.uk/cgi-bin/sequences/mirna_entry.pl?acc=MIMAT0002807) |
| [**rs2307220**](http://www.ncbi.nlm.nih.gov/SNP/snp_ref.cgi?rs=rs2307220) | G/T | PAWR(PRKC apoptosis WT1 regulator protein) | **TTGTATA** | [hsa-miR-381](http://microrna.sanger.ac.uk/cgi-bin/sequences/mirna_entry.pl?acc=MIMAT0000736) |
| [**rs233113**](http://www.ncbi.nlm.nih.gov/SNP/snp_ref.cgi?rs=rs233113) | A/T | DDAH1(NG,NG-dimethylarginine dimethylaminohydrolase 1) | **ATGGGTA** | [hsa-miR-660](http://microrna.sanger.ac.uk/cgi-bin/sequences/mirna_entry.pl?acc=MIMAT0003338) |
| [**rs2377028**](http://www.ncbi.nlm.nih.gov/SNP/snp_ref.cgi?rs=rs2377028) | C/G | LIN9(Lin-9 homolog) | **CCCTGCC** | [hsa-miR-1207-5p](http://microrna.sanger.ac.uk/cgi-bin/sequences/mirna_entry.pl?acc=MIMAT0005871) |
| [**rs2390606**](http://www.ncbi.nlm.nih.gov/SNP/snp_ref.cgi?rs=rs2390606) | G/T | BARHL2(BarH-like 2 homeobox protein) | **AGCTCCC** | [hsa-miR-920](http://microrna.sanger.ac.uk/cgi-bin/sequences/mirna_entry.pl?acc=MIMAT0004970) |
| [**rs2459965**](http://www.ncbi.nlm.nih.gov/SNP/snp_ref.cgi?rs=rs2459965) | T/C | TP53INP1(Tumor protein p53-inducible nuclear protein 1) | **ATTTGTT** | [hsa-miR-7-1*](http://microrna.sanger.ac.uk/cgi-bin/sequences/mirna_entry.pl?acc=MIMAT0004553) |
| [**rs2466551**](http://www.ncbi.nlm.nih.gov/SNP/snp_ref.cgi?rs=rs2466551) | A/C | NFYB(Nuclear transcription factor Y subunit beta) | **ATGCAGA** | [hsa-miR-20a*](http://microrna.sanger.ac.uk/cgi-bin/sequences/mirna_entry.pl?acc=MIMAT0004493) |
| [**rs2497**](http://www.ncbi.nlm.nih.gov/SNP/snp_ref.cgi?rs=rs2497) | C/T | GDI2(Rab GDP dissociation inhibitor beta) | **GTGCCAT** | [hsa-miR-183](http://microrna.sanger.ac.uk/cgi-bin/sequences/mirna_entry.pl?acc=MIMAT0000261) |
| [**rs2506141**](http://www.ncbi.nlm.nih.gov/SNP/snp_ref.cgi?rs=rs2506141) | G/A | NRP1(Neuropilin-1 precursor) | **AGCTGCA** | [hsa-miR-1301](http://microrna.sanger.ac.uk/cgi-bin/sequences/mirna_entry.pl?acc=MIMAT0005797) |
| [**rs2530310**](http://www.ncbi.nlm.nih.gov/SNP/snp_ref.cgi?rs=rs2530310) | C/T | CNTNAP2(Contactin-associated protein-like 2 precursor) | **TTCCAGA** | [hsa-miR-875-3p](http://microrna.sanger.ac.uk/cgi-bin/sequences/mirna_entry.pl?acc=MIMAT0004923) |
| [**rs2550303**](http://www.ncbi.nlm.nih.gov/SNP/snp_ref.cgi?rs=rs2550303) | T/C | AMFR(Autocrine motility factor receptor, isoform 2) | **TTATCTA** | [hsa-miR-577](http://microrna.sanger.ac.uk/cgi-bin/sequences/mirna_entry.pl?acc=MIMAT0003242) |
| [**rs26317**](http://www.ncbi.nlm.nih.gov/SNP/snp_ref.cgi?rs=rs26317) | T/C | MYO10(Myosin-X) | **CACTTTA** | [hsa-miR-17](http://microrna.sanger.ac.uk/cgi-bin/sequences/mirna_entry.pl?acc=MIMAT0000070) |
| [**rs2654981**](http://www.ncbi.nlm.nih.gov/SNP/snp_ref.cgi?rs=rs2654981) | G/C | IGF1R(Insulin-like growth factor 1 receptor precursor) | **GTAGGAA** | [hsa-miR-155*](http://microrna.sanger.ac.uk/cgi-bin/sequences/mirna_entry.pl?acc=MIMAT0004658) |
| [**rs2659582**](http://www.ncbi.nlm.nih.gov/SNP/snp_ref.cgi?rs=rs2659582) | A/G | DOCK5(Dedicator of cytokinesis protein 5) | **ACTACAG** | [hsa-miR-20b*](http://microrna.sanger.ac.uk/cgi-bin/sequences/mirna_entry.pl?acc=MIMAT0004752) |
| [**rs2664370**](http://www.ncbi.nlm.nih.gov/SNP/snp_ref.cgi?rs=rs2664370) | A/G | MMP16(Matrix metalloproteinase-16 precursor) | **CATAGCA** | [hsa-miR-31*](http://microrna.sanger.ac.uk/cgi-bin/sequences/mirna_entry.pl?acc=MIMAT0004504) |
| [**rs2693**](http://www.ncbi.nlm.nih.gov/SNP/snp_ref.cgi?rs=rs2693) | A/G | KIF13B(Kinesin-like protein KIF13B) | **TGTACCA** | [hsa-miR-150*](http://microrna.sanger.ac.uk/cgi-bin/sequences/mirna_entry.pl?acc=MIMAT0004610) |
| [**rs2822638**](http://www.ncbi.nlm.nih.gov/SNP/snp_ref.cgi?rs=rs2822638) | A/G | STCH(Stress 70 protein chaperone microsome-associated 60 kDa protein precursor) | **TATCCAA** | [hsa-miR-1261](http://microrna.sanger.ac.uk/cgi-bin/sequences/mirna_entry.pl?acc=MIMAT0005913) |
| [**rs2941479**](http://www.ncbi.nlm.nih.gov/SNP/snp_ref.cgi?rs=rs2941479) | A/C | HNF4G(Hepatocyte nuclear factor 4-gamma) | **ATTTGAA** | [hsa-miR-607](http://microrna.sanger.ac.uk/cgi-bin/sequences/mirna_entry.pl?acc=MIMAT0003275) |
| [**rs2942**](http://www.ncbi.nlm.nih.gov/SNP/snp_ref.cgi?rs=rs2942) | G/A | GRM1(Metabotropic glutamate receptor 1 precursor) | **AAACCCC** | [hsa-miR-193b*](http://microrna.sanger.ac.uk/cgi-bin/sequences/mirna_entry.pl?acc=MIMAT0004767) |
| [**rs2971877**](http://www.ncbi.nlm.nih.gov/SNP/snp_ref.cgi?rs=rs2971877) | A/G | SPTBN1(Spectrin beta chain, brain 1) | **GACTCAA** | [hsa-miR-627](http://microrna.sanger.ac.uk/cgi-bin/sequences/mirna_entry.pl?acc=MIMAT0003296) |
| [**rs2971879**](http://www.ncbi.nlm.nih.gov/SNP/snp_ref.cgi?rs=rs2971879) | C/T | SPTBN1(Spectrin beta chain, brain 1) | **TCCATGA** | [hsa-miR-490-5p](http://microrna.sanger.ac.uk/cgi-bin/sequences/mirna_entry.pl?acc=MIMAT0004764) |
| [**rs3087960**](http://www.ncbi.nlm.nih.gov/SNP/snp_ref.cgi?rs=rs3087960) | C/G | GOLPH3L(GPP34-related protein) | **GAACCCA** | [hsa-miR-23a*](http://microrna.sanger.ac.uk/cgi-bin/sequences/mirna_entry.pl?acc=MIMAT0004496) |
| [**rs3088034**](http://www.ncbi.nlm.nih.gov/SNP/snp_ref.cgi?rs=rs3088034) | G/C | PHF10(PHD finger protein 10) | **GTTACAT** | [hsa-miR-379*](http://microrna.sanger.ac.uk/cgi-bin/sequences/mirna_entry.pl?acc=MIMAT0004690) |
| [**rs3132555**](http://www.ncbi.nlm.nih.gov/SNP/snp_ref.cgi?rs=rs3132555) | C/G | CDSN(corneodesmosin precursor) | **CCTCCCA** | [hsa-miR-30b*](http://microrna.sanger.ac.uk/cgi-bin/sequences/mirna_entry.pl?acc=MIMAT0004589) |
| [**rs3206635**](http://www.ncbi.nlm.nih.gov/SNP/snp_ref.cgi?rs=rs3206635) | A/G | SMAD5(Mothers against decapentaplegic homolog 5) | **GTAGGCA** | [hsa-miR-24-1*](http://microrna.sanger.ac.uk/cgi-bin/sequences/mirna_entry.pl?acc=MIMAT0000079) |
| [**rs3218210**](http://www.ncbi.nlm.nih.gov/SNP/snp_ref.cgi?rs=rs3218210) | T/A | E2F2(Transcription factor E2F2) | **AGTTTTA** | hsa-miR-548 |
| [**rs3728**](http://www.ncbi.nlm.nih.gov/SNP/snp_ref.cgi?rs=rs3728) | A/C | BRP44L(Brain protein 44-like protein) | **AATAATT** | [hsa-miR-944](http://microrna.sanger.ac.uk/cgi-bin/sequences/mirna_entry.pl?acc=MIMAT0004987) |
| [**rs3733125**](http://www.ncbi.nlm.nih.gov/SNP/snp_ref.cgi?rs=rs3733125) | C/T | ATXN7(Ataxin-7) | **CCCACTG** | [hsa-miR-194*](http://microrna.sanger.ac.uk/cgi-bin/sequences/mirna_entry.pl?acc=MIMAT0004671) |
| [**rs3733176**](http://www.ncbi.nlm.nih.gov/SNP/snp_ref.cgi?rs=rs3733176) | T/C | CD96(T-cell surface protein tactile precursor) | **CCTCTTT** | [hsa-miR-583](http://microrna.sanger.ac.uk/cgi-bin/sequences/mirna_entry.pl?acc=MIMAT0003248) |
| [**rs3734201**](http://www.ncbi.nlm.nih.gov/SNP/snp_ref.cgi?rs=rs3734201) | A/G | GABRR1(Gamma-aminobutyric-acid receptor subunit rho-1 precursor) | **CTACAGA** | [hsa-miR-20b*](http://microrna.sanger.ac.uk/cgi-bin/sequences/mirna_entry.pl?acc=MIMAT0004752) |
| [**rs3739456**](http://www.ncbi.nlm.nih.gov/SNP/snp_ref.cgi?rs=rs3739456) | G/A | AKAP2(Paralemmin-2) | **CGCTGCC** | [hsa-miR-885-3p](http://microrna.sanger.ac.uk/cgi-bin/sequences/mirna_entry.pl?acc=MIMAT0004948) |
| [**rs3740237**](http://www.ncbi.nlm.nih.gov/SNP/snp_ref.cgi?rs=rs3740237) | C/G | EPC1(Enhancer of polycomb homolog 1) | **AACCTGC** | [hsa-miR-657](http://microrna.sanger.ac.uk/cgi-bin/sequences/mirna_entry.pl?acc=MIMAT0003335) |
| [**rs3745550**](http://www.ncbi.nlm.nih.gov/SNP/snp_ref.cgi?rs=rs3745550) | A/G | INSR(Insulin receptor precursor) | **AGCAATA** | [hsa-miR-137](http://microrna.sanger.ac.uk/cgi-bin/sequences/mirna_entry.pl?acc=MIMAT0000429) |
| [**rs3746544**](http://www.ncbi.nlm.nih.gov/SNP/snp_ref.cgi?rs=rs3746544) | T/G | SNAP25(Synaptosomal-associated protein 25) | **ATGTCTT** | [hsa-miR-641](http://microrna.sanger.ac.uk/cgi-bin/sequences/mirna_entry.pl?acc=MIMAT0003311) |
| [**rs3757261**](http://www.ncbi.nlm.nih.gov/SNP/snp_ref.cgi?rs=rs3757261) | C/T | SIRT5(NAD-dependent deacetylase sirtuin-5) | **ACCTCAC** | [hsa-miR-1294](http://microrna.sanger.ac.uk/cgi-bin/sequences/mirna_entry.pl?acc=MIMAT0005884) |
| [**rs3764062**](http://www.ncbi.nlm.nih.gov/SNP/snp_ref.cgi?rs=rs3764062) | A/G | DCLK1(Serine/threonine-protein kinase DCLK1) | **AATCAGA** | [hsa-miR-29a*](http://microrna.sanger.ac.uk/cgi-bin/sequences/mirna_entry.pl?acc=MIMAT0004503) |
| [**rs3774729**](http://www.ncbi.nlm.nih.gov/SNP/snp_ref.cgi?rs=rs3774729) | A/G | ATXN7(Ataxin-7) | **ATGAACA** | [hsa-miR-1206](http://microrna.sanger.ac.uk/cgi-bin/sequences/mirna_entry.pl?acc=MIMAT0005870) |
| [**rs3802266**](http://www.ncbi.nlm.nih.gov/SNP/snp_ref.cgi?rs=rs3802266) | A/G | ZHX2(Zinc fingers and homeoboxes protein 2) | **AATGGGT** | [hsa-miR-660](http://microrna.sanger.ac.uk/cgi-bin/sequences/mirna_entry.pl?acc=MIMAT0003338) |
| [**rs3802703**](http://www.ncbi.nlm.nih.gov/SNP/snp_ref.cgi?rs=rs3802703) | T/C | FAM53B(Protein FAM53B) | **TTGTAGC** | [hsa-miR-187*](http://microrna.sanger.ac.uk/cgi-bin/sequences/mirna_entry.pl?acc=MIMAT0004561) |
| [**rs3809401**](http://www.ncbi.nlm.nih.gov/SNP/snp_ref.cgi?rs=rs3809401) | G/A | SLC8A3(Sodium/calcium exchanger 3 precursor) | **ATAGGAA** | [hsa-miR-337-3p](http://microrna.sanger.ac.uk/cgi-bin/sequences/mirna_entry.pl?acc=MIMAT0000754) |
| [**rs3809724**](http://www.ncbi.nlm.nih.gov/SNP/snp_ref.cgi?rs=rs3809724) | G/A | PPM1E(Protein phosphatase 1E) | **ACTTCAA** | [hsa-miR-573](http://microrna.sanger.ac.uk/cgi-bin/sequences/mirna_entry.pl?acc=MIMAT0003238) |
| [**rs3813034**](http://www.ncbi.nlm.nih.gov/SNP/snp_ref.cgi?rs=rs3813034) | T/G | SLC6A4(Sodium-dependent serotonin transporter) | **ATTAACA** | [hsa-miR-569](http://microrna.sanger.ac.uk/cgi-bin/sequences/mirna_entry.pl?acc=MIMAT0003234) |
| [**rs3825195**](http://www.ncbi.nlm.nih.gov/SNP/snp_ref.cgi?rs=rs3825195) | T/C | PDE1B(Calcium/calmodulin-dependent 3',5'-cyclic nucleotide phosphodiesterase 1B) | **TAGCCAG** | [hsa-miR-664*](http://microrna.sanger.ac.uk/cgi-bin/sequences/mirna_entry.pl?acc=MIMAT0005948) |
| [**rs4016**](http://www.ncbi.nlm.nih.gov/SNP/snp_ref.cgi?rs=rs4016) | T/A | PGRMC2(Membrane-associated progesterone receptor component 2) | **ATGCAGT** | [hsa-miR-217](http://microrna.sanger.ac.uk/cgi-bin/sequences/mirna_entry.pl?acc=MIMAT0000274) |
| [**rs41739**](http://www.ncbi.nlm.nih.gov/SNP/snp_ref.cgi?rs=rs41739) | A/G | MET(Hepatocyte growth factor receptor precursor) | **TTAGAAA** | [hsa-miR-576-5p](http://microrna.sanger.ac.uk/cgi-bin/sequences/mirna_entry.pl?acc=MIMAT0003241) |
| [**rs4447076**](http://www.ncbi.nlm.nih.gov/SNP/snp_ref.cgi?rs=rs4447076) | T/C | MMRN2(Multimerin-2 precursor) | **CTTCTGC** | [hsa-miR-298](http://microrna.sanger.ac.uk/cgi-bin/sequences/mirna_entry.pl?acc=MIMAT0004901) |
| [**rs444927**](http://www.ncbi.nlm.nih.gov/SNP/snp_ref.cgi?rs=rs444927) | A/G | CNTN1(Contactin-1 precursor) | **TCAGGAA** | [hsa-miR-1200](http://microrna.sanger.ac.uk/cgi-bin/sequences/mirna_entry.pl?acc=MIMAT0005863) |
| [**rs4560**](http://www.ncbi.nlm.nih.gov/SNP/snp_ref.cgi?rs=rs4560) | G/A | EIF2AK1(Eukaryotic translation initiation factor 2-alpha kinase 1) | **CCAGGTT** | [hsa-miR-490-3p](http://microrna.sanger.ac.uk/cgi-bin/sequences/mirna_entry.pl?acc=MIMAT0002806) |
| [**rs4796030**](http://www.ncbi.nlm.nih.gov/SNP/snp_ref.cgi?rs=rs4796030) | C/A | LIG3(DNA ligase 3) | **AAGCCCA** | [hsa-miR-27a*](http://microrna.sanger.ac.uk/cgi-bin/sequences/mirna_entry.pl?acc=MIMAT0004501) |
| [**rs4823006**](http://www.ncbi.nlm.nih.gov/SNP/snp_ref.cgi?rs=rs4823006) | A/G | ZNRF3(Zinc/RING finger protein 3 precursor) | **GCCAGGA** | [hsa-miR-221*](http://microrna.sanger.ac.uk/cgi-bin/sequences/mirna_entry.pl?acc=MIMAT0004568) |
| [**rs485609**](http://www.ncbi.nlm.nih.gov/SNP/snp_ref.cgi?rs=rs485609) | C/G | SCARA3(Scavenger receptor class A member 3) | **CAGAGAC** | [hsa-miR-593](http://microrna.sanger.ac.uk/cgi-bin/sequences/mirna_entry.pl?acc=MIMAT0004802) |
| [**rs4902357**](http://www.ncbi.nlm.nih.gov/SNP/snp_ref.cgi?rs=rs4902357) | C/G | MAX(Protein max) | **GATCCCA** | [hsa-miR-638](http://microrna.sanger.ac.uk/cgi-bin/sequences/mirna_entry.pl?acc=MIMAT0003308) |
| [**rs495714**](http://www.ncbi.nlm.nih.gov/SNP/snp_ref.cgi?rs=rs495714) | G/A | ABCB11(Bile salt export pump) | **AGGGCAG** | [hsa-miR-18a*](http://microrna.sanger.ac.uk/cgi-bin/sequences/mirna_entry.pl?acc=MIMAT0002891) |
| [**rs551517**](http://www.ncbi.nlm.nih.gov/SNP/snp_ref.cgi?rs=rs551517) | G/A | EDG2(Lysophosphatidic acid receptor Edg-2) | **TGCAGAA** | [hsa-miR-544](http://microrna.sanger.ac.uk/cgi-bin/sequences/mirna_entry.pl?acc=MIMAT0003164) |
| [**rs587404**](http://www.ncbi.nlm.nih.gov/SNP/snp_ref.cgi?rs=rs587404) | A/G | MACF1(Microtubule-actin cross-linking factor 1) | **GAAACCA** | [hsa-miR-29b-2*](http://microrna.sanger.ac.uk/cgi-bin/sequences/mirna_entry.pl?acc=MIMAT0004515) |
| [**rs602201**](http://www.ncbi.nlm.nih.gov/SNP/snp_ref.cgi?rs=rs602201) | T/A | GNAL(Guanine nucleotide-binding protein G(olf) subunit alpha) | **ATGTTTC** | [hsa-miR-494](http://microrna.sanger.ac.uk/cgi-bin/sequences/mirna_entry.pl?acc=MIMAT0002816) |
| [**rs608823**](http://www.ncbi.nlm.nih.gov/SNP/snp_ref.cgi?rs=rs608823) | G/A | ONECUT2(One cut domain family member 2) | **GAAGTAG** | [hsa-miR-138-1*](http://microrna.sanger.ac.uk/cgi-bin/sequences/mirna_entry.pl?acc=MIMAT0004607) |
| [**rs6128327**](http://www.ncbi.nlm.nih.gov/SNP/snp_ref.cgi?rs=rs6128327) | A/G | RAB22A(Ras-related protein Rab-22A) | **GTTTACA** | hsa-miR-30 |
| [**rs6480718**](http://www.ncbi.nlm.nih.gov/SNP/snp_ref.cgi?rs=rs6480718) | C/G | AP3M1(AP-3 complex subunit mu-1) | **GCAAAAA** | [hsa-miR-129-5p](http://microrna.sanger.ac.uk/cgi-bin/sequences/mirna_entry.pl?acc=MIMAT0000242) |
| [**rs6566883**](http://www.ncbi.nlm.nih.gov/SNP/snp_ref.cgi?rs=rs6566883) | G/A | ONECUT2(One cut domain family member 2) | **GAGGGAA** | [hsa-miR-642](http://microrna.sanger.ac.uk/cgi-bin/sequences/mirna_entry.pl?acc=MIMAT0003312) |
| [**rs6821591**](http://www.ncbi.nlm.nih.gov/SNP/snp_ref.cgi?rs=rs6821591) | A/G | PPARGC1A(Peroxisome proliferator-activated receptor gamma coactivator 1-alpha) | **ACACTTA** | [hsa-miR-595](http://microrna.sanger.ac.uk/cgi-bin/sequences/mirna_entry.pl?acc=MIMAT0003263) |
| [**rs6845**](http://www.ncbi.nlm.nih.gov/SNP/snp_ref.cgi?rs=rs6845) | C/T | PSMD2(26S proteasome non-ATPase regulatory subunit 2) | **CAGCTGA** | [hsa-miR-1207-3p](http://microrna.sanger.ac.uk/cgi-bin/sequences/mirna_entry.pl?acc=MIMAT0005872) |
| [**rs6923492**](http://www.ncbi.nlm.nih.gov/SNP/snp_ref.cgi?rs=rs6923492) | C/T | GRM1(Metabotropic glutamate receptor 1 precursor) | **CCCCACC** | [hsa-miR-92a-2*](http://microrna.sanger.ac.uk/cgi-bin/sequences/mirna_entry.pl?acc=MIMAT0004508) |
| [**rs6958**](http://www.ncbi.nlm.nih.gov/SNP/snp_ref.cgi?rs=rs6958) | C/G | PRKAR1A(cAMP-dependent protein kinase type I-alpha regulatory subunit) | **TGGCCAA** | [hsa-miR-588](http://microrna.sanger.ac.uk/cgi-bin/sequences/mirna_entry.pl?acc=MIMAT0003255) |
| [**rs6960867**](http://www.ncbi.nlm.nih.gov/SNP/snp_ref.cgi?rs=rs6960867) | A/G | AKAP9(A-kinase anchor protein 9) | **AATCAGA** | [hsa-miR-29a*](http://microrna.sanger.ac.uk/cgi-bin/sequences/mirna_entry.pl?acc=MIMAT0004503) |
| [**rs698761**](http://www.ncbi.nlm.nih.gov/SNP/snp_ref.cgi?rs=rs698761) | C/T | PREPL(prolyl endopeptidase-like isoform D) | **ATTCTCA** | [hsa-miR-580](http://microrna.sanger.ac.uk/cgi-bin/sequences/mirna_entry.pl?acc=MIMAT0003245) |
| [**rs701848**](http://www.ncbi.nlm.nih.gov/SNP/snp_ref.cgi?rs=rs701848) | T/C | PTEN(Phosphatase and tensin homolog) | **GCTTCAA** | [hsa-miR-127-5p](http://microrna.sanger.ac.uk/cgi-bin/sequences/mirna_entry.pl?acc=MIMAT0004604) |
| [**rs707718**](http://www.ncbi.nlm.nih.gov/SNP/snp_ref.cgi?rs=rs707718) | A/C | CYP26B1(Cytochrome P450 26B1) | **AATGAAT** | [hsa-miR-664](http://microrna.sanger.ac.uk/cgi-bin/sequences/mirna_entry.pl?acc=MIMAT0005949) |
| [**rs709592**](http://www.ncbi.nlm.nih.gov/SNP/snp_ref.cgi?rs=rs709592) | G/A | MED24(Thyroid hormone receptor-associated protein complex 100 kDa component) | **TGAGCAG** | [hsa-miR-767-3p](http://microrna.sanger.ac.uk/cgi-bin/sequences/mirna_entry.pl?acc=MIMAT0003883) |
| [**rs7132908**](http://www.ncbi.nlm.nih.gov/SNP/snp_ref.cgi?rs=rs7132908) | C/T | (FAIM2)Fas apoptotic inhibitory molecule 2 | **CCCAGAG** | [hsa-miR-326](http://microrna.sanger.ac.uk/cgi-bin/sequences/mirna_entry.pl?acc=MIMAT0000756) |
| [**rs715020**](http://www.ncbi.nlm.nih.gov/SNP/snp_ref.cgi?rs=rs715020) | A/G | QKI(Quaking protein) | **AAATGAA** | [hsa-miR-579](http://microrna.sanger.ac.uk/cgi-bin/sequences/mirna_entry.pl?acc=MIMAT0003244) |
| [**rs7221**](http://www.ncbi.nlm.nih.gov/SNP/snp_ref.cgi?rs=rs7221) | T/C | MBNL1(Muscleblind-like protein 1) | **TTTGCTG** | [hsa-miR-545](http://microrna.sanger.ac.uk/cgi-bin/sequences/mirna_entry.pl?acc=MIMAT0003165) |
| [**rs7237996**](http://www.ncbi.nlm.nih.gov/SNP/snp_ref.cgi?rs=rs7237996) | A/G | ONECUT2(One cut domain family member 2) | **GTGCATA** | [hsa-miR-501-3p](http://microrna.sanger.ac.uk/cgi-bin/sequences/mirna_entry.pl?acc=MIMAT0004774) |
| [**rs7359387**](http://www.ncbi.nlm.nih.gov/SNP/snp_ref.cgi?rs=rs7359387) | T/G | NFAT5(Nuclear factor of activated T-cells 5) | **GTTACAT** | [hsa-miR-379*](http://microrna.sanger.ac.uk/cgi-bin/sequences/mirna_entry.pl?acc=MIMAT0004690) |
| [**rs7549683**](http://www.ncbi.nlm.nih.gov/SNP/snp_ref.cgi?rs=rs7549683) | A/C | GCLM(Glutamate--cysteine ligase modifier subunit) | **TTTAAGA** | [hsa-miR-302a*](http://microrna.sanger.ac.uk/cgi-bin/sequences/mirna_entry.pl?acc=MIMAT0000683) |
| [**rs7566**](http://www.ncbi.nlm.nih.gov/SNP/snp_ref.cgi?rs=rs7566) | C/T | CANX(Calnexin precursor) | **GAATGCT** | [hsa-miR-1179](http://microrna.sanger.ac.uk/cgi-bin/sequences/mirna_entry.pl?acc=MIMAT0005824) |
| [**rs7655413**](http://www.ncbi.nlm.nih.gov/SNP/snp_ref.cgi?rs=rs7655413) | C/T | FGF2(Heparin-binding growth factor 2 precursor) | **CATTTGA** | [hsa-miR-105](http://microrna.sanger.ac.uk/cgi-bin/sequences/mirna_entry.pl?acc=MIMAT0000102) |
| [**rs767053**](http://www.ncbi.nlm.nih.gov/SNP/snp_ref.cgi?rs=rs767053) | C/T | RC3H1(RING finger and C3H zinc finger protein 1) | **AACCTGC** | [hsa-miR-657](http://microrna.sanger.ac.uk/cgi-bin/sequences/mirna_entry.pl?acc=MIMAT0003335) |
| [**rs786906**](http://www.ncbi.nlm.nih.gov/SNP/snp_ref.cgi?rs=rs786906) | T/C | PKN2(Serine/threonine-protein kinase N2) | **AGTGATT** | [hsa-miR-34b](http://microrna.sanger.ac.uk/cgi-bin/sequences/mirna_entry.pl?acc=MIMAT0004676) |
| [**rs7974459**](http://www.ncbi.nlm.nih.gov/SNP/snp_ref.cgi?rs=rs7974459) | C/T | KCNA1(Potassium voltage-gated channel subfamily A member 1) | **ACTGCAG** | [hsa-miR-17*](http://microrna.sanger.ac.uk/cgi-bin/sequences/mirna_entry.pl?acc=MIMAT0000071) |
| [**rs7974559**](http://www.ncbi.nlm.nih.gov/SNP/snp_ref.cgi?rs=rs7974559) | A/T | KCNA1(Potassium voltage-gated channel subfamily A member 1) | **AACTGGA** | [hsa-miR-145](http://microrna.sanger.ac.uk/cgi-bin/sequences/mirna_entry.pl?acc=MIMAT0000437) |
| [**rs8031107**](http://www.ncbi.nlm.nih.gov/SNP/snp_ref.cgi?rs=rs8031107) | G/A | IL16(Interleukin-16 precursor) | **CAGTGGA** | [hsa-miR-181a-2*](http://microrna.sanger.ac.uk/cgi-bin/sequences/mirna_entry.pl?acc=MIMAT0004558) |
| [**rs8031627**](http://www.ncbi.nlm.nih.gov/SNP/snp_ref.cgi?rs=rs8031627) | G/A | SMAD3(Mothers against decapentaplegic homolog 3) | **GCAGGCT** | [hsa-miR-596](http://microrna.sanger.ac.uk/cgi-bin/sequences/mirna_entry.pl?acc=MIMAT0003264) |
| [**rs8203**](http://www.ncbi.nlm.nih.gov/SNP/snp_ref.cgi?rs=rs8203) | A/G | PHLPPL(PH domain leucine-rich repeat protein phosphatase-like) | **AATAATA** | [hsa-miR-126*](http://microrna.sanger.ac.uk/cgi-bin/sequences/mirna_entry.pl?acc=MIMAT0000444) |
| [**rs8336**](http://www.ncbi.nlm.nih.gov/SNP/snp_ref.cgi?rs=rs8336) | C/T | SMARCAD1(SWI/SNF-related matrix-associated actin-dependent regulator) | **ACTTCAA** | [hsa-miR-573](http://microrna.sanger.ac.uk/cgi-bin/sequences/mirna_entry.pl?acc=MIMAT0003238) |
| [**rs8410**](http://www.ncbi.nlm.nih.gov/SNP/snp_ref.cgi?rs=rs8410) | A/G | PREPL(prolyl endopeptidase-like isoform D) | **ATGCATA** | [hsa-miR-586](http://microrna.sanger.ac.uk/cgi-bin/sequences/mirna_entry.pl?acc=MIMAT0003252) |
| [**rs8461**](http://www.ncbi.nlm.nih.gov/SNP/snp_ref.cgi?rs=rs8461) | G/A | PISD(Phosphatidylserine decarboxylase proenzyme) | **TGCCTCC** | [hsa-miR-650](http://microrna.sanger.ac.uk/cgi-bin/sequences/mirna_entry.pl?acc=MIMAT0003320) |
| [**rs854802**](http://www.ncbi.nlm.nih.gov/SNP/snp_ref.cgi?rs=rs854802) | A/G | MYO15A(Myosin-XV) | **AATGAAT** | [hsa-miR-664](http://microrna.sanger.ac.uk/cgi-bin/sequences/mirna_entry.pl?acc=MIMAT0005949) |
| [**rs8602**](http://www.ncbi.nlm.nih.gov/SNP/snp_ref.cgi?rs=rs8602) | G/T | MKNK1(MAP kinase-interacting serine/threonine-protein kinase 1) | **TCCAGCA** | [hsa-miR-1287](http://microrna.sanger.ac.uk/cgi-bin/sequences/mirna_entry.pl?acc=MIMAT0005878) |
| [**rs8752**](http://www.ncbi.nlm.nih.gov/SNP/snp_ref.cgi?rs=rs8752) | G/A | HPGD(15-hydroxyprostaglandin dehydrogenase) | **CAGCCTC** | [hsa-miR-485-5p](http://microrna.sanger.ac.uk/cgi-bin/sequences/mirna_entry.pl?acc=MIMAT0002175) |
| [**rs882869**](http://www.ncbi.nlm.nih.gov/SNP/snp_ref.cgi?rs=rs882869) | A/G | FMN2(Formin-2) | **TGCATAA** | [hsa-miR-491-3p](http://microrna.sanger.ac.uk/cgi-bin/sequences/mirna_entry.pl?acc=MIMAT0004765) |
| [**rs887842**](http://www.ncbi.nlm.nih.gov/SNP/snp_ref.cgi?rs=rs887842) | A/C | CYP26B1(Cytochrome P450 26B1) | **AATATGA** | [hsa-miR-1279](http://microrna.sanger.ac.uk/cgi-bin/sequences/mirna_entry.pl?acc=MIMAT0005937) |
| [**rs9293360**](http://www.ncbi.nlm.nih.gov/SNP/snp_ref.cgi?rs=rs9293360) | T/C | EDIL3(EGF-like repeat and discoidin I-like domain-containing protein 3 precursor) | **TGGCCAA** | [hsa-miR-588](http://microrna.sanger.ac.uk/cgi-bin/sequences/mirna_entry.pl?acc=MIMAT0003255) |
| [**rs9332**](http://www.ncbi.nlm.nih.gov/SNP/snp_ref.cgi?rs=rs9332) | G/A | MTRR(Methionine synthase reductase, mitochondrial precursor) | **TCTGATA** | [hsa-miR-361-5p](http://microrna.sanger.ac.uk/cgi-bin/sequences/mirna_entry.pl?acc=MIMAT0000703) |
| [**rs9556713**](http://www.ncbi.nlm.nih.gov/SNP/snp_ref.cgi?rs=rs9556713) | A/G | MBNL2(Muscleblind-like protein 2) | **GAATGTA** | hsa-miR-181 |
| [**rs9611591**](http://www.ncbi.nlm.nih.gov/SNP/snp_ref.cgi?rs=rs9611591) | C/T | TOB2(Protein Tob2) | **GGGCCCA** | [hsa-miR-296-5p](http://microrna.sanger.ac.uk/cgi-bin/sequences/mirna_entry.pl?acc=MIMAT0000690) |
| [**rs9614613**](http://www.ncbi.nlm.nih.gov/SNP/snp_ref.cgi?rs=rs9614613) | G/C | NUP50(Nucleoporin 50 kDa) | **CAGGGTA** | hsa-miR-10 |
| [**rs963917**](http://www.ncbi.nlm.nih.gov/SNP/snp_ref.cgi?rs=rs963917) | G/A | RAD51L1(DNA repair protein RAD51 homolog 2) | **CCTGAGT** | [hsa-miR-510](http://microrna.sanger.ac.uk/cgi-bin/sequences/mirna_entry.pl?acc=MIMAT0002882) |
| [**rs963918**](http://www.ncbi.nlm.nih.gov/SNP/snp_ref.cgi?rs=rs963918) | T/C | RAD51L1(DNA repair protein RAD51 homolog 2) | **CTATGAA** | hsa-miR-376 |
| [**rs9731**](http://www.ncbi.nlm.nih.gov/SNP/snp_ref.cgi?rs=rs9731) | A/G | CLDN12(Claudin-12) | **AAGCCAT** | hsa-miR-135 |
| [**rs9844202**](http://www.ncbi.nlm.nih.gov/SNP/snp_ref.cgi?rs=rs9844202) | A/G | SERPINI2(Serpin I2 precursor) | **TCATCAT** | [hsa-miR-1272](http://microrna.sanger.ac.uk/cgi-bin/sequences/mirna_entry.pl?acc=MIMAT0005925) |
