# Supplementary material for: SNPs within microRNA binding sites and the prognosis of breast cancer
Source: Aging (Albany NY). 2021 Feb 26;13(5):7465–80. doi: 10.18632/aging.202612 (PMC7993692; doi:10.18632/aging.202612)
Supplement: Supplementary Table 2 [file aging-13-202612-s003.docx]

**Supplementary Table 2. Association between 192 SNPs within microRNA binding sites and breast cancer OS (Stage I).**

| Polymorphisms | *N* (%) | Univariate Cox regression | |  | Multivariate Cox regression | |
| --- | --- | --- | --- | --- | --- | --- |
|  |  | HR (95% CI) | *P* |  | HR (95% CI) | *P* |
| rs3802266 |  |  |  |  |  |  |
| AA | 675 (52.1) | 1 (ref) |  |  | 1 (ref) |  |
| AG | 520 (40.2) | 0.74 (0.46-1.19) | 0.216 |  | 0.80 (0.48-1.34) | 0.397 |
| GG | 100 (7.7) | 1.10 (0.52-2.34) | 0.799 |  | 1.31 (0.61-2.81) | 0.495 |
| rs6821591 |  |  |  |  |  |  |
| AA | 644 (49.7) | 1 (ref) |  |  | 1 (ref) |  |
| AG | 532 (41.0) | 0.94 (0.59-1.51) | 0.806 |  | 0.92 (0.56-1.52) | 0.755 |
| GG | 121 (9.3) | 1.42 (0.69-2.93) | 0.340 |  | 1.50 (0.72-3.13) | 0.280 |
| **rs1053739** |  |  |  |  |  |  |
| AA | 359 (27.7) | 1 (ref) |  |  | 1 (ref) |  |
| AG | 659 (50.8) | 1.64 (0.88-3.07) | 0.121 |  | 2.65 (0.98-7.10) | 0.053 |
| GG | 278 (21.5) | **2.66 (1.37-5.16)** | **0.004** |  | **4.38 (1.52-12.65)** | **0.006** |
| rs1056471 |  |  |  |  |  |  |
| GG | 839 (64.9) | 1 (ref) |  |  | 1 (ref) |  |
| CG | 367 (28.4) | 1.26 (0.78-2.03) | 0.353 |  | 1.23 (0.74-2.07) | 0.425 |
| CC | 87 (6.7) | 1.73 (0.82-3.68) | 0.152 |  | 1.84 (0.86-3.95) | 0.117 |
| rs7974559 |  |  |  |  |  |  |
| AA | 451 (34.8) | 1 (ref) |  |  | 1 (ref) |  |
| AT | 614 (47.4) | 0.98 (0.61-1.58) | 0.923 |  | 0.89 (0.54-1.45) | 0.632 |
| TT | 230 (17.8) | 0.74 (0.37-1.47) | 0.382 |  | 0.45 (0.20-1.02) | 0.057 |
| rs707718 |  |  |  |  |  |  |
| AA | 375 (28.9) | 1 (ref) |  |  | 1 (ref) |  |
| AC | 649 (50.0) | 0.76 (0.47-1.22) | 0.254 |  | 0.76 (0.46-1.26) | 0.288 |
| CC | 273 (21.0) | 0.69 (0.36-1.33) | 0.269 |  | 0.51 (0.24-1.10) | 0.085 |
| rs12636077 |  |  |  |  |  |  |
| GG | 831 (64.1) | 1 (ref) |  |  | 1 (ref) |  |
| AG | 420 (32.4) | 1.14 (0.72-1.82) | 0.582 |  | 1.18 (0.72-1.94) | 0.516 |
| AA | 46 (3.5) | 1.58 (0.57-4.37) | 0.383 |  | 1.51 (0.54-4.24) | 0.431 |
| rs1044145 |  |  |  |  |  |  |
| AA | 780 (60.1) | 1 (ref) |  |  | 1 (ref) |  |
| AG | 465 (35.9) | 1.29 (0.82-2.03) | 0.270 |  | 1.19 (0.74-1.92) | 0.469 |
| GG | 52 (4.0) | 1.08 (0.33-3.47) | 0.902 |  | 0.83 (0.20-3.46) | 0.795 |
| rs1131382 |  |  |  |  |  |  |
| AA | 324 (25.2) | 1 (ref) |  |  | 1 (ref) |  |
| AG | 666 (51.7) | 0.95 (0.57-1.59) | 0.846 |  | 0.83 (0.48-1.44) | 0.509 |
| GG | 298 (23.1) | 0.72 (0.37-1.4) | 0.331 |  | 0.71 (0.36-1.4) | 0.322 |
| rs3733176 |  |  |  |  |  |  |
| GG | 751 (57.9) | 1 (ref) |  |  | 1 (ref) |  |
| AG | 472 (36.4) | 1.03 (0.65-1.63) | 0.891 |  | 0.95 (0.59-1.56) | 0.850 |
| AA | 74 (5.7) | 0.73 (0.23-2.33) | 0.589 |  | 0.80 (0.25-2.61) | 0.717 |
| rs9293360 |  |  |  |  |  |  |
| GG | 399 (30.9) | 1 (ref) |  |  | 1 (ref) |  |
| AG | 637 (49.3) | 0.86 (0.52-1.44) | 0.572 |  | 0.85 (0.49-1.47) | 0.559 |
| AA | 257 (19.9) | 0.91 (0.49-1.71) | 0.769 |  | 1.02 (0.53-1.96) | 0.949 |
| rs10437 |  |  |  |  |  |  |
| AA | 817 (63.0) | 1 (ref) |  |  | 1 (ref) |  |
| AG | 430 (33.2) | 1.01 (0.64-1.6) | 0.967 |  | 0.85 (0.52-1.40) | 0.533 |
| GG | 50 (3.9) | 0.30 (0.04-2.2) | 0.238 |  | 0.31 (0.04-2.28) | 0.251 |
| rs2241183 |  |  |  |  |  |  |
| GG | 476 (36.7) | 1 (ref) |  |  | 1 (ref) |  |
| AG | 616 (47.5) | 1.25 (0.78-2.01) | 0.359 |  | 1.16 (0.70-1.92) | 0.558 |
| AA | 204 (15.7) | 0.65 (0.29-1.42) | 0.278 |  | 0.62 (0.27-1.43) | 0.260 |
| rs1235 |  |  |  |  |  |  |
| AA | 724 (55.8) | 1 (ref) |  |  | 1 (ref) |  |
| AC | 489 (37.7) | 1.34 (0.86-2.11) | 0.200 |  | 1.10 (0.68-1.78) | 0.699 |
| CC | 84 (6.5) | 0.94 (0.34-2.65) | 0.913 |  | 0.91 (0.32-2.57) | 0.860 |
| rs8031627 |  |  |  |  |  |  |
| AA | 336 (25.9) | 1 (ref) |  |  | 1 (ref) |  |
| AG | 649 (50.1) | 0.92 (0.53-1.58) | 0.750 |  | 0.98 (0.56-1.74) | 0.954 |
| GG | 310 (23.9) | 1.32 (0.72-2.40) | 0.366 |  | 1.15 (0.61-2.19) | 0.661 |
| rs709592 |  |  |  |  |  |  |
| GG | 407 (31.5) | 1 (ref) |  |  | 1 (ref) |  |
| AG | 651 (50.3) | 1.13 (0.68-1.88) | 0.649 |  | 1.12 (0.65-1.91) | 0.694 |
| AA | 236 (18.2) | 1.33 (0.71-2.49) | 0.372 |  | 1.32 (0.68-2.59) | 0.411 |
| rs602201 |  |  |  |  |  |  |
| TT | 523 (40.4) | 1 (ref) |  |  | 1 (ref) |  |
| AT | 581 (44.9) | 1.51 (0.93-2.47) | 0.098 |  | 1.51 (0.91-2.51) | 0.114 |
| AA | 189 (14.6) | 1.27 (0.63-2.58) | 0.503 |  | 1.35 (0.62-2.93) | 0.452 |
| rs3739456 |  |  |  |  |  |  |
| GG | 582 (44.9) | 1 (ref) |  |  | 1 (ref) |  |
| AG | 583 (45.0) | 1.02 (0.65-1.6) | 0.925 |  | 1.02 (0.63-1.65) | 0.938 |
| AA | 130 (10.0) | 0.47 (0.17-1.32) | 0.150 |  | 0.49 (0.17-1.4) | 0.183 |
| rs3746544 |  |  |  |  |  |  |
| AA | 762 (58.8) | 1 (ref) |  |  | 1 (ref) |  |
| AC | 456 (35.2) | 0.87 (0.54-1.42) | 0.588 |  | 0.89 (0.53-1.50) | 0.672 |
| CC | 78 (6.0) | 1.18 (0.50-2.74) | 0.709 |  | 1.26 (0.53-3.02) | 0.599 |
| rs10305516 |  |  |  |  |  |  |
| GG | 588 (45.5) | 1 (ref) |  |  | 1 (ref) |  |
| CG | 567 (43.9) | 0.86 (0.54-1.36) | 0.516 |  | 0.90 (0.56-1.47) | 0.680 |
| CC | 137 (10.6) | 0.63 (0.27-1.49) | 0.293 |  | 0.67 (0.28-1.6) | 0.364 |
| **rs2693** |  |  |  |  |  |  |
| GG | 656 (50.6) | 1 (ref) |  |  | 1 (ref) |  |
| AG | 532 (41.0) | 1.08 (0.67-1.74) | 0.754 |  | 0.99 (0.50-1.95) | 0.975 |
| AA | 108 (8.3) | **2.35 (1.22-4.51)** | **0.011** |  | **3.19 (1.01-10.04)** | **0.047** |
| rs3088034 |  |  |  |  |  |  |
| GG | 583 (44.9) | 1 (ref) |  |  | 1 (ref) |  |
| CG | 562 (43.3) | 1.08 (0.68-1.71) | 0.756 |  | 1.25 (0.76-2.05) | 0.382 |
| CC | 152 (11.7) | 0.90 (0.44-1.87) | 0.783 |  | 1.00 (0.47-2.12) | 0.996 |
| rs2073859 |  |  |  |  |  |  |
| GG | 524 (40.4) | 1 (ref) |  |  | 1 (ref) |  |
| AG | 585 (45.1) | 1.19 (0.73-1.94) | 0.477 |  | 1.23 (0.73-2.06) | 0.435 |
| AA | 187 (14.4) | 1.34 (0.69-2.58) | 0.389 |  | 1.38 (0.69-2.78) | 0.363 |
| rs767053 |  |  |  |  |  |  |
| GG | 641 (49.6) | 1 (ref) |  |  | 1 (ref) |  |
| AG | 536 (41.5) | 0.80 (0.5-1.28) | 0.352 |  | 0.92 (0.57-1.51) | 0.754 |
| AA | 115 (8.9) | 0.88 (0.4-1.95) | 0.751 |  | 1.00 (0.44-2.26) | 0.999 |
| rs495714 |  |  |  |  |  |  |
| AA | 478 (36.9) | 1 (ref) |  |  | 1 (ref) |  |
| AG | 607 (46.9) | 1.20 (0.74-1.93) | 0.469 |  | 1.25 (0.75-2.09) | 0.387 |
| GG | 210 (16.2) | 0.83 (0.40-1.70) | 0.608 |  | 0.82 (0.38-1.75) | 0.600 |
| rs4560 |  |  |  |  |  |  |
| GG | 396 (30.5) | 1 (ref) |  |  | 1 (ref) |  |
| AG | 647 (49.9) | 1.04 (0.63-1.72) | 0.875 |  | 1.13 (0.66-1.94) | 0.658 |
| AA | 254 (19.6) | 0.83 (0.43-1.60) | 0.577 |  | 0.91 (0.45-1.84) | 0.799 |
| rs7549683 |  |  |  |  |  |  |
| CC | 804 (62.0) | 1 (ref) |  |  | 1 (ref) |  |
| AC | 417 (32.2) | 1.26 (0.80-2.00) | 0.321 |  | 1.18 (0.72-1.93) | 0.511 |
| AA | 76 (5.9) | 0.91 (0.33-2.52) | 0.853 |  | 1.01 (0.36-2.86) | 0.986 |
| rs9844202 |  |  |  |  |  |  |
| GG | 623 (48.1) | 1 (ref) |  |  | 1 (ref) |  |
| AG | 548 (42.3) | 1.03 (0.64-1.66) | 0.920 |  | 1.05 (0.63-1.75) | 0.860 |
| AA | 124 (9.6) | 1.79 (0.95-3.39) | 0.074 |  | 1.97 (1.02-3.83) | 0.044 |
| rs1043915 |  |  |  |  |  |  |
| AA | 411 (31.7) | 1 (ref) |  |  | 1 (ref) |  |
| AT | 667 (51.5) | 1.08 (0.66-1.77) | 0.773 |  | 0.91 (0.54-1.51) | 0.707 |
| TT | 217 (16.8) | 1.05 (0.54-2.04) | 0.896 |  | 0.75 (0.36-1.56) | 0.434 |
| rs8203 |  |  |  |  |  |  |
| GG | 733 (56.6) | 1 (ref) |  |  | 1 (ref) |  |
| AG | 491 (37.9) | 1.05 (0.67-1.65) | 0.839 |  | 1.16 (0.71-1.88) | 0.553 |
| AA | 70 (5.4) | 0.91 (0.33-2.52) | 0.851 |  | 1.13 (0.40-3.20) | 0.818 |
| rs1941084 |  |  |  |  |  |  |
| GG | 396 (30.7) | 1 (ref) |  |  | 1 (ref) |  |
| AG | 675 (52.2) | 1.05 (0.63-1.74) | 0.855 |  | 1.19 (0.70-2.05) | 0.520 |
| AA | 221 (17.1) | 1.16 (0.61-2.23) | 0.653 |  | 1.20 (0.59-2.44) | 0.616 |
| rs1051424 |  |  |  |  |  |  |
| AA | 438 (33.8) | 1 (ref) |  |  | 1 (ref) |  |
| AG | 628 (48.4) | 1.43 (0.84-2.45) | 0.191 |  | 1.27 (0.73-2.22) | 0.405 |
| GG | 231 (17.8) | 1.97 (1.06-3.66) | 0.032 |  | 1.90 (0.99-3.64) | 0.054 |
| **rs698761** |  |  |  |  |  |  |
| GG | 588 (45.4) | 1 (ref) |  |  | 1 (ref) |  |
| AG | 540 (41.7) | 0.99 (0.60-1.62) | 0.958 |  | 1.63 (0.78-3.39) | 0.192 |
| AA | 168 (13.0) | **1.92 (1.07-3.44)** | **0.028** |  | **3.48 (1.45-8.33)** | **0.005** |
| rs3132555 |  |  |  |  |  |  |
| CC | 1013 (78.6) | 1 (ref) |  |  | 1 (ref) |  |
| CG | 85 (6.6) | 1.61 (0.77-3.37) | 0.204 |  | 1.62 (0.77-3.42) | 0.206 |
| GG | 190 (14.8) | 0.84 (0.43-1.63) | 0.599 |  | 0.86 (0.42-1.75) | 0.676 |
| rs1058267 |  |  |  |  |  |  |
| GG | 359 (27.7) | 1 (ref) |  |  | 1 (ref) |  |
| AG | 652 (50.3) | 1.02 (0.61-1.71) | 0.945 |  | 0.92 (0.53-1.57) | 0.746 |
| AA | 284 (21.9) | 0.94 (0.5-1.78) | 0.853 |  | 0.97 (0.51-1.85) | 0.915 |
| **rs8602** |  |  |  |  |  |  |
| CC | 656 (50.6) | 1 (ref) |  |  | 1 (ref) |  |
| AC | 518 (39.9) | 1.38 (0.85-2.23) | 0.189 |  | 1.45 (0.73-2.88) | 0.293 |
| AA | 123 (9.5) | **2.27 (1.19-4.32)** | **0.013** |  | **2.63 (1.04-6.65)** | **0.041** |
| rs12191285 |  |  |  |  |  |  |
| AA | 549 (42.7) | 1 (ref) |  |  | 1 (ref) |  |
| AG | 570 (44.3) | 1.29 (0.81-2.05) | 0.283 |  | 1.25 (0.76-2.05) | 0.372 |
| GG | 168 (13.1) | 0.71 (0.31-1.61) | 0.413 |  | 0.81 (0.35-1.86) | 0.617 |
| rs9614613 |  |  |  |  |  |  |
| CC | 598 (46.2) | 1 (ref) |  |  | 1 (ref) |  |
| CG | 570 (44.0) | 1.06 (0.66-1.70) | 0.799 |  | 0.99 (0.60-1.63) | 0.952 |
| GG | 127 (9.8) | 1.42 (0.7-2.88) | 0.329 |  | 1.50 (0.73-3.08) | 0.269 |
| rs2530310 |  |  |  |  |  |  |
| AA | 536 (41.4) | 1 (ref) |  |  | 1 (ref) |  |
| AG | 593 (45.8) | 1.25 (0.77-2.02) | 0.374 |  | 1.36 (0.82-2.25) | 0.240 |
| GG | 166 (12.8) | 1.48 (0.77-2.84) | 0.245 |  | 1.49 (0.73-3.03) | 0.273 |
| rs233113 |  |  |  |  |  |  |
| TT | 715 (55.2) | 1 (ref) |  |  | 1 (ref) |  |
| AT | 490 (37.8) | 1.34 (0.84-2.14) | 0.217 |  | 1.58 (0.97-2.56) | 0.066 |
| AA | 90 (6.9) | 1.81 (0.84-3.88) | 0.130 |  | 1.42 (0.55-3.65) | 0.474 |
| rs11920311 |  |  |  |  |  |  |
| GG | 746 (57.5) | 1 (ref) |  |  | 1 (ref) |  |
| AG | 471 (36.3) | 0.86 (0.53-1.37) | 0.520 |  | 0.93 (0.57-1.53) | 0.776 |
| AA | 80 (6.2) | 1.05 (0.42-2.63) | 0.926 |  | 1.19 (0.47-3.04) | 0.713 |
| rs8752 |  |  |  |  |  |  |
| GG | 547 (42.2) | 1 (ref) |  |  | 1 (ref) |  |
| AG | 613 (47.3) | 0.72 (0.45-1.13) | 0.153 |  | 0.72 (0.44-1.17) | 0.187 |
| AA | 135 (10.4) | 0.55 (0.24-1.30) | 0.176 |  | 0.50 (0.19-1.27) | 0.144 |
| rs1059829 |  |  |  |  |  |  |
| GG | 498 (38.4) | 1 (ref) |  |  | 1 (ref) |  |
| AG | 628 (48.4) | 1.09 (0.68-1.75) | 0.734 |  | 0.98 (0.60-1.60) | 0.926 |
| AA | 171 (13.2) | 1.01 (0.49-2.06) | 0.987 |  | 0.85 (0.40-1.81) | 0.671 |
| rs2971877 |  |  |  |  |  |  |
| GG | 415 (32.0) | 1 (ref) |  |  | 1 (ref) |  |
| AG | 611 (47.2) | 0.75 (0.46-1.23) | 0.258 |  | 0.77 (0.46-1.29) | 0.321 |
| AA | 269 (20.8) | 0.91 (0.50-1.64) | 0.746 |  | 0.74 (0.39-1.43) | 0.371 |
| rs1051434 |  |  |  |  |  |  |
| AA | 519 (40.0) | 1 (ref) |  |  | 1 (ref) |  |
| AG | 625 (48.2) | 0.81 (0.51-1.29) | 0.374 |  | 0.93 (0.57-1.51) | 0.755 |
| GG | 152 (11.7) | 0.82 (0.40-1.71) | 0.601 |  | 0.85 (0.39-1.86) | 0.678 |
| rs6480718 |  |  |  |  |  |  |
| CC | 474 (36.6) | 1 (ref) |  |  | 1 (ref) |  |
| CG | 641 (49.5) | 1.50 (0.92-2.45) | 0.105 |  | 1.89 (1.10-3.23) | 0.021 |
| GG | 181 (14.0) | 0.99 (0.46-2.11) | 0.971 |  | 1.22 (0.55-2.69) | 0.626 |
| rs1194 |  |  |  |  |  |  |
| GG | 597 (46.1) | 1 (ref) |  |  | 1 (ref) |  |
| AG | 575 (44.4) | 0.99 (0.62-1.57) | 0.958 |  | 0.84 (0.51-1.38) | 0.489 |
| AA | 123 (9.5) | 1.09 (0.51-2.35) | 0.821 |  | 1.26 (0.58-2.74) | 0.558 |
| rs7566 |  |  |  |  |  |  |
| GG | 722 (55.7) | 1 (ref) |  |  | 1 (ref) |  |
| AG | 473 (36.5) | 0.96 (0.60-1.53) | 0.856 |  | 0.90 (0.55-1.49) | 0.686 |
| AA | 101 (7.8) | 1.11 (0.50-2.45) | 0.805 |  | 1.01 (0.43-2.39) | 0.985 |
| rs2235882 |  |  |  |  |  |  |
| GG | 820 (63.3) | 1 (ref) |  |  | 1 (ref) |  |
| AG | 420 (32.4) | 1.17 (0.73-1.87) | 0.519 |  | 1.17 (0.71-1.93) | 0.548 |
| AA | 55 (4.2) | 0.87 (0.27-2.79) | 0.811 |  | 0.75 (0.23-2.43) | 0.626 |
| rs17765013 |  |  |  |  |  |  |
| AA | 1256 (96.8) | 1 (ref) |  |  | 1 (ref) |  |
| AG | 41 (3.2) | 1.35 (0.43-4.28) | 0.609 |  | 1.10 (0.27-4.54) | 0.897 |
| rs887842 |  |  |  |  |  |  |
| CC | 386 (29.8) | 1 (ref) |  |  | 1 (ref) |  |
| AC | 661 (51.0) | 0.77 (0.48-1.24) | 0.285 |  | 0.74 (0.45-1.22) | 0.235 |
| AA | 250 (19.3) | 0.65 (0.33-1.30) | 0.220 |  | 0.50 (0.23-1.11) | 0.087 |
| rs2550303 |  |  |  |  |  |  |
| AA | 451 (34.9) | 1 (ref) |  |  | 1 (ref) |  |
| AG | 642 (49.7) | 1.13 (0.69-1.85) | 0.625 |  | 1.14 (0.67-1.92) | 0.637 |
| GG | 199 (15.4) | 1.19 (0.61-2.31) | 0.614 |  | 1.20 (0.6-2.43) | 0.605 |
| rs963917 |  |  |  |  |  |  |
| AA | 569 (44.1) | 1 (ref) |  |  | 1 (ref) |  |
| AG | 476 (36.9) | 1.31 (0.80-2.17) | 0.287 |  | 1.60 (0.95-2.71) | 0.079 |
| GG | 245 (19.0) | 1.45 (0.81-2.58) | 0.210 |  | 1.45 (0.78-2.72) | 0.244 |
| rs963918 |  |  |  |  |  |  |
| AA | 752 (58.0) | 1 (ref) |  |  | 1 (ref) |  |
| AG | 446 (34.4) | 0.91 (0.56-1.48) | 0.713 |  | 1.03 (0.62-1.69) | 0.920 |
| GG | 99 (7.6) | 1.31 (0.62-2.78) | 0.481 |  | 0.86 (0.34-2.18) | 0.745 |
| rs3740237 |  |  |  |  |  |  |
| GG | 966 (74.5) | 1 (ref) |  |  | 1 (ref) |  |
| CG | 310 (23.9) | 0.80 (0.46-1.38) | 0.415 |  | 0.87 (0.50-1.53) | 0.631 |
| CC | 20 (1.5) | 1.66 (0.41-6.78) | 0.483 |  | 2.11 (0.50-8.93) | 0.311 |
| rs6566883 |  |  |  |  |  |  |
| AA | 560 (43.2) | 1 (ref) |  |  | 1 (ref) |  |
| AG | 569 (43.9) | 1.14 (0.71-1.82) | 0.587 |  | 1.11 (0.67-1.82) | 0.695 |
| GG | 168 (13.0) | 1.08 (0.53-2.20) | 0.828 |  | 1.30 (0.63-2.67) | 0.484 |
| rs9332 |  |  |  |  |  |  |
| GG | 922 (71.1) | 1 (ref) |  |  | 1 (ref) |  |
| AG | 347 (26.8) | 0.96 (0.58-1.59) | 0.877 |  | 0.95 (0.56-1.64) | 0.862 |
| AA | 28 (2.2) | 2.56 (0.93-7.07) | 0.070 |  | 2.65 (0.93-7.51) | 0.068 |
| rs8336 |  |  |  |  |  |  |
| GG | 611 (47.1) | 1 (ref) |  |  | 1 (ref) |  |
| AG | 578 (44.6) | 0.87 (0.55-1.39) | 0.568 |  | 0.89 (0.55-1.45) | 0.640 |
| AA | 108 (8.3) | 0.96 (0.43-2.15) | 0.925 |  | 1.02 (0.45-2.31) | 0.961 |
| rs4447076 |  |  |  |  |  |  |
| AA | 834 (64.3) | 1 (ref) |  |  | 1 (ref) |  |
| AG | 400 (30.8) | 0.63 (0.37-1.06) | 0.083 |  | 0.70 (0.40-1.21) | 0.197 |
| GG | 63 (4.9) | 0.79 (0.29-2.17) | 0.646 |  | 0.87 (0.31-2.44) | 0.792 |
| rs3774729 |  |  |  |  |  |  |
| AA | 358 (27.7) | 1 (ref) |  |  | 1 (ref) |  |
| AG | 621 (48.0) | 1.25 (0.72-2.17) | 0.421 |  | 1.28 (0.70-2.31) | 0.421 |
| GG | 315 (24.3) | 1.30 (0.70-2.40) | 0.403 |  | 1.43 (0.74-2.75) | 0.284 |
| rs7359387 |  |  |  |  |  |  |
| AA | 528 (40.8) | 1 (ref) |  |  | 1 (ref) |  |
| AC | 613 (47.3) | 0.89 (0.55-1.42) | 0.612 |  | 0.92 (0.56-1.53) | 0.751 |
| CC | 154 (11.9) | 1.10 (0.56-2.17) | 0.787 |  | 1.27 (0.63-2.55) | 0.503 |
| rs3809401 |  |  |  |  |  |  |
| GG | 407 (31.4) | 1 (ref) |  |  | 1 (ref) |  |
| AG | 649 (50.1) | 0.86 (0.52-1.41) | 0.542 |  | 0.95 (0.56-1.62) | 0.844 |
| AA | 240 (18.5) | 0.96 (0.51-1.79) | 0.894 |  | 1.17 (0.60-2.28) | 0.641 |
| rs14240 |  |  |  |  |  |  |
| GG | 398 (30.7) | 1 (ref) |  |  | 1 (ref) |  |
| AG | 628 (48.5) | 1.17 (0.69-1.98) | 0.562 |  | 1.14 (0.66-1.97) | 0.640 |
| AA | 270 (20.8) | 1.36 (0.74-2.48) | 0.326 |  | 1.23 (0.65-2.35) | 0.520 |
| rs2466551 |  |  |  |  |  |  |
| AA | 530 (40.9) | 1 (ref) |  |  | 1 (ref) |  |
| AC | 591 (45.6) | 1.03 (0.66-1.62) | 0.897 |  | 1.05 (0.65-1.71) | 0.833 |
| CC | 174 (13.4) | 0.37 (0.13-1.04) | 0.059 |  | 0.42 (0.15-1.19) | 0.103 |
| rs16974880 |  |  |  |  |  |  |
| AA | 657 (50.7) | 1 (ref) |  |  | 1 (ref) |  |
| AC | 554 (42.7) | 0.98 (0.63-1.53) | 0.926 |  | 1.03 (0.64-1.64) | 0.920 |
| CC | 85 (6.6) | 0.54 (0.17-1.74) | 0.302 |  | 0.58 (0.18-1.89) | 0.367 |
| rs2659582 |  |  |  |  |  |  |
| AA | 478 (36.9) | 1 (ref) |  |  | 1 (ref) |  |
| AG | 617 (47.6) | 1.20 (0.74-1.95) | 0.456 |  | 1.18 (0.71-1.97) | 0.517 |
| GG | 200 (15.4) | 1.02 (0.50-2.05) | 0.964 |  | 0.98 (0.47-2.06) | 0.954 |
| **rs10878441** |  |  |  |  |  |  |
| AA | 476 (36.7) | 1 (ref) |  |  | 1 (ref) |  |
| AC | 610 (47.0) | 1.00 (0.58-1.70) | 0.986 |  | 1.09 (0.52-2.29) | 0.811 |
| CC | 211 (16.3) | **2.63 (1.51-4.58)** | **0.001** |  | **2.46 (1.07-5.68)** | **0.035** |
| rs701848 |  |  |  |  |  |  |
| AA | 397 (30.6) | 1 (ref) |  |  | 1 (ref) |  |
| AG | 653 (50.3) | 1.06 (0.64-1.75) | 0.828 |  | 1.13 (0.66-1.91) | 0.659 |
| GG | 247 (19.0) | 1.00 (0.52-1.94) | 0.991 |  | 0.94 (0.46-1.93) | 0.873 |
| rs1049269 |  |  |  |  |  |  |
| GG | 329 (25.4) | 1 (ref) |  |  | 1 (ref) |  |
| AG | 644 (49.8) | 1.35 (0.78-2.33) | 0.277 |  | 1.49 (0.83-2.68) | 0.178 |
| AA | 321 (24.8) | 0.89 (0.45-1.74) | 0.723 |  | 0.98 (0.48-1.99) | 0.950 |
| rs1049684 |  |  |  |  |  |  |
| GG | 397 (30.7) | 1 (ref) |  |  | 1 (ref) |  |
| AG | 631 (48.7) | 0.94 (0.58-1.53) | 0.804 |  | 0.87 (0.52-1.46) | 0.608 |
| AA | 267 (20.6) | 0.59 (0.29-1.19) | 0.140 |  | 0.65 (0.31-1.33) | 0.238 |
| rs2497 |  |  |  |  |  |  |
| GG | 737 (57.2) | 1 (ref) |  |  | 1 (ref) |  |
| AG | 551 (42.8) | 1.33 (0.84-2.11) | 0.220 |  | 0.69 (0.43-1.13) | 0.142 |
| **rs10318** |  |  |  |  |  |  |
| AA | 351 (27.1) | 1 (ref) |  |  | 1 (ref) |  |
| AG | 660 (51.0) | 0.75 (0.47-1.21) | 0.242 |  | 0.47 (0.24-0.94) | 0.033 |
| GG | 283 (21.9) | **0.37 (0.18-0.78)** | **0.009** |  | **0.32 (0.13-0.80)** | **0.015** |
| rs2971879 |  |  |  |  |  |  |
| GG | 392 (30.3) | 1 (ref) |  |  | 1 (ref) |  |
| AG | 641 (49.5) | 0.87 (0.53-1.43) | 0.578 |  | 0.99 (0.58-1.70) | 0.971 |
| AA | 262 (20.2) | 0.81 (0.43-1.53) | 0.514 |  | 0.91 (0.47-1.80) | 0.795 |
| rs1595066 |  |  |  |  |  |  |
| GG | 571 (44.1) | 1 (ref) |  |  | 1 (ref) |  |
| AG | 566 (43.7) | 1.15 (0.72-1.82) | 0.560 |  | 1.17 (0.71-1.91) | 0.538 |
| AA | 158 (12.2) | 0.85 (0.39-1.84) | 0.682 |  | 1.02 (0.47-2.25) | 0.954 |
| rs786906 |  |  |  |  |  |  |
| GG | 334 (25.8) | 1 (ref) |  |  | 1 (ref) |  |
| AG | 646 (49.9) | 1.26 (0.74-2.15) | 0.399 |  | 1.10 (0.62-1.92) | 0.752 |
| AA | 314 (24.3) | 0.78 (0.39-1.55) | 0.470 |  | 0.74 (0.36-1.53) | 0.418 |
| rs2390606 |  |  |  |  |  |  |
| AA | 385 (29.8) | 1 (ref) | 0.168 |  | 1 (ref) |  |
| AC | 658 (50.9) | 1.56 (0.91-2.67) | 0.108 |  | 1.48 (0.84-2.60) | 0.176 |
| CC | 251 (19.4) | 1.02 (0.49-2.11) | 0.968 |  | 0.95 (0.43-2.08) | 0.895 |
| rs3764062 |  |  |  |  |  |  |
| AA | 650 (50.1) | 1 (ref) |  |  | 1 (ref) |  |
| AG | 546 (42.1) | 1.36 (0.86-2.13) | 0.187 |  | 1.45 (0.9-2.34) | 0.128 |
| GG | 101 (7.8) | 0.78 (0.28-2.20) | 0.641 |  | 0.9 (0.32-2.59) | 0.852 |
| rs3218210 |  |  |  |  |  |  |
| TT | 1292 (100.0) | - | - |  | - | - |
| rs3745550 |  |  |  |  |  |  |
| GG | 648 (50.0) | 1 (ref) |  |  | 1 (ref) |  |
| AG | 536 (41.3) | 1.18 (0.74-1.88) | 0.497 |  | 1.18 (0.72-1.92) | 0.508 |
| AA | 113 (8.7) | 1.79 (0.89-3.61) | 0.103 |  | 1.59 (0.73-3.47) | 0.245 |
| rs10510454 |  |  |  |  |  |  |
| GG | 324 (25.0) | 1 (ref) |  |  | 1 (ref) |  |
| AG | 660 (50.9) | 1.16 (0.65-2.05) | 0.620 |  | 1.46 (0.79-2.71) | 0.231 |
| AA | 313 (24.1) | 1.61 (0.87-2.98) | 0.130 |  | 1.81 (0.91-3.6) | 0.093 |
| rs2377028 |  |  |  |  |  |  |
| CC | 741 (57.2) | 1 (ref) |  |  | 1 (ref) |  |
| CG | 461 (35.6) | 0.93 (0.57-1.49) | 0.749 |  | 1.00 (0.61-1.66) | 0.990 |
| GG | 94 (7.3) | 1.47 (0.72-3.01) | 0.292 |  | 1.63 (0.75-3.54) | 0.216 |
| rs10888486 |  |  |  |  |  |  |
| GG | 411 (31.7) | 1 (ref) |  |  | 1 (ref) |  |
| AG | 635 (49.0) | 0.90 (0.56-1.45) | 0.664 |  | 0.75 (0.45-1.24) | 0.260 |
| AA | 250 (19.3) | 0.59 (0.29-1.18) | 0.135 |  | 0.62 (0.30-1.25) | 0.182 |
| rs2292822 |  |  |  |  |  |  |
| GG | 739 (57.2) | 1 (ref) |  |  | 1 (ref) |  |
| CG | 362 (28.0) | 1.07 (0.65-1.77) | 0.795 |  | 1.04 (0.61-1.77) | 0.879 |
| CC | 190 (14.7) | 1.01 (0.52-1.96) | 0.967 |  | 1.15 (0.57-2.32) | 0.688 |
| rs1062679 |  |  |  |  |  |  |
| GG | 598 (46.2) | 1 (ref) |  |  | 1 (ref) |  |
| CG | 564 (43.6) | 0.59 (0.36-0.97) | 0.038 |  | 0.54 (0.32-0.92) | 0.022 |
| CC | 132 (10.2) | 1.18 (0.62-2.25) | 0.609 |  | 1.04 (0.53-2.07) | 0.908 |
| rs3206635 |  |  |  |  |  |  |
| GG | 456 (35.2) | 1 (ref) |  |  | 1 (ref) |  |
| AG | 618 (47.6) | 1.15 (0.69-1.90) | 0.595 |  | 1.18 (0.70-2.01) | 0.538 |
| AA | 223 (17.2) | 1.39 (0.75-2.58) | 0.294 |  | 1.46 (0.75-2.81) | 0.264 |
| rs2941479 |  |  |  |  |  |  |
| CC | 516 (39.8) | 1 (ref) |  |  | 1 (ref) |  |
| AC | 601 (46.3) | 1.03 (0.64-1.66) | 0.907 |  | 0.98 (0.59-1.63) | 0.939 |
| AA | 180 (13.9) | 1.13 (0.58-2.21) | 0.714 |  | 1.13 (0.56-2.29) | 0.728 |
| rs715020 |  |  |  |  |  |  |
| GG | 708 (54.6) | 1 (ref) |  |  | 1 (ref) |  |
| AG | 500 (38.6) | 0.84 (0.53-1.35) | 0.476 |  | 0.90 (0.55-1.46) | 0.658 |
| AA | 88 (6.8) | 1.01 (0.43-2.37) | 0.978 |  | 0.90 (0.35-2.28) | 0.816 |
| rs7132908 |  |  |  |  |  |  |
| GG | 752 (58.1) | 1 (ref) |  |  | 1 (ref) |  |
| AG | 462 (35.7) | 1.12 (0.71-1.76) | 0.635 |  | 1.30 (0.81-2.10) | 0.283 |
| AA | 81 (6.3) | 0.59 (0.18-1.90) | 0.378 |  | 0.74 (0.22-2.41) | 0.611 |
| rs1180342 |  |  |  |  |  |  |
| AA | 394 (30.4) | 1 (ref) |  |  | 1 (ref) |  |
| AC | 673 (52.0) | 0.87 (0.54-1.42) | 0.585 |  | 0.82 (0.49-1.39) | 0.465 |
| CC | 227 (17.5) | 0.61 (0.30-1.26) | 0.181 |  | 0.64 (0.30-1.35) | 0.241 |
| rs2075802 |  |  |  |  |  |  |
| AA | 585 (45.1) | 1 (ref) |  |  | 1 (ref) |  |
| AC | 584 (45.0) | 1.12 (0.70-1.80) | 0.633 |  | 1.22 (0.74-2.01) | 0.433 |
| CC | 128 (9.9) | 1.59 (0.81-3.15) | 0.182 |  | 1.54 (0.73-3.26) | 0.261 |
| rs13734 |  |  |  |  |  |  |
| AA | 350 (27.0) | 1 (ref) |  |  | 1 (ref) |  |
| AG | 640 (49.4) | 0.80 (0.48-1.32) | 0.381 |  | 0.86 (0.51-1.45) | 0.562 |
| GG | 305 (23.6) | 0.80 (0.43-1.48) | 0.474 |  | 0.70 (0.35-1.39) | 0.309 |
| rs1044158 |  |  |  |  |  |  |
| GG | 559 (43.3) | 1 (ref) |  |  | 1 (ref) |  |
| AG | 555 (43.0) | 1.04 (0.66-1.63) | 0.876 |  | 1.02 (0.63-1.65) | 0.937 |
| AA | 178 (13.8) | 0.41 (0.16-1.03) | 0.058 |  | 0.36 (0.13-1.01) | 0.052 |
| rs6128327 |  |  |  |  |  |  |
| AA | 673 (52.0) | 1 (ref) |  |  | 1 (ref) |  |
| AG | 510 (39.4) | 0.73 (0.46-1.17) | 0.191 |  | 0.80 (0.49-1.31) | 0.379 |
| GG | 110 (8.5) | 0.50 (0.18-1.38) | 0.182 |  | 0.44 (0.14-1.43) | 0.172 |
| rs1051148 |  |  |  |  |  |  |
| CC | 383 (29.6) | 1 (ref) |  |  | 1 (ref) |  |
| AC | 656 (50.6) | 1.36 (0.79-2.33) | 0.271 |  | 1.52 (0.84-2.74) | 0.162 |
| AA | 257 (19.8) | 1.48 (0.78-2.79) | 0.229 |  | 1.84 (0.94-3.61) | 0.075 |
| rs4796030 |  |  |  |  |  |  |
| AA | 418 (32.2) | 1 (ref) |  |  | 1 (ref) |  |
| AC | 647 (49.9) | 0.92 (0.55-1.54) | 0.748 |  | 0.90 (0.53-1.56) | 0.715 |
| CC | 232 (17.9) | 1.46 (0.81-2.62) | 0.211 |  | 1.38 (0.74-2.57) | 0.309 |
| rs7221 |  |  |  |  |  |  |
| AA | 605 (51.0) | 1 (ref) |  |  | 1 (ref) |  |
| AG | 477 (40.2) | 0.98 (0.59-1.62) | 0.933 |  | 1.14 (0.67-1.93) | 0.632 |
| GG | 105 (8.8) | 2.06 (1.10-3.88) | 0.025 |  | 2.39 (1.20-4.72) | 0.013 |
| rs1043881 |  |  |  |  |  |  |
| GG | 597 (46.1) | 1 (ref) |  |  | 1 (ref) |  |
| AG | 552 (42.6) | 0.74 (0.46-1.20) | 0.223 |  | 0.88 (0.53-1.47) | 0.633 |
| AA | 147 (11.3) | 1.27 (0.67-2.42) | 0.466 |  | 1.57 (0.80-3.06) | 0.187 |
| rs551517 |  |  |  |  |  |  |
| GG | 697 (53.7) | 1 (ref) |  |  | 1 (ref) |  |
| AG | 502 (38.7) | 1.05 (0.66-1.67) | 0.837 |  | 1.17 (0.72-1.91) | 0.534 |
| AA | 98 (7.6) | 1.22 (0.55-2.71) | 0.631 |  | 1.38 (0.61-3.11) | 0.440 |
| rs2255090 |  |  |  |  |  |  |
| GG | 339 (26.2) | 1 (ref) |  |  | 1 (ref) |  |
| AG | 674 (52.1) | 1.00 (0.58-1.73) | 0.993 |  | 0.98 (0.55-1.75) | 0.948 |
| AA | 281 (21.7) | 1.26 (0.67-2.35) | 0.479 |  | 1.33 (0.69-2.58) | 0.397 |
| rs9731 |  |  |  |  |  |  |
| GG | 566 (43.7) | 1 (ref) |  |  | 1 (ref) |  |
| AG | 575 (44.4) | 1.02 (0.62-1.65) | 0.950 |  | 1.05 (0.63-1.75) | 0.847 |
| AA | 153 (11.8) | 1.65 (0.89-3.05) | 0.109 |  | 1.54 (0.80-2.98) | 0.201 |
| rs3757261 |  |  |  |  |  |  |
| GG | 953 (74.5) | 1 (ref) |  |  | 1 (ref) |  |
| AG | 206 (16.1) | 0.89 (0.48-1.65) | 0.707 |  | 0.99 (0.53-1.85) | 0.961 |
| AA | 120 (9.4) | 0.55 (0.20-1.50) | 0.243 |  | 0.45 (0.14-1.43) | 0.176 |
| rs11724758 |  |  |  |  |  |  |
| GG | 464 (35.8) | 1 (ref) |  |  | 1 (ref) |  |
| AG | 621 (47.9) | 1.45 (0.88-2.38) | 0.144 |  | 1.56 (0.92-2.65) | 0.098 |
| AA | 211 (16.3) | 1.17 (0.59-2.34) | 0.656 |  | 1.34 (0.65-2.80) | 0.428 |
| rs1047499 |  |  |  |  |  |  |
| AA | 376 (29.1) | 1 (ref) |  |  | 1 (ref) |  |
| AG | 633 (48.9) | 0.86 (0.51-1.44) | 0.561 |  | 1.00 (0.57-1.75) | 0.998 |
| GG | 285 (22.0) | 0.94 (0.51-1.73) | 0.834 |  | 1.07 (0.56-2.05) | 0.850 |
| rs2069086 |  |  |  |  |  |  |
| AA | 782 (60.3) | 1 (ref) |  |  | 1 (ref) |  |
| AC | 457 (35.3) | 1.29 (0.82-2.04) | 0.279 |  | 1.25 (0.77-2.03) | 0.366 |
| CC | 57 (4.4) | 1.53 (0.60-3.85) | 0.372 |  | 1.64 (0.64-4.23) | 0.304 |
| rs4016 |  |  |  |  |  |  |
| TT | 356 (27.4) | 1 (ref) |  |  | 1 (ref) |  |
| AT | 641 (49.4) | 0.71 (0.43-1.18) | 0.186 |  | 0.75 (0.43-1.29) | 0.292 |
| AA | 300 (23.1) | 0.90 (0.50-1.63) | 0.728 |  | 0.95 (0.51-1.78) | 0.876 |
| rs1050488 |  |  |  |  |  |  |
| AA | 629 (48.5) | 1 (ref) |  |  | 1 (ref) |  |
| AG | 563 (43.4) | 0.94 (0.60-1.49) | 0.798 |  | 1.01 (0.63-1.63) | 0.968 |
| GG | 104 (8.0) | 0.65 (0.26-1.63) | 0.354 |  | 0.56 (0.20-1.59) | 0.278 |
| rs12913 |  |  |  |  |  |  |
| GG | 432 (33.4) | 1 (ref) |  |  | 1 (ref) |  |
| AG | 609 (47.1) | 1.74 (1.02-2.98) | 0.042 |  | 1.71 (0.98-2.99) | 0.061 |
| AA | 253 (19.6) | 1.46 (0.75-2.83) | 0.270 |  | 1.44 (0.71-2.90) | 0.310 |
| rs13429321 |  |  |  |  |  |  |
| TT | 520 (40.2) | 1 (ref) |  |  | 1 (ref) |  |
| AT | 614 (47.4) | 1.48 (0.90-2.42) | 0.121 |  | 1.34 (0.80-2.24) | 0.261 |
| AA | 161 (12.4) | 1.64 (0.82-3.27) | 0.159 |  | 1.29 (0.61-2.72) | 0.501 |
| rs17053540 |  |  |  |  |  |  |
| GG | 971 (74.9) | 1 (ref) |  |  | 1 (ref) |  |
| AG | 305 (23.5) | 0.99 (0.59-1.66) | 0.959 |  | 1.12 (0.65-1.92) | 0.691 |
| AA | 21 (1.6) | 2.71 (0.85-8.65) | 0.092 |  | 4.44 (1.34-14.71) | 0.015 |
| rs2078864 |  |  |  |  |  |  |
| GG | 416 (32.3) | 1 (ref) |  |  | 1 (ref) |  |
| AG | 640 (49.7) | 1.06 (0.65-1.73) | 0.823 |  | 1.00 (0.59-1.70) | 0.994 |
| AA | 232 (18.0) | 0.93 (0.48-1.80) | 0.823 |  | 1.05 (0.53-2.09) | 0.896 |
| rs3087960 |  |  |  |  |  |  |
| GG | 587 (45.3) | 1 (ref) |  |  | 1 (ref) |  |
| CG | 573 (44.2) | 0.97 (0.60-1.57) | 0.902 |  | 1.04 (0.62-1.73) | 0.881 |
| CC | 136 (10.5) | 1.75 (0.93-3.32) | 0.085 |  | 2.14 (1.11-4.15) | 0.024 |
| **rs10075853** |  |  |  |  |  |  |
| AA | 802 (61.8) | 1 (ref) |  |  | 1 (ref) |  |
| AG | 412 (31.8) | 1.32 (0.82-2.14) | 0.256 |  | 1.22 (0.60-2.46) | 0.583 |
| GG | 83 (6.4) | **2.52 (1.30-4.91)** | **0.006** |  | **3.58 (1.26-10.14)** | **0.017** |
| rs10270308 |  |  |  |  |  |  |
| AA | 522 (40.2) | 1 (ref) |  |  | 1 (ref) |  |
| AG | 609 (47.0) | 1.01 (0.62-1.65) | 0.956 |  | 0.99 (0.59-1.66) | 0.982 |
| GG | 166 (12.8) | 1.68 (0.89-3.17) | 0.110 |  | 1.95 (1.01-3.77) | 0.047 |
| rs1059310 |  |  |  |  |  |  |
| GG | 595 (45.9) | 1 (ref) |  |  | 1 (ref) |  |
| AG | 564 (43.6) | 1.04 (0.65-1.67) | 0.867 |  | 0.92 (0.56-1.51) | 0.748 |
| AA | 136 (10.5) | 1.22 (0.62-2.40) | 0.572 |  | 0.87 (0.40-1.90) | 0.722 |
| rs1045004 |  |  |  |  |  |  |
| GG | 739 (57.0) | 1 (ref) |  |  | 1 (ref) |  |
| AG | 494 (38.1) | 1.19 (0.76-1.88) | 0.450 |  | 1.26 (0.78-2.03) | 0.349 |
| AA | 64 (4.9) | 1.29 (0.51-3.26) | 0.592 |  | 1.27 (0.45-3.61) | 0.649 |
| rs2664370 |  |  |  |  |  |  |
| AA | 479 (36.9) | 1 (ref) |  |  | 1 (ref) |  |
| AG | 615 (47.4) | 0.97 (0.61-1.56) | 0.907 |  | 0.88 (0.54-1.45) | 0.625 |
| GG | 203 (15.7) | 0.74 (0.36-1.50) | 0.396 |  | 0.72 (0.34-1.52) | 0.384 |
| rs3813034 |  |  |  |  |  |  |
| CC | 850 (65.5) | 1 (ref) |  |  | 1 (ref) |  |
| AC | 393 (30.3) | 0.93 (0.56-1.52) | 0.758 |  | 1.00 (0.59-1.68) | 0.987 |
| AA | 54 (4.2) | 1.59 (0.64-3.99) | 0.320 |  | 1.71 (0.68-4.34) | 0.257 |
| rs11173459 |  |  |  |  |  |  |
| AA | 523 (40.4) | 1 (ref) |  |  | 1 (ref) |  |
| AG | 598 (46.1) | 0.72 (0.45-1.16) | 0.177 |  | 0.65 (0.40-1.08) | 0.094 |
| GG | 175 (13.5) | 0.78 (0.39-1.57) | 0.489 |  | 0.74 (0.36-1.52) | 0.415 |
| rs608823 |  |  |  |  |  |  |
| GG | 426 (32.9) | 1 (ref) |  |  | 1 (ref) |  |
| AG | 603 (46.6) | 1.16 (0.70-1.92) | 0.577 |  | 1.19 (0.69-2.03) | 0.535 |
| AA | 264 (20.4) | 1.19 (0.63-2.24) | 0.589 |  | 1.38 (0.71-2.68) | 0.347 |
| rs13242090 |  |  |  |  |  |  |
| AA | 648 (50.0) | 1 (ref) |  |  | 1 (ref) |  |
| AG | 543 (41.9) | 1.05 (0.66-1.66) | 0.846 |  | 0.98 (0.6-1.61) | 0.945 |
| GG | 106 (8.2) | 1.11 (0.50-2.48) | 0.804 |  | 1.20 (0.53-2.74) | 0.658 |
| rs1130741 |  |  |  |  |  |  |
| AA | 764 (58.9) | 1 (ref) |  |  | 1 (ref) |  |
| AG | 452 (34.8) | 1.06 (0.67-1.70) | 0.795 |  | 1.11 (0.67-1.82) | 0.692 |
| GG | 81 (6.2) | 1.04 (0.42-2.63) | 0.928 |  | 1.22 (0.48-3.11) | 0.684 |
| rs17264436 |  |  |  |  |  |  |
| TT | 435 (33.6) | 1 (ref) |  |  | 1 (ref) |  |
| AT | 590 (45.5) | 1.30 (0.78-2.16) | 0.318 |  | 1.17 (0.68-2.00) | 0.567 |
| AA | 271 (20.9) | 1.09 (0.57-2.08) | 0.803 |  | 1.10 (0.56-2.16) | 0.793 |
| rs1325774 |  |  |  |  |  |  |
| AA | 996 (76.9) | 1 (ref) |  |  | 1 (ref) |  |
| AC | 272 (21.0) | 0.70 (0.38-1.29) | 0.255 |  | 0.62 (0.32-1.22) | 0.169 |
| CC | 28 (2.2) | 1.65 (0.52-5.26) | 0.396 |  | 1.84 (0.57-6.00) | 0.310 |
| rs1044123 |  |  |  |  |  |  |
| AA | 415 (32.0) | 1 (ref) |  |  | 1 (ref) |  |
| AG | 659 (50.8) | 1.02 (0.62-1.68) | 0.937 |  | 0.96 (0.56-1.63) | 0.870 |
| GG | 223 (17.2) | 1.18 (0.62-2.24) | 0.608 |  | 1.00 (0.50-2.01) | 0.992 |
| rs11538239 |  |  |  |  |  |  |
| GG | 472 (36.8) | 1 (ref) |  |  | 1 (ref) |  |
| AG | 594 (46.3) | 0.82 (0.50-1.36) | 0.445 |  | 0.86 (0.51-1.46) | 0.579 |
| AA | 218 (17.0) | 1.24 (0.68-2.25) | 0.482 |  | 1.18 (0.63-2.24) | 0.604 |
| rs10196 |  |  |  |  |  |  |
| AA | 515 (39.7) | 1 (ref) |  |  | 1 (ref) |  |
| AG | 617 (47.6) | 1.13 (0.69-1.83) | 0.635 |  | 1.22 (0.73-2.05) | 0.447 |
| GG | 164 (12.7) | 1.47 (0.76-2.84) | 0.250 |  | 1.40 (0.68-2.87) | 0.357 |
| rs1058747 |  |  |  |  |  |  |
| AA | 728 (56.2) | 1 (ref) |  |  | 1 (ref) |  |
| AG | 497 (38.3) | 1.04 (0.66-1.64) | 0.866 |  | 1.15 (0.71-1.86) | 0.576 |
| GG | 71 (5.5) | 0.89 (0.32-2.49) | 0.830 |  | 1.10 (0.39-3.12) | 0.855 |
| rs2241114 |  |  |  |  |  |  |
| GG | 351 (27.1) | 1 (ref) |  |  | 1 (ref) |  |
| CG | 643 (49.6) | 1.14 (0.67-1.92) | 0.636 |  | 1.14 (0.66-1.98) | 0.644 |
| CC | 302 (23.3) | 1.04 (0.55-1.94) | 0.915 |  | 0.91 (0.47-1.77) | 0.781 |
| rs3802703 |  |  |  |  |  |  |
| AA | 956 (73.8) | 1 (ref) |  |  | 1 (ref) |  |
| AG | 313 (24.2) | 0.36 (0.17-0.74) | 0.005 |  | 0.41 (0.19-0.85) | 0.017 |
| GG | 26 (2.0) | 1.09 (0.27-4.44) | 0.906 |  | 0.66 (0.09-4.86) | 0.685 |
| rs2280692 |  |  |  |  |  |  |
| GG | 638 (49.2) | 1 (ref) |  |  | 1 (ref) |  |
| AG | 554 (42.7) | 1.04 (0.67-1.63) | 0.849 |  | 1.02 (0.64-1.63) | 0.942 |
| AA | 105 (8.1) | 0.35 (0.08-1.43) | 0.144 |  | 0.32 (0.08-1.34) | 0.120 |
| rs7974459 |  |  |  |  |  |  |
| GG | 444 (34.3) | 1 (ref) |  |  | 1 (ref) |  |
| AG | 617 (47.7) | 0.96 (0.60-1.55) | 0.876 |  | 0.88 (0.54-1.44) | 0.607 |
| AA | 232 (17.9) | 0.79 (0.40-1.54) | 0.480 |  | 0.44 (0.19-1.01) | 0.053 |
| rs7655413 |  |  |  |  |  |  |
| AA | 412 (31.8) | 1 (ref) |  |  | 1 (ref) |  |
| AG | 657 (50.7) | 1.16 (0.70-1.95) | 0.564 |  | 1.30 (0.75-2.25) | 0.360 |
| GG | 227 (17.5) | 1.16 (0.60-2.23) | 0.666 |  | 1.38 (0.70-2.74) | 0.355 |
| rs10205020 |  |  |  |  |  |  |
| AA | 668 (51.5) | 1 (ref) |  |  | 1 (ref) |  |
| AG | 531 (41.0) | 1.12 (0.71-1.76) | 0.620 |  | 1.07 (0.66-1.72) | 0.789 |
| GG | 97 (7.5) | 0.50 (0.16-1.62) | 0.247 |  | 0.34 (0.08-1.40) | 0.134 |
| rs2654981 |  |  |  |  |  |  |
| GG | 462 (35.6) | 1 (ref) |  |  | 1 (ref) |  |
| CG | 619 (47.8) | 0.89 (0.56-1.41) | 0.617 |  | 0.93 (0.57-1.52) | 0.779 |
| CC | 215 (16.6) | 0.35 (0.15-0.84) | 0.018 |  | 0.38 (0.16-0.93) | 0.033 |
| rs9611591 |  |  |  |  |  |  |
| AA | 362 (27.9) | 1 (ref) |  |  | 1 (ref) |  |
| AG | 628 (48.4) | 1.06 (0.60-1.88) | 0.832 |  | 1.09 (0.59-2.00) | 0.791 |
| GG | 307 (23.7) | 1.06 (0.57-1.97) | 0.854 |  | 1.20 (0.62-2.32) | 0.582 |
| **rs8410** |  |  |  |  |  |  |
| GG | 599 (46.3) | 1 (ref) |  |  | 1 (ref) |  |
| AG | 532 (41.1) | 0.95 (0.58-1.56) | 0.840 |  | 1.63 (0.78-3.40) | 0.191 |
| AA | 163 (12.6) | **1.98 (1.11-3.54)** | **0.021** |  | **3.63 (1.52-8.71)** | **0.004** |
| rs3809724 |  |  |  |  |  |  |
| GG | 826 (63.7) | 1 (ref) |  |  | 1 (ref) |  |
| AG | 415 (32.0) | 0.74 (0.45-1.22) | 0.238 |  | 0.86 (0.51-1.44) | 0.564 |
| AA | 55 (4.2) | 1.23 (0.45-3.41) | 0.686 |  | 1.08 (0.33-3.54) | 0.895 |
| rs9556713 |  |  |  |  |  |  |
| AA | 844 (65.2) | 1 (ref) |  |  | 1 (ref) |  |
| AG | 401 (31.0) | 1.37 (0.87-2.16) | 0.177 |  | 1.34 (0.83-2.18) | 0.237 |
| GG | 49 (3.8) | 0.79 (0.19-3.25) | 0.742 |  | 0.97 (0.23-4.07) | 0.971 |
| rs1048906 |  |  |  |  |  |  |
| GG | 554 (42.7) | 1 (ref) |  |  | 1 (ref) |  |
| AG | 580 (44.7) | 1.11 (0.70-1.78) | 0.649 |  | 1.14 (0.70-1.86) | 0.589 |
| AA | 163 (12.6) | 0.90 (0.43-1.88) | 0.782 |  | 0.79 (0.34-1.83) | 0.586 |
| rs882869 |  |  |  |  |  |  |
| AA | 352 (27.2) | 1 (ref) |  |  | 1 (ref) |  |
| AG | 638 (49.3) | 1.63 (0.94-2.82) | 0.081 |  | 1.43 (0.80-2.53) | 0.225 |
| GG | 305 (23.6) | 0.77 (0.37-1.61) | 0.480 |  | 0.71 (0.33-1.53) | 0.378 |
| rs2071501 |  |  |  |  |  |  |
| AA | 504 (39.0) | 1 (ref) |  |  | 1 (ref) |  |
| AC | 596 (46.1) | 1.27 (0.78-2.05) | 0.338 |  | 1.18 (0.70-1.98) | 0.531 |
| CC | 192 (14.9) | 1.00 (0.50-2.02) | 0.997 |  | 1.16 (0.57-2.39) | 0.679 |
| rs7237996 |  |  |  |  |  |  |
| AA | 470 (36.3) | 1 (ref) |  |  | 1 (ref) |  |
| AG | 614 (47.4) | 1.14 (0.7-1.85) | 0.607 |  | 1.10 (0.66-1.85) | 0.715 |
| GG | 211 (16.3) | 1.17 (0.6-2.26) | 0.651 |  | 1.38 (0.70-2.72) | 0.353 |
| rs17063228 |  |  |  |  |  |  |
| AA | 1053 (81.2) | 1 (ref) |  |  | 1 (ref) |  |
| AT | 253 (19.5) | 1.11 (0.66-1.88) | 0.689 |  | 1.11 (0.64-1.92) | 0.710 |
| TT | 11 (0.8) | 0 (0-2.004E+194) | 0.962 |  | - | 0.977 |
| rs1044268 |  |  |  |  |  |  |
| AA | 415 (32.0) | 1 (ref) |  |  | 1 (ref) |  |
| AG | 642 (49.5) | 0.79 (0.48-1.29) | 0.342 |  | 0.76 (0.45-1.28) | 0.305 |
| GG | 239 (18.4) | 1.01 (0.55-1.87) | 0.966 |  | 1.05 (0.55-1.98) | 0.892 |
| rs3734201 |  |  |  |  |  |  |
| AA | 668 (51.6) | 1 (ref) |  |  | 1 (ref) |  |
| AG | 522 (40.3) | 1.59 (1.01-2.49) | 0.045 |  | 1.57 (0.97-2.52) | 0.065 |
| GG | 105 (8.1) | 0.80 (0.29-2.25) | 0.674 |  | 0.82 (0.29-2.31) | 0.700 |
| rs11844549 |  |  |  |  |  |  |
| AA | 736 (56.9) | 1 (ref) |  |  | 1 (ref) |  |
| AG | 483 (37.4) | 1.22 (0.78-1.91) | 0.390 |  | 0.98 (0.61-1.59) | 0.947 |
| GG | 74 (5.7) | 0.77 (0.24-2.49) | 0.664 |  | 0.75 (0.23-2.44) | 0.630 |
| rs13422 |  |  |  |  |  |  |
| CC | 574 (44.6) | 1 (ref) |  |  | 1 (ref) |  |
| AC | 565 (43.9) | 1.08 (0.69-1.70) | 0.741 |  | 1.14 (0.71-1.84) | 0.596 |
| AA | 149 (11.6) | 0.53 (0.21-1.36) | 0.189 |  | 0.47 (0.17-1.33) | 0.153 |
| rs854802 |  |  |  |  |  |  |
| AA | 583 (45.0) | 1 (ref) |  |  | 1 (ref) |  |
| AG | 572 (44.1) | 0.97 (0.61-1.54) | 0.895 |  | 0.96 (0.59-1.57) | 0.864 |
| GG | 141 (10.9) | 1.07 (0.51-2.22) | 0.860 |  | 1.38 (0.65-2.91) | 0.399 |
| rs6923492 |  |  |  |  |  |  |
| AA | 394 (30.5) | 1 (ref) |  |  | 1 (ref) |  |
| AG | 657 (50.8) | 0.80 (0.47-1.34) | 0.391 |  | 0.82 (0.47-1.42) | 0.478 |
| GG | 242 (18.7) | 1.35 (0.75-2.43) | 0.313 |  | 1.23 (0.65-2.33) | 0.533 |
| rs587404 |  |  |  |  |  |  |
| GG | 469 (36.2) | 1 (ref) |  |  | 1 (ref) |  |
| AG | 624 (48.2) | 1.06 (0.65-1.71) | 0.823 |  | 1.04 (0.63-1.73) | 0.867 |
| AA | 199 (15.4) | 1.11 (0.57-2.19) | 0.754 |  | 1.08 (0.52-2.26) | 0.838 |
| rs12677519 |  |  |  |  |  |  |
| GG | 451 (34.8) | 1 (ref) |  |  | 1 (ref) |  |
| AG | 622 (48.0) | 1.23 (0.73-2.06) | 0.436 |  | 1.16 (0.67-2.00) | 0.595 |
| AA | 222 (17.1) | 1.64 (0.88-3.04) | 0.117 |  | 1.65 (0.86-3.18) | 0.136 |
| rs2273847 |  |  |  |  |  |  |
| AA | 648 (50.0) | 1 (ref) |  |  | 1 (ref) |  |
| AG | 544 (42.0) | 1.44 (0.91-2.28) | 0.124 |  | 1.35 (0.83-2.19) | 0.223 |
| GG | 104 (8.0) | 1.30 (0.57-2.93) | 0.533 |  | 1.14 (0.47-2.76) | 0.780 |
| rs3825195 |  |  |  |  |  |  |
| GG | 339 (26.2) | 1 (ref) |  |  | 1 (ref) |  |
| AG | 637 (49.2) | 0.80 (0.47-1.37) | 0.420 |  | 0.82 (0.46-1.45) | 0.494 |
| AA | 320 (24.7) | 1.17 (0.66-2.07) | 0.601 |  | 1.26 (0.68-2.32) | 0.462 |
| rs1805100 |  |  |  |  |  |  |
| GG | 587 (45.3) | 1 (ref) |  |  | 1 (ref) |  |
| AG | 569 (43.9) | 1.05 (0.66-1.66) | 0.849 |  | 0.98 (0.60-1.60) | 0.939 |
| AA | 139 (10.7) | 0.96 (0.45-2.07) | 0.924 |  | 0.96 (0.42-2.20) | 0.931 |
| rs2506141 |  |  |  |  |  |  |
| GG | 819 (63.1) | 1 (ref) |  |  | 1 (ref) |  |
| AG | 417 (32.2) | 0.86 (0.53-1.40) | 0.546 |  | 0.85 (0.51-1.41) | 0.522 |
| AA | 61 (4.7) | 1.13 (0.41-3.12) | 0.815 |  | 1.01 (0.31-3.25) | 0.993 |
| rs3728 |  |  |  |  |  |  |
| CC | 444 (34.3) | 1 (ref) |  |  | 1 (ref) |  |
| AC | 620 (47.8) | 1.51 (0.89-2.55) | 0.123 |  | 1.36 (0.79-2.35) | 0.274 |
| AA | 232 (17.9) | 1.68 (0.88-3.18) | 0.114 |  | 1.67 (0.86-3.24) | 0.130 |
| rs11912802 |  |  |  |  |  |  |
| GG | 807 (62.3) | 1 (ref) |  |  | 1 (ref) |  |
| AG | 434 (33.5) | 0.95 (0.59-1.53) | 0.826 |  | 0.88 (0.53-1.47) | 0.635 |
| AA | 55 (4.2) | 1.46 (0.58-3.66) | 0.421 |  | 1.78 (0.70-4.57) | 0.229 |
| rs2942 |  |  |  |  |  |  |
| GG | 396 (30.6) | 1 (ref) |  |  | 1 (ref) |  |
| AG | 657 (50.7) | 0.77 (0.46-1.29) | 0.325 |  | 0.79 (0.46-1.36) | 0.401 |
| AA | 243 (18.8) | 1.30 (0.73-2.33) | 0.371 |  | 1.18 (0.63-2.23) | 0.610 |
| rs2272669 |  |  |  |  |  |  |
| GG | 589 (45.5) | 1 (ref) |  |  | 1 (ref) |  |
| AG | 567 (43.8) | 1.05 (0.66-1.67) | 0.834 |  | 0.98 (0.60-1.61) | 0.950 |
| AA | 138 (10.7) | 0.97 (0.45-2.09) | 0.944 |  | 0.98 (0.43-2.23) | 0.956 |
| rs16845990 |  |  |  |  |  |  |
| AA | 391 (30.1) | 1 (ref) |  |  | 1 (ref) |  |
| AG | 631 (48.7) | 1.19 (0.72-1.96) | 0.509 |  | 1.17 (0.69-2.00) | 0.554 |
| GG | 268 (20.7) | 0.83 (0.42-1.63) | 0.581 |  | 0.94 (0.47-1.89) | 0.860 |
| rs1059111 |  |  |  |  |  |  |
| TT | 565 (43.7) | 1 (ref) |  |  | 1 (ref) | 0.611 |
| AT | 577 (44.6) | 0.90 (0.56-1.45) | 0.670 |  | 1.11 (0.67-1.85) | 0.680 |
| AA | 151 (11.7) | 1.26 (0.65-2.41) | 0.496 |  | 1.41 (0.71-2.79) | 0.321 |
| rs1509617 |  |  |  |  |  |  |
| CC | 686 (53.1) | 1 (ref) |  |  | 1 (ref) |  |
| CG | 508 (39.3) | 1.63 (1.04-2.56) | 0.034 |  | 1.59 (0.99-2.57) | 0.055 |
| GG | 97 (7.5) | 0.91 (0.32-2.56) | 0.859 |  | 1.08 (0.38-3.09) | 0.891 |
| rs6958 |  |  |  |  |  |  |
| CC | 355 (27.4) | 1 (ref) |  |  | 1 (ref) |  |
| CG | 660 (50.9) | 0.83 (0.50-1.39) | 0.487 |  | 0.85 (0.49-1.46) | 0.547 |
| GG | 282 (21.7) | 0.92 (0.50-1.70) | 0.794 |  | 0.98 (0.52-1.87) | 0.957 |
| rs3733125 |  |  |  |  |  |  |
| GG | 775 (59.8) | 1 (ref) |  |  | 1 (ref) |  |
| AG | 455 (35.1) | 1.42 (0.90-2.24) | 0.132 |  | 1.33 (0.82-2.17) | 0.249 |
| AA | 66 (5.1) | 1.32 (0.52-3.33) | 0.560 |  | 1.58 (0.62-4.06) | 0.339 |
| rs1599796 |  |  |  |  |  |  |
| GG | 620 (48.0) | 1 (ref) |  |  | 1 (ref) |  |
| AG | 543 (42.0) | 1.01 (0.63-1.63) | 0.967 |  | 0.92 (0.55-1.54) | 0.747 |
| AA | 129 (10.0) | 1.75 (0.93-3.31) | 0.085 |  | 1.99 (1.02-3.89) | 0.043 |
| rs1065518 |  |  |  |  |  |  |
| TT | 555 (42.9) | 1 (ref) |  |  | 1 (ref) |  |
| AT | 603 (46.6) | 0.88 (0.56-1.38) | 0.571 |  | 0.82 (0.51-1.32) | 0.414 |
| AA | 137 (10.6) | 0.51 (0.20-1.31) | 0.162 |  | 0.42 (0.15-1.18) | 0.101 |
| rs8461 |  |  |  |  |  |  |
| GG | 560 (43.2) | 1 (ref) |  |  | 1 (ref) |  |
| AG | 586 (45.2) | 0.89 (0.56-1.41) | 0.617 |  | 1.06 (0.63-1.78) | 0.825 |
| AA | 150 (11.6) | 0.35 (0.15-0.84) | 0.018 |  | 1.97 (1.04-3.74) | 0.038 |
| rs1045832 |  |  |  |  |  |  |
| AA | 383 (29.7) | 1 (ref) |  |  | 1 (ref) |  |
| AG | 657 (50.9) | 1.35 (0.79-2.3) | 0.273 |  | 1.25 (0.72-2.16) | 0.433 |
| GG | 251 (19.4) | 1.10 (0.56-2.16) | 0.785 |  | 0.90 (0.44-1.84) | 0.776 |
| rs11108452 |  |  |  |  |  |  |
| AA | 666 (51.3) | 1 (ref) |  |  | 1 (ref) |  |
| AG | 529 (40.8) | 1.15 (0.73-1.79) | 0.549 |  | 1.12 (0.70-1.79) | 0.652 |
| GG | 102 (7.9) | 0.44 (0.14-1.43) | 0.171 |  | 0.43 (0.13-1.42) | 0.166 |
| rs10980234 |  |  |  |  |  |  |
| AA | 702 (54.1) | 1 (ref) |  |  | 1 (ref) |  |
| AG | 523 (40.3) | 1.47 (0.93-2.30) | 0.097 |  | 1.66 (1.03-2.68) | 0.037 |
| GG | 72 (5.6) | 1.06 (0.38-2.98) | 0.907 |  | 1.62 (0.57-4.63) | 0.369 |
| rs6845 |  |  |  |  |  |  |
| GG | 870 (67.1) | 1 (ref) |  |  | 1 (ref) |  |
| AG | 394 (30.4) | 1.33 (0.84-2.11) | 0.228 |  | 1.19 (0.72-1.96) | 0.500 |
| AA | 33 (2.5) | 1.59 (0.50-5.11) | 0.436 |  | 1.62 (0.49-5.34) | 0.427 |
| rs8031107 |  |  |  |  |  |  |
| GG | 347 (26.8) | 1 (ref) |  |  | 1 (ref) |  |
| AG | 616 (47.5) | 0.80 (0.48-1.32) | 0.379 |  | 0.83 (0.49-1.41) | 0.493 |
| AA | 333 (25.7) | 0.74 (0.40-1.36) | 0.327 |  | 0.67 (0.35-1.28) | 0.221 |
| rs2200285 |  |  |  |  |  |  |
| GG | 992 (77.9) | 1 (ref) |  |  | 1 (ref) |  |
| AG | 109 (8.6) | 0.72 (0.29-1.80) | 0.483 |  | 0.77 (0.31-1.94) | 0.582 |
| AA | 173 (13.6) | 1.07 (0.59-1.95) | 0.827 |  | 1.03 (0.55-1.94) | 0.926 |
| rs2307220 |  |  |  |  |  |  |
| AA | 551 (42.5) | 1 (ref) |  |  | 1 (ref) |  |
| AC | 571 (44.1) | 0.94 (0.59-1.50) | 0.801 |  | 0.90 (0.55-1.47) | 0.658 |
| CC | 173 (13.4) | 0.94 (0.47-1.90) | 0.868 |  | 0.92 (0.44-1.93) | 0.819 |
| rs6960867 |  |  |  |  |  |  |
| AA | 849 (65.5) | 1 (ref) |  |  | 1 (ref) |  |
| AG | 393 (30.3) | 0.74 (0.45-1.23) | 0.249 |  | 0.61 (0.35-1.08) | 0.088 |
| GG | 55 (4.2) | 0.78 (0.25-2.50) | 0.680 |  | 0.56 (0.13-2.30) | 0.418 |
| rs41739 |  |  |  |  |  |  |
| AA | 367 (28.3) | 1 (ref) |  |  | 1 (ref) |  |
| AG | 673 (52.0) | 0.83 (0.51-1.35) | 0.447 |  | 0.78 (0.46-1.30) | 0.333 |
| GG | 255 (19.7) | 0.65 (0.33-1.26) | 0.203 |  | 0.59 (0.29-1.17) | 0.131 |
| rs26317 |  |  |  |  |  |  |
| AA | 357 (27.6) | 1 (ref) |  |  | 1 (ref) |  |
| AG | 640 (49.4) | 1.58 (0.91-2.76) | 0.103 |  | 1.35 (0.76-2.39) | 0.302 |
| GG | 298 (23.0) | 1.04 (0.52-2.09) | 0.907 |  | 0.99 (0.48-2.06) | 0.980 |
| rs2018650 |  |  |  |  |  |  |
| AA | 721 (55.6) | 1 (ref) |  |  | 1 (ref) |  |
| AG | 499 (38.5) | 1.43 (0.91-2.23) | 0.119 |  | 1.36 (0.85-2.19) | 0.205 |
| GG | 76 (5.9) | 0.75 (0.23-2.43) | 0.629 |  | 0.75 (0.23-2.44) | 0.631 |
| rs2459965 |  |  |  |  |  |  |
| GG | 924 (71.5) | 1 (ref) |  |  | 1 (ref) |  |
| AG | 341 (26.4) | 1.15 (0.71-1.87) | 0.562 |  | 1.13 (0.68-1.88) | 0.642 |
| AA | 28 (2.2) | 1.04 (0.25-4.25) | 0.962 |  | 1.31 (0.31-5.50) | 0.712 |
| rs4902357 |  |  |  |  |  |  |
| GG | 497 (38.3) | 1 (ref) |  |  | 1 (ref) |  |
| CG | 630 (48.6) | 0.87 (0.55-1.39) | 0.567 |  | 1.02 (0.62-1.67) | 0.951 |
| CC | 169 (13.0) | 0.72 (0.35-1.50) | 0.381 |  | 0.92 (0.43-1.95) | 0.817 |
| rs2072693 |  |  |  |  |  |  |
| AA | 516 (40.0) | 1 (ref) |  |  | 1 (ref) |  |
| AC | 617 (47.8) | 1.56 (0.96-2.54) | 0.072 |  | 1.38 (0.83-2.30) | 0.218 |
| CC | 158 (12.2) | 0.99 (0.43-2.28) | 0.977 |  | 0.86 (0.35-2.12) | 0.742 |
| rs12107172 |  |  |  |  |  |  |
| AA | 876 (67.6) | 1 (ref) |  |  | 1 (ref) |  |
| AG | 374 (28.9) | 0.95 (0.58-1.56) | 0.851 |  | 0.97 (0.58-1.64) | 0.921 |
| GG | 55 (4.2) | 0.96 (0.30-3.06) | 0.940 |  | 1.21 (0.37-3.94) | 0.747 |
| rs485609 |  |  |  |  |  |  |
| CC | 434 (33.5) | 1 (ref) |  |  | 1 (ref) |  |
| CG | 645 (49.8) | 0.94 (0.57-1.57) | 0.818 |  | 0.83 (0.49-1.41) | 0.491 |
| GG | 215 (16.6) | 1.32 (0.73-2.41) | 0.362 |  | 1.02 (0.53-1.96) | 0.950 |
| rs4823006 |  |  |  |  |  |  |
| GG | 372 (28.7) | 1 (ref) |  |  | 1 (ref) |  |
| AG | 654 (50.5) | 1.1 (0.66-1.85) | 0.713 |  | 1.08 (0.63-1.84) | 0.793 |
| AA | 269 (20.8) | 1.08 (0.57-2.05) | 0.820 |  | 0.99 (0.50-1.96) | 0.985 |
| rs1043291 |  |  |  |  |  |  |
| AA | 483 (37.4) | 1 (ref) |  |  | 1 (ref) |  |
| AG | 602 (46.6) | 0.84 (0.52-1.36) | 0.483 |  | 0.94 (0.57-1.56) | 0.811 |
| GG | 208 (16.1) | 0.94 (0.50-1.77) | 0.854 |  | 1.04 (0.53-2.06) | 0.909 |
| rs17104965 |  |  |  |  |  |  |
| GG | 719 (55.5) | 1 (ref) |  |  | 1 (ref) |  |
| AG | 473 (36.5) | 0.59 (0.35-0.97) | 0.039 |  | 0.55 (0.32-0.94) | 0.029 |
| AA | 104 (8.0) | 0.83 (0.36-1.92) | 0.656 |  | 0.91 (0.38-2.16) | 0.830 |
| rs12705977 |  |  |  |  |  |  |
| CC | 951 (73.4) | 1 (ref) |  |  | 1 (ref) |  |
| AC | 316 (24.4) | 1.08 (0.65-1.82) | 0.761 |  | 1.11 (0.64-1.92) | 0.721 |
| AA | 29 (2.2) | 2.69 (0.84-8.61) | 0.096 |  | 3.50 (1.06-11.55) | 0.040 |
| rs1395119 |  |  |  |  |  |  |
| AA | 811 (62.5) | 1 (ref) |  |  | 1 (ref) |  |
| AT | 421 (32.5) | 0.88 (0.55-1.42) | 0.600 |  | 1.01 (0.62-1.65) | 0.981 |
| TT | 65 (5.0) | 0.46 (0.11-1.89) | 0.280 |  | 0.26 (0.04-1.88) | 0.181 |
| rs216598 |  |  |  |  |  |  |
| GG | 424 (32.7) | 1 (ref) |  |  | 1 (ref) |  |
| AG | 645 (49.7) | 0.70 (0.42-1.15) | 0.153 |  | 0.79 (0.46-1.34) | 0.382 |
| AA | 228 (17.6) | 1.15 (0.64-2.06) | 0.649 |  | 1.31 (0.71-2.43) | 0.389 |
| rs2822638 |  |  |  |  |  |  |
| AA | 462 (35.6) | 1 (ref) |  |  | 1 (ref) |  |
| AG | 593 (45.8) | 1.04 (0.64-1.71) | 0.868 |  | 0.99 (0.59-1.68) | 0.982 |
| GG | 241 (18.6) | 1.13 (0.61-2.09) | 0.702 |  | 1.15 (0.60-2.19) | 0.675 |
| rs444927 |  |  |  |  |  |  |
| GG | 531 (41.0) | 1 (ref) |  |  | 1 (ref) |  |
| AG | 598 (46.2) | 1.54 (0.96-2.48) | 0.074 |  | 1.41 (0.86-2.32) | 0.176 |
| AA | 166 (12.8) | 0.66 (0.27-1.61) | 0.363 |  | 0.68 (0.28-1.66) | 0.397 |
| rs1056796 |  |  |  |  |  |  |
| CC | 335 (25.9) | 1 (ref) |  |  | 1 (ref) |  |
| AC | 677 (52.3) | 1.61 (0.90-2.88) | 0.106 |  | 1.61 (0.88-2.97) | 0.125 |
| AA | 283 (21.9) | 1.34 (0.67-2.68) | 0.410 |  | 1.60 (0.78-3.28) | 0.196 |

^#^ Adjusted for age at diagnosis, education, occupation, age at menarche, number of live births, breastfeeding duration, abortion, menopause, TNM stage, tumor size, histopathologic classification, grade, lymph node, ER, PR, and HER2.
