# Supplementary material for: SNPs within microRNA binding sites and the prognosis of breast cancer
Source: Aging (Albany NY). 2021 Feb 26;13(5):7465–80. doi: 10.18632/aging.202612 (PMC7993692; doi:10.18632/aging.202612)
Supplement: Supplementary Table 3 [file aging-13-202612-s004.docx]

**Supplementary Table 3. Association between the SNP rs10878441 and breast cancer OS stratified by clinical characteristics.**

| Variables | Genotype | *N* (%) | Univariate | |  | Multivariate | |
| --- | --- | --- | --- | --- | --- | --- | --- |
|  |  |  | HR (95% CI) | *P* |  | HR (95% CI) | *P^#^* |
| Age |  |  |  |  |  |  |  |
| ≤50 | AA | 431 | 1 (ref) |  |  | 1 (ref) |  |
|  | AC | 537 | 0.75 (0.45-1.24) | 0.261 |  | 0.80 (0.38-1.68) | 0.549 |
|  | CC | 179 | 1.50 (0.84-2.69) | 0.174 |  | 1.82 (0.78-4.28) | 0.167 |
|  | Additive model | 1147 | 1.15 (0.83-1.59) | 0.393 |  | 1.28 (0.80-2.04) | 0.301 |
|  | Dominant model | 1147 | 0.92 (0.58-1.46) | 0.734 |  | 1.02 (0.52-2.01) | 0.946 |
|  | Recessive model | 1147 | **1.75 (1.03-2.97)** | **0.037** |  | 2.07 (0.97-4.42) | 0.062 |
| >50 | AA | 543 | 1 (ref) |  |  | 1 (ref) |  |
|  | AC | 684 | **1.51 (1.04-2.17)** | **0.029** |  | **1.87 (1.08-3.22)** | **0.025** |
|  | CC | 218 | **1.81 (1.15-2.86)** | **0.011** |  | **2.63 (1.34-5.17)** | **0.005** |
|  | Additive model | 1445 | **1.36 (1.09-1.70)** | **0.006** |  | **1.64 (1.18-2.27)** | **0.003** |
|  | Dominant model | 1445 | **1.58 (1.12-2.24)** | **0.010** |  | **2.03 (1.21-3.42)** | **0.008** |
|  | Recessive model | 1445 | 1.42 (0.96-2.10) | 0.079 |  | **1.80 (1.02-3.18)** | **0.043** |
| TNM |  |  |  |  |  |  |  |
| 0-Ⅱa | AA | 585 | 1 (ref) |  |  | 1 (ref) |  |
|  | AC | 733 | 1.43 (0.85-2.41) | 0.182 |  | 1.51 (0.72-3.15) | 0.279 |
|  | CC | 242 | **2.92 (1.65-5.17)** | **<0.001** |  | **3.19 (1.43-7.09)** | **0.004** |
|  | Additive model | 1560 | **1.72 (1.28-2.32)** | **<0.001** |  | **1.81 (1.19-2.73)** | **0.005** |
|  | Dominant model | 1560 | **1.79 (1.10-2.91)** | **0.019** |  | 1.93 (0.97-3.83) | 0.061 |
|  | Recessive model | 1560 | **2.36 (1.49-3.74)** | **<0.001** |  | **2.48 (1.32-4.68)** | **0.005** |
| Ⅱb-Ⅳ | AA | 282 | 1 (ref) |  |  | 1 (ref) |  |
|  | AC | 358 | 1.12 (0.77-1.63) | 0.554 |  | 1.54 (0.89-2.65) | 0.125 |
|  | CC | 110 | 1.35 (0.81-2.23) | 0.248 |  | 1.88 (0.89-4.00) | 0.099 |
|  | Additive model | 750 | 1.15 (0.90-1.47) | 0.256 |  | 1.40 (0.98-2.00) | 0.062 |
|  | Dominant model | 750 | 1.17 (0.82-1.67) | 0.380 |  | 1.61 (0.95-2.70) | 0.075 |
|  | Recessive model | 750 | 1.27 (0.80-2.00) | 0.314 |  | 1.48 (0.75-2.90) | 0.254 |
| Tumor size |  |  |  |  |  |  |  |
| ≤2.5cm | AA | 553 | 1 (ref) |  |  | 1 (ref) |  |
|  | AC | 680 | 1.08 (0.69-1.67) | 0.747 |  | 1.32 (0.73-2.41) | 0.358 |
|  | CC | 213 | 1.65 (0.97-2.81) | 0.067 |  | **2.44 (1.21-4.89)** | **0.012** |
|  | Additive model | 1446 | 1.26 (0.96-1.66) | 0.103 |  | **1.55 (1.08-2.22)** | **0.018** |
|  | Dominant model | 1446 | 1.21 (0.81-1.82) | 0.358 |  | 1.58 (0.91-2.75) | 0.107 |
|  | Recessive model | 1446 | 1.58 (0.99-2.53) | 0.057 |  | **2.08 (1.14-3.78)** | **0.017** |
| >2.5cm | AA | 243 | 1 (ref) |  |  | 1 (ref) |  |
|  | AC | 339 | 1.12 (0.71-1.77) | 0.620 |  | 1.49 (0.77-2.86) | 0.236 |
|  | CC | 110 | 1.40 (0.79-2.48) | 0.247 |  | 1.58 (0.69-3.64) | 0.281 |
|  | Additive model | 692 | 1.18 (0.88-1.56) | 0.265 |  | 1.28 (0.86-1.90) | 0.225 |
|  | Dominant model | 392 | 1.19 (0.78-1.83) | 0.422 |  | 1.51 (0.81-2.81) | 0.195 |
|  | Recessive model | 692 | 1.31 (0.79-2.17) | 0.291 |  | 1.24 (0.61-2.55) | 0.551 |
| ER |  |  |  |  |  |  |  |
| Negative | AA | 382 | 1 (ref) |  |  | 1 (ref) |  |
|  | AC | 483 | 1.11 (0.74-1.67) | 0.603 |  | 1.45 (0.81-2.61) | 0.214 |
|  | CC | 146 | 1.67 (1.00-2.78) | 0.052 |  | 1.88 (0.87-4.04) | 0.108 |
|  | Additive model | 1011 | 1.26 (0.97-1.64) | 0.079 |  | 1.38 (0.95-2.00) | 0.092 |
|  | Dominant model | 1011 | 1.23 (0.84-1.80) | 0.281 |  | 1.54 (0.88-2.70) | 0.135 |
|  | Recessive model | 1011 | 1.57 (0.99-2.47) | 0.054 |  | 1.48 (0.77-2.86) | 0.240 |
| Positive | AA | 558 | 1 (ref) |  |  | 1 (ref) |  |
|  | AC | 709 | 1.35 (0.87-2.09) | 0.187 |  | 1.36 (0.71-2.60) | 0.352 |
|  | CC | 240 | **1.83 (1.08-3.11)** | **0.025** |  | **2.40 (1.15-4.98)** | **0.020** |
|  | Additive model | 1507 | **1.35 (1.04-1.76)** | **0.025** |  | **1.54 (1.06-2.24)** | **0.024** |
|  | Dominant model | 1507 | 1.47 (0.97-2.22) | 0.069 |  | 1.63 (0.90-2.96) | 0.109 |
|  | Recessive model | 1507 | 1.54 (0.98-2.43) | 0.062 |  | **2.02 (1.08-3.77)** | **0.028** |
| PR |  |  |  |  |  |  |  |
| Negative | AA | 429 | 1 (ref) |  |  | 1 (ref) |  |
|  | AC | 559 | 1.27 (0.87-1.84) | 0.218 |  | 1.24 (0.72-2.11) | 0.441 |
|  | CC | 173 | 1.43 (0.87-2.35) | 0.159 |  | 1.90 (0.96-3.74) | 0.065 |
|  | Additive model | 1161 | 1.21 (0.95-1.53) | 0.124 |  | 1.36 (0.96-1.92) | 0.080 |
|  | Dominant model | 1161 | 1.30 (0.91-1.86) | 0.144 |  | 1.37 (0.82-2.27) | 0.228 |
|  | Recessive model | 1161 | 1.25 (0.80-1.93) | 0.330 |  | 1.67 (0.93-3.00) | 0.089 |
| Positive | AA | 511 | 1 (ref) |  |  | 1 (ref) |  |
|  | AC | 633 | 1.17 (0.71-1.91) | 0.539 |  | 1.63 (0.79-3.39) | 0.190 |
|  | CC | 213 | **2.25 (1.29-3.92)** | **0.004** |  | **2.66 (1.16-6.12)** | **0.022** |
|  | Additive model | 1357 | **1.48 (1.11-1.99)** | **0.009** |  | **1.63 (1.08-2.47)** | **0.021** |
|  | Dominant model | 1357 | 1.42 (0.90-2.23) | 0.130 |  | 1.90 (0.96-3.75) | 0.064 |
|  | Recessive model | 1357 | **2.06 (1.28-3.31)** | **0.003** |  | **2.02 (1.00-4.09)** | **0.049** |
| HER2 |  |  |  |  |  |  |  |
| Negative | AA | 649 | 1 (ref) |  |  | 1 (ref) |  |
|  | AC | 854 | 1.15 (0.79-1.68) | 0.473 |  | 1.26 (0.75-2.12) | 0.391 |
|  | CC | 284 | **1.78 (1.14-2.79)** | **0.012** |  | **1.94 (1.04-3.62)** | **0.038** |
|  | Additive model | 1787 | 1.26 (0.75-2.12) | 0.391 |  | **1.38 (1.01-1.90)** | **0.046** |
|  | Dominant model | 1787 | 1.31 (0.92-1.86) | 0.140 |  | 1.42 (0.87-2.31) | 0.159 |
|  | Recessive model | 1787 | **1.64 (1.11-2.43)** | **0.012** |  | 1.70 (0.99-2.90) | 0.054 |
| Positive | AA | 206 | 1 (ref) |  |  | 1 (ref) |  |
|  | AC | 259 | 1.59 (0.91-2.77) | 0.104 |  | 1.75 (0.79-3.88) | 0.170 |
|  | CC | 76 | 1.56 (0.73-3.36) | 0.253 |  | **3.10 (1.15-8.39)** | **0.026** |
|  | Additive model | 541 | 1.30 (0.92-1.84) | 0.143 |  | **1.76 (1.07-2.89)** | **0.026** |
|  | Dominant model | 541 | 1.58 (0.93-2.70) | 0.093 |  | 2.00 (0.94-4.27) | 0.074 |
|  | Recessive model | 541 | 1.19 (0.60-2.33) | 0.622 |  | 2.20 (0.95-5.09) | 0.065 |
| Grade |  |  |  |  |  |  |  |
| I | AA | 70 | 1 (ref) |  |  | 1 (ref) |  |
|  | AC | 85 | 1.00 (0.14-7.17) | 0.997 |  | **-** | 0.651 |
|  | CC | 26 | **9.95 (1.89-52.42)** | **0.007** |  | **-** | 0.646 |
|  | Additive model | 181 | **3.98 (1.48-10.69)** | **0.006** |  | **10.75 (1.48-78.27)** | **0.019** |
|  | Dominant model | 181 | 2.76 (0.57-13.41) | 0.207 |  | - | 0.912 |
|  | Recessive model | 181 | **9.93 (2.63-37.53)** | **0.001** |  | 10.82 (0.85-137.33) | 0.066 |
| II | AA | 509 | 1 (ref) |  |  | 1 (ref) |  |
|  | AC | 650 | 1.45 (0.06-2.18) | 0.078 |  | 1.56 (0.93-2.60) | 0.091 |
|  | CC | 208 | **2.26 (1.39-3.66)** | **0.001** |  | **2.60 (1.40-4.82)** | **0.002** |
|  | Additive model | 1367 | **1.50 (1.17-1.91)** | **0.001** |  | **1.61 (1.18-2.20)** | **0.003** |
|  | Dominant model | 1367 | **1.64 (1.11-2.40)** | **0.012** |  | **1.76 (1.08-2.88)** | **0.022** |
|  | Recessive model | 1367 | **1.81 (1.21-2.73)** | **0.004** |  | **1.96 (1.18-3.26)** | **0.009** |
| III | AA | 123 | 1 (ref) |  |  | 1 (ref) |  |
|  | AC | 126 | 1.04 (0.52-2.07) | 0.920 |  | 1.04 (0.39-2.80) | 0.940 |
|  | CC | 49 | 0.87 (0.34-2.22) | 0.768 |  | 1.29 (0.34-4.84) | 0.709 |
|  | Additive model | 298 | 0.95 (0.62-1.47) | 0.826 |  | 1.12 (0.58-2.13) | 0.742 |
|  | Dominant model | 298 | 0.98 (0.52-1.87) | 0.961 |  | 1.10 (0.43-2.77) | 0.846 |
|  | Recessive model | 298 | 0.85 (0.36-2.04) | 0.721 |  | 1.26 (0.38-4.21) | 0.707 |
| Histopathologic |  |  |  |  |  |  |  |
| non-IDC | AA | 305 | 1 (ref) |  |  | 1 (ref) |  |
|  | AC | 370 | 1.39 (0.75-2.55) | 0.294 |  | - | 0.950 |
|  | CC | 105 | 1.23 (0.51-2.97) | 0.649 |  | - | 0.932 |
|  | Additive model | 775 | 1.16 (0.78-1.73) | 0.458 |  | - | 0.884 |
|  | Dominant model | 775 | 1.35 (0.75-2.43) | 0.315 |  | - | 0.851 |
|  | Recessive model | 775 | 1.02 (0.46-2.26) | 0.970 |  | - | 0.894 |
| IDC | AA | 669 | 1 (ref) |  |  | 1 (ref) |  |
|  | AC | 849 | 1.12 (0.80-1.56) | 0.517 |  | 1.41 (0.91-2.17) | 0.126 |
|  | CC | 292 | **1.74 (1.17-2.59)** | **0.006** |  | **2.15 (1.25-3.69)** | **0.005** |
|  | Additive model | 1810 | **1.30 (1.06-1.60)** | **0.012** |  | **146 (1.12-1.92)** | **0.006** |
|  | Dominant model | 1810 | 1.27 (0.93-1.74) | 0.131 |  | **1.56 (1.03-2.36)** | **0.034** |
|  | Recessive model | 1810 | **1.64 (1.16-2.31)** | **0.005** |  | **1.75 (1.10-2.77)** | **0.017** |
| Lymph node |  |  |  |  |  |  |  |
| Negative | AA | 745 | 1 (ref) |  |  | 1 (ref) |  |
|  | AC | 946 | 1.55 (0.98-2.44) | 0.062 |  | 1.74 (0.89-3.41) | 0.105 |
|  | CC | 298 | **2.56 (1.51-4.34)** | **<0.001** |  | **2.85 (1.33-6.10)** | **0.007** |
|  | Additive model | 1989 | **1.60 (1.23-2.09)** | **0.001** |  | **1.69 (1.16-2.46)** | **0.007** |
|  | Dominant model | 1989 | **1.78 (1.16-2.74)** | **0.008** |  | **2.02 (1.07-3.80)** | **0.029** |
|  | Recessive model | 1989 | **1.97 (1.27-3.04)** | **0.002** |  | **2.03 (1.10-3.75)** | **0.024** |
| Positive | AA | 215 | 1 (ref) |  |  | 1 (ref) |  |
|  | AC | 251 | 1.05 (0.70-1.56) | 0.829 |  | 1.37(0.77-2.46) | 0.286 |
|  | CC | 89 | 1.23 (0.72-2.09) | 0.447 |  | 2.03 (0.95-4.36) | 0.068 |
|  | Additive model | 555 | 1.10 (0.85-1.42) | 0.487 |  | 1.42 (0.98-2.06) | 0.067 |
|  | Dominant model | 555 | 1.09 (0.75-1.59) | 0.651 |  | 1.51 (0.88-2.62) | 0.138 |
|  | Recessive model | 555 | 1.20 (0.74-1.94) | 0.460 |  | 1.72 (0.87-3.41) | 0.122 |

^#^ Adjusted for age at diagnosis, education, occupation, age at menarche, number of live births, breastfeeding duration, abortion, menopause, TNM stage, tumor size, histopathologic classification, grade, lymph node, ER, PR, and HER2.
